# Supplementary material for: Synthesis of a covalent organic framework with hetero-environmental pores and its medicine co-delivery application
Source: Nat Commun. 2023 Sep 28;14:6049. doi: 10.1038/s41467-023-41622-x (PMC10539374; doi:10.1038/s41467-023-41622-x)
Supplement: Supplementary file 1 — Supplementary Information [file 41467_2023_41622_MOESM1_ESM.pdf]

Supplementary Information for

Synthesis of a covalent organic framework with hetero-environmental pores and its medicine co-delivery application

Wenyan Ji,[1,2,#] Pai Zhang,[3,#] Gungyuan Feng,[2,#] Yuan-Zhe Cheng,[1,4], Tian-Xiong Wang,[1,4] Daqiang Yuan,[5] Ruitao Cha,*[,3] Xuesong Ding,*[,1] Shengbin Lei,*[,2,6] Bao-Hang Han*[,1,4]

[1] *CAS Key Laboratory of Nanosystem and Hierarchical Fabrication, CAS Center for Excellence in Nanoscience, National Center for Nanoscience and Technology, Beijing 100190, China*

[2] *Department of Chemistry, School of Science & Collaborative Innovation Center of Chemical Science and Engineering (Tianjin), Tianjin University, Tianjin 300072, China*

[3] *CAS Key Laboratory for Biomedical Effects of Nanomaterials and Nanosafety, CAS Center for Excellence in Nanoscience, National Center for Nanoscience and Technology, Beijing, 100190, China*

[4] *University of Chinese Academy of Sciences, Beijing 100049, China*

[5] State Key Laboratory of Structure Chemistry, Fujian Institute of Research on the Structure of Matter, Chinese Academy of Sciences (CAS), Fuzhou 350002, China

[6] Tianjin Key Laboratory of Molecular Optoelectronic Science, Tianjin University, Tianjin 300072, China

Tel.: +86 10 8254 5576. E-mail: hanbh@nanoctr.cn

E-mail: shengbin.lei@tju.edu.cn

Tel.: +86 10 8254 5708. E-mail: dingxs@nanoctr.cn

Tel.: +86 10 8254 5575. E-mail: chart@nanoctr.cn

Table of contents:

Supplementary Methods

Supplementary Figures

Supplementary Tables

Supplementary References

Supplementary Methods

Materials

Tetrahydrofuran (THF), nitrobenzene, chloroform, dichloromethane, petroleum ether, ethyl acetate, dimethyl sulphoxide (DMSO), acetone, *o*-dichlorobenzene (*o*-DCB), methanol, absolute alcohol, lactic acid, hydrochloric acid, and other commonly used organic solvents were purchased from Sinopharm Chemical Reagent Co. Pyrrole, *p*-nitrobenzaldehyde, 4-heptylphenol, 1-bromo-2-(2-methoxyethoxy)ethane, 4-formylphenylboronic acid, bis(triphenylphosphine)palladium(II) dichloride, bromine, hydroquinone, and 1-bromoheptane were purchased from InnoChom Science & Technology Co. Ltd., Beijing. Polycaprolactone (PCL, MW = 80000) was obtained from Sigma-Aldrich. Gelatin (type A, 300 bloom) was obtained from Aladdin. The levofloxacin (Lev) and vancomycin hydrochloride (Van) were obtained from Beijing Solarbio Science & Technology Co., Ltd., China. Methicillin-resistant *S. aureus* (MRSA) was obtained from a local hospital in China. Ultrapure water (18 MΩ cm) was produced through a Millipore-ELIX water purification system. Nitrogen (N_2, 99.999 % purity) was purchased from Beijing Haike Yuanchang Gas Co. Ltd., China. All chemicals and solvents were reagent grade and used without further purification, unless with particularly noted.

Synthesis of Monomers

Synthesis of 5,10,15,20-Tetrakis(4-aminophenyl)porphyrin (TAPP).

5,10,15,20-Tetrakis(4-nitrophenyl)porphyrin (TNPP) was synthesized according to the following procedure.[1,2] The solution of *p*-nitrobenzaldehyde (8.32 g, 55 mmol) with 50 mL nitrobenzene and 14 mL lactic acid was heated to 160 °C, and stirred within 40 min. After a 24 mL nitrobenzene solution containing 3.68 g (3.8 mL, 55 mmol) pyrrole being dropwise added in, the reaction was continued for 5 h. After the reaction system being cooled to room temperature, 60 mL of methanol was added in. The mixture was stirred for 10 min, and stored at –4 °C overnight to obtain precipitated purple crystals. The precipitate was filtered and washed with methanol until the effluent became colorless, and then was collected and dried in vacuum at 60 °C to obtain 2.2 g of bright purple TNPP. 2.2 g of TNPP was dissolved in 100 mL of concentrated HCl solution, and 25 mL of concentrated HCl solution containing 9.4 g of $SnCl_2$ was added dropwise within 10 min at room temperature. The reaction was continued for 2.5 h, and then the temperature was raised to 70 °C for 30 min. The reaction mixture was cooled in an ice water bath, and the dark green tetrakis(4-aminophenyl)porphyrin hydrochloride solid was filtered. The hydrochloride solid was dispersed in 200 mL deionized water, then concentrated ammonia water was added to neutralize the solution until pH = 9. The solid is filtered and dried in vacuum oven at 60 °C. 2.5 g purple solid were obtained. The purple solid was extracted by Soxhlet extractor with chloroform for 3 d, and then a bright purple TAPP (1.5 g) was obtained

with the 80% yield. ^{1}H NMR (400 MHz, DMSO) δ = 8.89 (s, 8H), 7.87 (d, J = 8.0, 8H), 7.02 (d, J = 8.0, 8H), 5.58 (s, 8H). HRMS (m/z): $[M]^+$ calcd. for $C_{44}H_{34}N_8$: 674.29; found: 675.2963 $[M + H]^+$.

Synthesis of 1-Heptyl-4-(2-(2-methoxyethoxy)ethoxy)benzene (DEG-HEP-H).

DEG-HEP-H was synthesized following the procedure. To a 100 mL round-bottom flask was charged with 4-heptylphenol (8.46 g, 44 mmol) and KOH (9.88 g, 176 mmol). DMSO (20 mL) was added to the flask and stirred vigorously for 30 min. 1-Bromo-2-(2-methoxyethoxy)ethane (7.40 mL, 55 mmol) and sodium iodide (catalytic amount) were added in, and the system was subsequently stirred at room temperature for 10 h. Then the reaction mixture was poured into water (400 mL) and extracted with dichloromethane. The organic layers were collected, washed with water until the water layers became neutral, dried with $MgSO_4$, filtered, and evaporated by rotary evaporation. The oil crude products were purified by column chromatography (petroleum ether and ethyl acetate as the eluents) to give the DEG-HEP-Ph (yield 83%). ^{1}H NMR (400 MHz, $CDCl_3$) δ = 7.07 (d, *J* = 8.5 Hz, 2H), 6.83 (d, *J* = 8.6 Hz, 2H), 4.16–4.07 (m, 2H), 3.87–3.81 (m, 2H), 3.71 (dd, *J* = 5.6, 3.7 Hz, 2H), 3.57 (dd, *J* = 5.6, 3.7 Hz, 2H), 3.39 (s, 3H), 2.59–2.48 (m, 2H), 1.56 (d, *J* = 7.3 Hz, 2H), 1.29 (dd, *J* = 8.2, 4.1 Hz, 8H), 0.87 (s, 3H).

Synthesis of 1,4-Dibromo-2-heptyl-5-(2-(2-methoxyethoxy)ethoxy)benzene (DEG-HEP-Br).

DEG-HEP-Br was synthesized following the procedure. To a 250 mL round-bottom flask was charged with DEG-HEP-H (3.80 g, 11.2 mmol). After the flask being evacuated and filled with nitrogen, 40 mL CCl_4 (90 mL) were added into. The reaction system was stirred in an ice/salt bath until –6 °C, and the catalytic amount of iron powder was added in. Bromine (1.10 mL, 22.4 mmol) in the 20 mL CCl_4 were dropwise added in flask by an addition funnel over 10 min. The exhaust gas was vented through the saturated aqueous Na_2SO_3 solution. After being reacted at 6 h, 1.10 mL bromine were added again, and the reaction system was stirred in an ice/salt bath overnight. The reaction was quenched with the saturated aqueous Na_2SO_3 solution (100 mL) and stirred vigorously until colorless. The reaction mixture was extracted with dichloromethane. The organic layers were collected, washed with water, dried with $MgSO_4$, filtered, and evaporated by rotary evaporation to give colorless transparent liquid. The crude products were purified by column chromatography (petroleum ether/ethyl acetate = 10/1 as the eluent) to give the DEG-HEP-Br (yield 50%). ^{1}H NMR (400 MHz, $CDCl_3$) δ = 7.38 (s, 1H), 7.11 (s, 1H), 4.20–4.14 (m, 2H), 3.96–3.90 (m, 2H), 3.79 (dd, J = 5.5, 3.7 Hz, 2H), 3.60 (dd, J = 5.5, 3.7 Hz, 2H), 3.42 (s, 3H), 2.71–2.56 (m, 2H), 1.69–1.51 (m, 2H), 1.34 (dd, J = 10.0, 7.3 Hz, 8H), 0.91 (t, J = 6.8 Hz, 3H).

Synthesis of 2'-Heptyl-5'-(2-(2-methoxyethoxy)ethoxy)-[1,4'-terphenyl]-4,4''-dicarbaldehyde (DEG-HEP-CHO).

DEG-HEP-CHO was synthesized following the procedure. To a 250 mL round-bottom flask was charged with DEG-HEP-Br (1.06 g, 2.35 mmol) and 4-formylphenylboronic acid (1.06 g, 7.05 mmol). After the flask being evacuated and filled with nitrogen, 70 mL THF were added into. Aqueous solution of potassium carbonate (25.0 mL, 2.0 M) and bis(triphenylphosphine)palladium(II) dichloride (200 mg) were successively added under nitrogen atmosphere. The reaction system was stirred at 100 °C for 12 h. After being cooled to room temperature, the THF was evaporated by rotary evaporation, and the residual solution was then extracted with dichloromethane. The organic layers were collected, washed with water until the water layers became neutral, dried with $MgSO_4$, filtered, and evaporated by rotary evaporation. The oil crude products were purified by column chromatography (petroleum ether/ethyl acetate = 5/1 and petroleum ether/ethyl acetate = 1/1 as the eluents) to give DEG-HEP-CHO (yield 81%). ^{1}H NMR (400 MHz, $CDCl_3$) δ = 10.09 (d, J = 11.5 Hz, 2H), 7.96 (dd, J = 12.9, 8.2 Hz, 4H), 7.83 (d, J = 8.2 Hz, 2H), 7.53 (d, J = 8.0 Hz, 2H), 7.30 (s, 1H), 6.84 (s, 1H), 4.15 (dd, J = 8.4, 3.3 Hz, 2H), 3.83–3.74 (m, 2H), 3.61 (dd, J = 5.7, 3.5 Hz, 2H), 3.50 (dd, J = 5.6, 3.5 Hz, 2H), 3.36 (s, 3H), 2.60–2.50 (m, 2H), 1.25 (dd, J = 18.5, 11.4 Hz, 10H), 0.84 (t, J = 7.0 Hz, 3H). ^{13}C NMR (400 MHz, $CDCl_3$) δ = 191.99, 153.64, 147.98, 144.64, 141.54, 135.21, 134.9, 133.08, 131.90, 130.28, 129.87, 129.64, 129.32, 114.30, 71.98, 70.78, 69.63, 68.49, 59.07, 32.22, 31.62, 29.29, 28.91, 22.58, 14.04. HRMS (m/z): $[M]^+$ calcd. for $C_{32}H_{35}O_5$: 502.27; found, 503.2663 $[M + H]^+$.

Synthesis of 1,4-Bis(2-(2-methoxyethoxy)ethoxy)benzene (DEG-H).

DEG-H was synthesized according to the following procedure.[3,4] To a 100 mL round-bottom flask was charged with hydroquinone (1.12 g, 10.13 mmol) and KOH (4.55 g, 81.04 mmol). 50 mL DMSO were added to the flask and stirred vigorously for 30 min. 1-bromo-2-(2-methoxyethoxy)ethane (3.00 ml, 22.30 mmol) and sodium iodide (catalytic amount) were added in, and the system was subsequently stirred at room temperature for 10 h. Then the reaction mixture was poured into water (400 mL), and extracted with dichloromethane. The organic layers were collected, washed with water until the water layers became neutral, dried with $MgSO_4$, filtered, and evaporated by rotary evaporation. The oil crude products were purified by column chromatography (petroleum ether and ethyl acetate as the eluents) to give the DEG-H (yield 93%). ^{1}H NMR (400 MHz, $CDCl_3$) δ = 6.83 (s, 2H), 4.12–4.01 (m, 2H), 3.88–3.78 (m, 2H), 3.71 (dd, J = 5.6, 3.7 Hz, 2H), 3.57 (dd, J = 5.6, 3.7 Hz, 2H), 3.39 (s, 3H).

Synthesis of 1,4-Dibromo-2,5-bis(2-(2-methoxyethoxy)ethoxy)benzene (DEG-Br).

DEG-Br was synthesized according to the following procedure.[5] To a 250 mL round-bottom flask was charged with DEG-H (1.85 g, 5.88 mmol). After the flask being evacuated and filled with nitrogen, 20 mL CCl_4 were added into. The reaction system was stirred in an ice/salt bath until –6 °C, and the catalytic amount of iron powder was added in. Bromine (0.66 mL, 12.9 mmol) in the 10 mL CCl_4 were successively dropwise added in flask by an addition funnel over 10 min. The exhaust

gas was vented through the saturated aqueous Na_2SO_3 solution. The reaction system was stirred in an ice/salt bath overnight, and then quenched with the saturated aqueous Na_2SO_3 solution (100 mL) and stirred vigorously until colorless. The reaction mixture was extracted with dichloromethane. The organic layers were collected, washed with water, dried with $MgSO_4$, filtered, and evaporated by rotary evaporation to give colorless transparent liquid. The crude products were purified by column chromatography (petroleum ether/ethyl acetate = 2/1 as the eluent) to give the DEG-Br white soild (yield 70%). ^{1}H NMR (400 MHz, $CDCl_3$) δ = 7.16 (s, 2H), 4.17–4.11 (m, 4H), 3.91–3.84 (m, 4H), 3.80–3.73 (m, 4H), 3.61–3.54 (m, 4H), 3.40 (s, 6H).

Synthesis of 2',5'-Bis(2-(2-methoxyethoxy)ethoxy)-[1,1':4',1''-terphenyl]-4,4''-dicarbaldehyde (DEG-CHO).

DEG-CHO was synthesized according to the following procedure.[6] To a 250 mL round-bottom flask was charged with DEG-Br (0.70 g, 1.49 mmol) and 4-formylphenylboronic acid (0.67 g, 4.47 mmol). After the flask being evacuated and filled with nitrogen, 50 mL THF were added into. Aqueous potassium carbonate solution (15.0 mL, 2.0 M) and bis(triphenylphosphine)palladium(II) dichloride (100 mg) were successively added in under nitrogen atmosphere. The reaction system was stirred at 100 °C for 12 h. After being cooled to room temperature, the THF was evaporated by rotary evaporation, and the residual solution was then extracted with dichloromethane. The organic layers were collected, washed with water until the water layers became neutral, dried with $MgSO_4$, filtered, and evaporated by rotary

evaporation. The oil crude products were purified by column chromatography (petroleum ether/ethyl acetate = 10/1 as the eluent) to give DEG-CHO (yield 80%). ^{1}H NMR (400 MHz, $CDCl_3$) δ = 10.07 (s, 2H), 7.94 (d, J = 8.2 Hz, 4H), 7.81 (d, J = 8.2 Hz, 4H), 7.05 (s, 2H), 4.17–4.12 (m, 4H), 3.79–3.73 (m, 4H), 3.60 (dd, J = 5.6, 3.5 Hz, 4H), 3.50 (dd, J = 5.7, 3.5 Hz, 4H), 3.35 (s, 6H). ^{13}C NMR (404 MHz, $CDCl_3$) δ = 192.00, 150.47, 144.27, 135.10, 130.56, 130.20, 129.46, 116.41, 71.97, 70.76, 69.74, 69.31, 59.07. HRMS (m/z): $[M]^+$ calcd. for $C_{30}H_{34}O_8$: 522.23; found: 523.2337 $[M + H]^+$.

Synthesis of 1,4-Bis(heptyloxy)benzene (HEP-H).

To a 100 mL round-bottom flask was charged with hydroquinone (1.90 g, 17.30 mmol) and KOH (7.77 g, 138.40 mmol). 50 mL DMSO were added to the flask and stirred vigorously for 30 min. 1-bromoheptane (6.00 mL, 38.20 mmol) and sodium iodide (catalytic amount) were added in, and the system was subsequently stirred at room temperature for 10 h. Then the reaction mixture was poured into water (400 mL), and extracted with dichloromethane. The organic layers were collected, washed with water until the water layers became neutral, dried with $MgSO_4$, filtered, and evaporated by rotary evaporation. The oil crude products were purified by column chromatography (petroleum ether and ethyl acetate as the eluents) to give the HEP-Ph (yield 92%). ^{1}H NMR (400 MHz, $CDCl_3$) δ = 6.83 (s, 2H), 3.91 (t, J = 6.6 Hz, 2H), 1.91–1.65 (m, 2H), 1.50–1.25 (m, 8H), 0.91 (t, J = 6.8 Hz, 3H).

Synthesis of 1,4-Dibromo-2,5-bis(2-(2-methoxyethoxy)ethoxy)benzene (HEP-Br).

To a 250 mL round-bottom flask was charged with HEP-H (1.62 g, 5.28 mmol). After the flask being evacuated and filled with nitrogen, 20 mL CCl_4 were added into. The reaction system was stirred in an ice/salt bath until to –6 °C, and the catalytic amount of iron powder was added in. Bromine (0.60 mL, 11.62 mmol) in the 10 mL CCl_4 were successively dropwise added in flask by an addition funnel over 10 min. The exhaust gas was vented through the saturated aqueous Na_2SO_3 solution. The reaction system was stirred in an ice/salt bath overnight, and then quenched with the saturated aqueous Na_2SO_3 solution (100 mL) and stirred vigorously until colorless. The reaction mixture was extracted with dichloromethane. The organic layers were collected, washed with water, dried with $MgSO_4$, filtered, and evaporated by rotary evaporation to give colorless transparent liquid. The crude products were purified by column chromatography (petroleum ether/ethyl acetate = 20/1 as the eluent) to give the HEP-Br white soild (yield 68%). ^{1}H NMR (400 MHz, $CDCl_3$) δ = 7.08 (s, 1H), 3.95 (t, J = 6.5 Hz, 2H), 1.86–1.75 (m, 2H), 1.50–1.27 (m, 8H), 0.89 (t, J = 6.8 Hz, 3H).

Synthesis of 2',5'-Bis(heptyloxy)-[1,1':4',1''-terphenyl]-4,4''-dicarbaldehyde (HEP-CHO).

To a 250 mL round-bottom flask was charged with HEP-Br (0.70 g, 1.51 mmol,) and 4-formylphenylboronic acid (0.68 g, 4.53 mmol). After the flask being evacuated and filled with nitrogen, 50 mL THF were added into. Aqueous potassium carbonate solution (15.0 mL, 2.0 M) and bis(triphenylphosphine)palladium(II) dichloride (100

mg) were successively added in under nitrogen atmosphere. The reaction was stirred at 100 °C for 12 h. After being cooled to room temperature, the THF was evaporated by rotary evaporation, and the residual solution was then extracted with dichloromethane. The organic layers were collected, washed with water until the water layers became neutral, dried with $MgSO_4$, filtered, and evaporated by rotary evaporation. The oil crude products were purified by column chromatography (petroleum ether/ethyl acetate = 1/1 as the eluent) to give HEP-CHO (yield 82%). ^{1}H NMR (400 MHz, $CDCl_3$) δ = 10.10 (s, 1H), 7.97 (d, J = 8.1 Hz, 2H), 7.80 (d, J = 8.1 Hz, 2H), 7.03 (s, 1H), 3.97 (t, J = 6.4 Hz, 2H), 1.32 (ddd, J = 18.2, 12.0, 5.4 Hz, 10H), 0.89 (t, J = 6.8 Hz, 3H). ^{13}C NMR (400 MHz, $CDCl_3$) δ = 192.01, 150.38, 144.59, 135.04, 130.36, 130.16, 129.42, 115.89, 69.66, 31.73, 29.25, 28.87, 26.00, 22.53, 14.06. HRMS (m/z): $[M]^+$ calcd. for $C_{34}H_{42}O_4$: 514.31; found: 515.3181 $[M + H]^+$.

Synthesis of 2'-(2-(2-Methoxyethoxy)ethoxy)-[1,1':4',1''-terphenyl]-4,4''-dicarbaldehyde (sDEG-CHO)

To a 100 mL round-bottom flask was charged with 2,5-dibromophenol (1.90 g, 11 mmol) and 1-bromo-2-(2-methoxyethoxy)ethane (4.00 g, 22 mmol). 30 mL DMF was added to the flask, and the mixture was stirred vigorously until the monomer was dissolved. Then, K_2CO_3 (3.10 g, 22 mmol) was added, and the system was subsequently stirred at room temperature for 10 h. Subsequently, the reaction mixture was poured into water (400 mL), and extracted with ethyl acetate. The organic layers were collected, washed with water until the water layers became neutral, dried with

$MgSO_4$, filtered, and evaporated by rotary evaporation. The oil crude products were purified by column chromatography (petroleum ether and ethyl acetate as the eluents, respectively) to give the sDEG-Br (yield 89%). sDEG-Br: ^{1}H NMR (400 MHz, $CDCl_3$) δ = 7.40 (d, J = 8.4 Hz, 1H), 7.08 (d, J = 2.1 Hz, 1H), 6.99 (dd, J = 8.4, 2.1 Hz, 1H), 4.22–4.18 (m, 2H), 3.95–3.91 (m, 2H), 3.82–3.77 (m, 2H), 3.62–3.58 (m, 2H), 3.42 (s, 3H).

To a 250 mL round-bottom flask was charged with sTFG-Br (1.77 g, 5 mmol,) and 4-formylphenylboronic acid (1.81 g, 4.53 mmol). After the flask being evacuated and filled with nitrogen, 100 mL THF were added into. Aqueous potassium carbonate solution (50 mL, 13.90 g) and bis(triphenylphosphine)palladium(II) dichloride (400 mg) were successively added in under nitrogen atmosphere. The reaction was stirred at 100 °C for 12 h. After being cooled to room temperature, the THF was evaporated by rotary evaporation, and the residual solution was then extracted with dichloromethane. The organic layers were collected, washed with water until the water layers became neutral, dried with $MgSO_4$, filtered, and evaporated by rotary evaporation. The oil crude products were purified by column chromatography (petroleum ether/ethyl acetate = 10/1 as the eluent) to give sDEG-CHO (yield 72%). sDEG-CHO: ^{1}H NMR (400 MHz, $CDCl_3$) δ = 10.11 (s, 1H), 10.09 (s, 1H), 7.98 (dd, J = 19.8, 8.2 Hz, 4H), 7.83 (dd, J = 9.9, 8.3 Hz, 4H), 7.50 (d, J = 7.9 Hz, 1H), 7.37 (dd, J = 7.9, 1.6 Hz, 1H), 7.29 (d, J = 1.6 Hz, 1H), 4.32–4.27 (m, 2H), 3.87–3.83 (m, 2H),

3.66 (dd, J = 5.6, 3.5 Hz, 2H), 3.55 (dd, J = 5.6, 3.5 Hz, 2H), 3.39 (s, 3H). HRMS (m/z): $[M]^+$ calcd. for $C_{25}H_{24}O_5$: 404.16; found: 405.1706 $[M + H]^+$.

Synthesis of 2'-(Heptyloxy)-[1,1':4',1''-terphenyl]-4,4''-dicarbaldehyde (sHEP-CHO).

To a 100 mL round-bottom flask was charged with 2,5-dibromophenol (1.90 g, 11 mmol) and 1-bromoheptane (3.92 g, 22 mmol). 30 mL DMF was added to the flask, and the mixture was stirred vigorously until the monomer was dissolved. Then, K_2CO_3 (3.10 g, 22 mmol) was added, and the system was subsequently stirred at room temperature for 10 h. Subsequently, the reaction mixture was poured into water (400 mL), and extracted with ethyl acetate. The organic layers were collected, washed with water until the water layers became neutral, dried with $MgSO_4$, filtered, and evaporated by rotary evaporation. The oil crude products were purified by column chromatography (petroleum ether and ethyl acetate as the eluents, respectively) to give the sHEP-Br (yield 78%). sHEP-Br: ^1H NMR (400 MHz, $CDCl_3$) δ = 7.40 (d, J = 8.4 Hz, 1H), 7.02 (d, J = 2.1 Hz, 1H), 6.97 (dd, J = 8.4, 2.1 Hz, 1H), 4.02 (t, J = 6.5 Hz, 2H), 1.94–1.78 (m, 2H), 1.55–1.30 (m, 8H), 0.92 (t, J = 6.8 Hz, 3H).

To a 250 mL round-bottom flask was charged with sHEP-Br (1.75 g, 5 mmol,) and 4-formylphenylboronic acid (1.81 g, 4.53 mmol). After the flask being evacuated and filled with nitrogen, 100 mL THF was added into. Aqueous potassium carbonate solution (50 mL, 13.90 g) and bis(triphenylphosphine)palladium(II) dichloride (400 mg) were successively added in under nitrogen atmosphere. The reaction was stirred

at 100 °C for 12 h. After being cooled to room temperature, the THF was evaporated by rotary evaporation, and the residual solution was then extracted with dichloromethane. The organic layers were collected, washed with water until the water layers became neutral, dried with $MgSO_4$, filtered, and evaporated by rotary evaporation. The oil crude products were purified by column chromatography (petroleum ether/ethyl acetate = 5/1 as the eluent) to give sHEP-CHO (yield 81%). sHEP-CHO: ^1H NMR (400 MHz, $CDCl_3$) δ = 10.11 (s, 1H), 10.09 (s, 1H), 7.99 (dd, J = 18.9, 8.1 Hz, 4H), 7.81 (dd, J = 11.0, 8.2 Hz, 4H), 7.49 (d, J = 7.9 Hz, 1H), 7.34 (d, J = 7.9 Hz, 1H), 7.28 (s, 1H), 4.10 (t, J = 6.4 Hz, 2H), 1.84–1.74 (m, 2H), 1.47–1.22 (m, 8H), 0.90 (t, J = 6.7 Hz, 3H). HRMS (m/z): $[M]^+$ calcd. for $C_{27}H_{28}O_3$: 400.20; found: 401.2125 $[M + H]^+$.

Synthesis of COFs

In a typical procedure, the mixture of TAPP (27.0 mg, 0.04 mmol) and dialdehyde monomers (0.08 mmol), and solvents with aqueous acetic acid (6 M) (ratio details are in Supplementary Tables 2–5) was successively added into a 10 mL Pyrex tube. The mixture was then sonicated for 20 min. The Pyrex tube was degassed through three freeze–pump–thaw cycles. The tube was flame-sealed and heated at 120 °C for 72 h. The precipitate was collected by centrifugation, washed with anhydrous THF and acetone, and then dried under vacuum at 60 °C for 12 h to achieve the relative COF, including DEG-HEP-COF, DEG-COF, HEP-COF, DEG+HEP-COF, sDEG-COF, and sHEP-COF.

Animals.

Sprague Dawley (SD) rats (6−8 weeks, 150 g, female) were obtained from Vital River Laboratory Animal Center (Beijing, China) and raised in a specific pathogen-free environment. All animal studies were approved by the Animal Care and Use Committee of the National Center for Nanoscience and Technology (approval number: NCNST21-2210-0601). All rats were housed with free water and chow according to the guidelines of the National Institutes of Health (16 h light/8 h dark cycle, 22 °C).

Instrumental Characterization.

Powder X-ray diffraction (PXRD) patterns were measured by Xeuss SAXS/WAXS system (Xenocs, France) with Cu Kα radiation (λ = 1.5418 Å). The ^{1}H and ^{13}C NMR spectra were recorded on a Bruker DMX-400 NMR spectrometer (Bruker, Germany) with tetramethylsilane (TMS) as an internal reference. The Fourier transform infrared spectroscopy (FT-IR) spectra were recorded on Perkin-Elmer Spectrum One spectrometer (Perkin-Elmer Instruments Co. Ltd, USA). The mass spectra were recorded by Waters Xevo G2-XS TOF mass spectrometer. Thermogravimetric analyses (TGA) were performed using a Pyris Diamond thermogravimetric/differential thermal analyzer (Perkin-Elmer Instruments Co. Ltd, USA) by heating the samples in temperature range from room temperature to 800 °C under N_2 atmosphere with 10 °C min^{-1} heating rate. Solid-state ^{13}C cross-polarization magic angle spinning (CP/MAS) NMR data were derived from Bruker Advance III 400 spectrometer (Bruker, Germany). Field emission scanning electron microscopy (FE-SEM) images were obtained on a FEI Sirion-200 or Hitachi high technologies (S-8200) field-emission scanning electron microscope at an electric voltage of 5 kV, respectively. X-ray photoelectron spectra (XPS) experiments were carried out on a Thermo ESCALAB 250Xi analyzer (Thermo Fisher Scientific Inc., USA) using 300W Al K α radiation. The Brunauer–Emmett–Teller (BET) specific surface area was calculated based on the nitrogen sorption isotherms measured at 77K using a 2020 volumetric adsorption analyzer (Micromeritics Instrument Corporation, USA)

and pore size distribution profiles were calculated using Original Density Functional Theory model (Model is N_2 at 77 K on carbon (slit pores)). All the porous materials were degassed at 120 °C for 12 h under vacuum before measurement. The molecular weight of newly synthesized compounds was tested by High-resolution mass spectral (HR-MS) (Waters Xevo G2-XS TOF mass spectrometer through an electrospray ionization (ESI) interface).

Scanning Tunneling Microscopy (STM) Characterization.

TAPP and DEG-HEP-CHO were first dissolved in tetrahydrofuran with a concentration of 0.01mg mL^{-1}, and the building blocks were mixed in a volume ratio of about 1:3 (TAPP: DEG-HEP-CHO). Then, 5 μL amount of the mixed solution was deposited on the freshly cleaved surface of highly oriented pyrolytic graphite (HOPG), and then the HOPG was moved into a closed system in the presence of 1.1 g $CuSO_4•5H_2O$ powder at the bottom. After heating at 180 °C for 3 h, the HOPG was cooled down to room temperature, and STM characterization was performed at the octanoic acid/HOPG interface after adding a drop of octanoic acid onto the surface. STM experiments were carried out by using a Multimode scanning tunneling microscope (Bruker, Germany) at room temperature under ambient air condition. STM tips were mechanically cut Pt/Ir wires (90:10). All the STM images presented in the paper were recorded by using the constant current mode.

Calculation.

Structure models were generated using the Material Studios 2017/R2 modeling (Accelrys Inc.). Geometry optimization was performed using the Forcite module. Full profile pattern fitting (Rietveld) was performed against the experimental powder pattern using the Reflex module.

The structural optimization and quantitative calculation of thermodynamics were performed under the Density Functional Theory (DFT) method by Gaussian 16: ES64L-G16RevA.03 (Gaussian, Inc.). These compounds were optimized on the theoretical level of B3LYP/6-31+G (d, p). When calculating the thermodynamic energy, we used the basis set and method of B3LYP/def2tzvp to calculate the molecular energy. The electrostatic potential of different organic compounds was calculated by the wave function of different configurations. The wave function was analyzed by Multiwfn. To compare different geometries, density-functional tight-binding method (DFTB+) was employed to optimize the structure and calculate total energies of various 2D COF layers. Our calculations were carried out using the DFTB+ program, using Slater–Koster library in which O-N-H-C parameters were based on an early publication. Specifically, the Coulombic interaction between partial atomic charges was determined under the scheme of the self-consistent charge (SCC) formalism. During the optimization, the SCC tolerance is 10^{-5} kcal mol^{-1}, and the convergence tolerance was set as 0.01 kcal mol^{-1} for total energy and 0.2 kcal mol^{-1} $Å^{-1}$ for force. The relative total

energy (RTE) is calculated. Two specific DEG-HEP-COF structures have been investigated with periodic boundary conditions (PBC). The 2D lattice was fixed with a = 32.35 Å, b = 47.60 Å, together with a large vacuum layer (> 50 Å). Large vacuum layer was employed to minimize the inter-layer self-interaction, which is critical for the calculation of stacking energy. The whole system for double layers is $C_{320}H_{352}N_{12}O_{24}$ (708 atoms). Layer distance was optimized through the scanning in the range of 3–9 Å, followed by full relaxation at the minimum. For the stacking module, 4 types of stacking have been investigated, including perfectly eclipsed AA, eclipsed AA-derived structures with slight slipping by 0.72 and 1.44 Å in a and b directions as well as alternated AA stacking. Based on full optimizations, the stacking energy (SE) was calculated. Optimum monolayer was obtained through comparing the total energy of different geometries, with their relative stabilities being labelled by relative crystal stacking energy (RSE). All energies are in unit of kcal mol^{-1}.

$$RTE = E_{\mathrm{A}} - E_{\mathrm{F}}$$

$$SE = E_{\mathrm{D}} - 2 \times E_{\mathrm{S}}$$

$$RSE = E_{\mathrm{SA}} - E_{\mathrm{SF}}$$

E_{A}: Energy of any configure; E_{F}: Energy of the most favourable configure; E_{D}: Energy of stacked double layers; E_{S}: Energy of stacked single layer; E_{SA}: Stacking energy of any configure; E_{SF}: Stacking energy of the most favourable configure.

Antibacterial property and wound healing testing

Antibacterial tests of COFs.

The antibacterial effects of DEG-HEP-COF, DEG-COF, and HEP-COF were tested by turbidity assays. We selected MRSA as microbial model. We diluted three types of COFs to 100 μg mL^{-1} with the Luria–Bertani (LB) liquid medium inoculated with the MRSA. The MRSA concentration was 10^5 colony-forming units (CFU/mL). The mixtures were incubated at 37 °C for 24 h with/without visible light irradiation. After 24 h, we observed the turbidity of the bacterial suspension and recorded the images.

Hemolysis of DEG-HEP-COF.

The DEG-HEP-COF hemolysis ability was tested using the fresh blood from rats. The blood samples were centrifuged at 200 g for 15 min and washed with saline for three times to get erythrocytes. The obtained erythrocytes were re-diluted into 4% suspension. The 4% suspensions mixed with DEG-HEP-COF at different concentrations were incubated at 37 °C for 3 h. Water was used as a positive control and saline was used as a negative control in this experiment. The supernatants were acquired through centrifugation at 9600 g for 15 min and then measured the absorbance at 540 nm by UV-vis spectrophotometer (Lambda-950, PerkinElmer, USA).

Preparation and characterization of DEG-HEP-COF@Antibiotics.

Synthesis of DEG-HEP-COF@Lev. Lev (0.5–2%, w/v) was dissolved into 5 mL of acetic acid, and the mixture was stirred for 10 min. Then, 5mg of DEG-HEP-COF

was added into the above solution with a continuous stirring at 20 g for 24 h. The particles (DEG-HEP-COF@Lev) were collected by centrifugal separation (9600 g 15 min), and washed three times using deionized water before drying at 50 °C.

Synthesis of DEG-HEP-COF@Van. Van (0.5–2%, w/v) was dissolved into 5 mL of water, and the mixture was stirred for 10 min. Then, 5 mg of DEG-HEP-COF was added into the above solution with a continuous stirring at 20 g for 24 h. The particles (DEG-HEP-COF@Van) were collected by centrifugal separation (9600 g, 15 min), and washed three times using ethanol before drying at 50 °C.

Synthesis of DEG-HEP-COF@Lev&Van. DEG-HEP-COF@Van (0.5–2%, w/v) was added into the solution with 5 mg of Lev and 5 mL of acetic acid with a continuous stirring again at 20 g for 24 h. The obtained particles (DEG-HEP-COF@Lev&Van) were collected by centrifugal separation (9600 g, 15 min), and washed three times using deionized water before drying at 50 °C.

FT-IR measurement of DEG-HEP-COF@Antibiotics. FT-IR spectra of DEG-HEP-COF@Antibiotics were acquired by a FT-IR spectrometer (Spectrum One, PerkinElmer Instruments, USA) in the range of 4000–500 cm^{-1} in a KBr matrix.

Loading capability of Lev and Van. The total amounts of Lev and Van effectively loaded within DEG-HEP-COF@Antibiotics were determined by absorbance measurements of the remaining solutions at 292 and 280 nm using UV-vis spectrophotometer (Lambda-950, PerkinElmer, USA) according to the standard curve,

respectively. The antibiotic loading capability (LC) of DEG-HEP-COF@Antibiotics was calculated according to the following equation:

$$LC = \frac{W_{\mathrm{L}}}{W_{\mathrm{T}}} \times 100\%$$

W_{L}: Weight of loaded antibiotics; W_{T}: total weight of DEG-HEP-COF@Antibiotics.

Release of Lev and Van.

The released amount of Lev and Van from DEG-HEP-COF@Antibiotics were determined by the UV-vis absorbance. We dispersed 5 mg of DEG-HEP-COF@Antibiotics into 10 mL of phosphate buffered saline (PBS) solution (15% ethanol) with shaking at 37 °C. At predetermined time intervals, 200 μL of solution was removed for release profile testing and replaced with an equal amount of PBS.

Cytocompatibility and biocompatibility of DEG-HEP-COF@Antibiotics.

The human umbilical vein endothelial cells (HUVECs) and mouse-derived fibroblasts (L929) were selected to estimate the cytocompatibility of DEG-HEP-COF@Antibiotics. We cultivated HUVECs and L929 in dulbecco's modified Eagle medium (DMEM) with the addition of 10% fetal bovine serum (FBS) and 1% penicillin/streptomycin. The cells were cultured in a 96-well plate at a density of 10^5 cells/well overnight. We incubated cells with different DEG-HEP-COF@Antibiotics at same concentration (100 μg mL^{-1}) for 24 h. We washed the cells with PBS three times and stained the cells with a cell counting kit-8 (CCK-8) for 2 h of incubation. We measured the optical density at 450 nm of the cells in each well by reading from a microplate reader (EnSpire multimode reader, PE, USA).

Preparation and characterization of DEG-HEP-COF@Antibiotics membranes.

PCL pellets and gelatin powder in a ratio of 10/3 (w/w) were dissolved in hexafluoroisopropanol (HFIP) at 10 wt %, stirring for 6 h followed by the addition of COF@Antibiotics (final concentration: 100 μg mL^{-1}), and then the mixture was stirred for another 6 h. We employed electrostatic spinning equipment (ET-2535, Beijing Yongkang Leye Technology Development Company, China) to obtain the nanofibrous films under a positive voltage of 15 kV and negative voltage of –3 kV. We used a 5 mL syringe with a flattened metal needle (no. 22G, with an inside diameter of 0.4 cm) to load the solution. The distance between the receiving plate and needle was 15 cm. The injection rate of the solution was 0.5 mm/min. We collected the nanofibrous films on an aluminum foil adhered onto the grounded receiving plate until the solution was used up. We used a camera (D90, Nikon, Japan) to record the appearance of electrospinning solution and nanofibrous films. We observed the distribution of DEG-HEP-COF@Antibiotics in nanofibrous films by SEM. We tested the hydrophilic properties of nanofiber films by water contact angle analyzer.

Antibacterial tests of DEG-HEP-COF@Antibiotics membranes.

We tested the antibacterial effects of DEG-HEP-COF@Antibiotics membranes soaked into LB liquid medium by turbidity assays. We cut the membranes into circular patches with a diameter of 1.5 cm and put them into 3 mL of LB liquid medium, which contained 10^5 CFU/mL MRSA. The liquid medium was incubated at

37 °C for 24 h. We tested the bacterial suspensions every 6 h at OD600 nm using a microplate reader.

The size of the inhibition zone can reflect the antibacterial effect of the membranes. We cut the membranes into circular patches with a diameter of 1.5 cm. We put them into an agar plate (with a diameter of 9 cm) on which 50 μL of 10^7 CFU/mL MRSA were evenly spread. The agar plate was incubated at 37 °C for 24 h. Then, we measured the sizes of the inhibition zones to estimate the antibacterial activity of the different membranes.

Rat skin model infected with MRSA.

We established a full-thickness wound model of MRSA infection to study the therapeutic effect of the PG-DEG-HEP-COF@antibiotics membranes. We cut one round full-thickness skin wound of 0.5 cm in diameter at the dorsal skin of each rat. The wounds were infected with 100 μL of MRSA (10^7 CFU/mL) for 0.5 h. The infected rats were randomly divided into 5 groups (n = 3). We put the PG, PG-DEG-HEP-COF, PG-DEG-HEP-COF@Lev, PG-DEG-HEP-COF@Van, and PG-DEG-HEP-COF@Lev&Van membranes on the wounds.

We observed and measured the wound-bed closure of the rats at different periods (0, 4, 8 and 12 d) and euthanized the rats to obtain the wound tissues on Day 12. The wound tissues were collected to perform hematoxylin and eosin (H&E) and Masson staining. The exudate was collected from the infected wound and diluted 100 times

with PBS. Then, 50 μL of the solution was coated on agar plates. The colony counts of MRSA were recorded.

Supplementary Figures

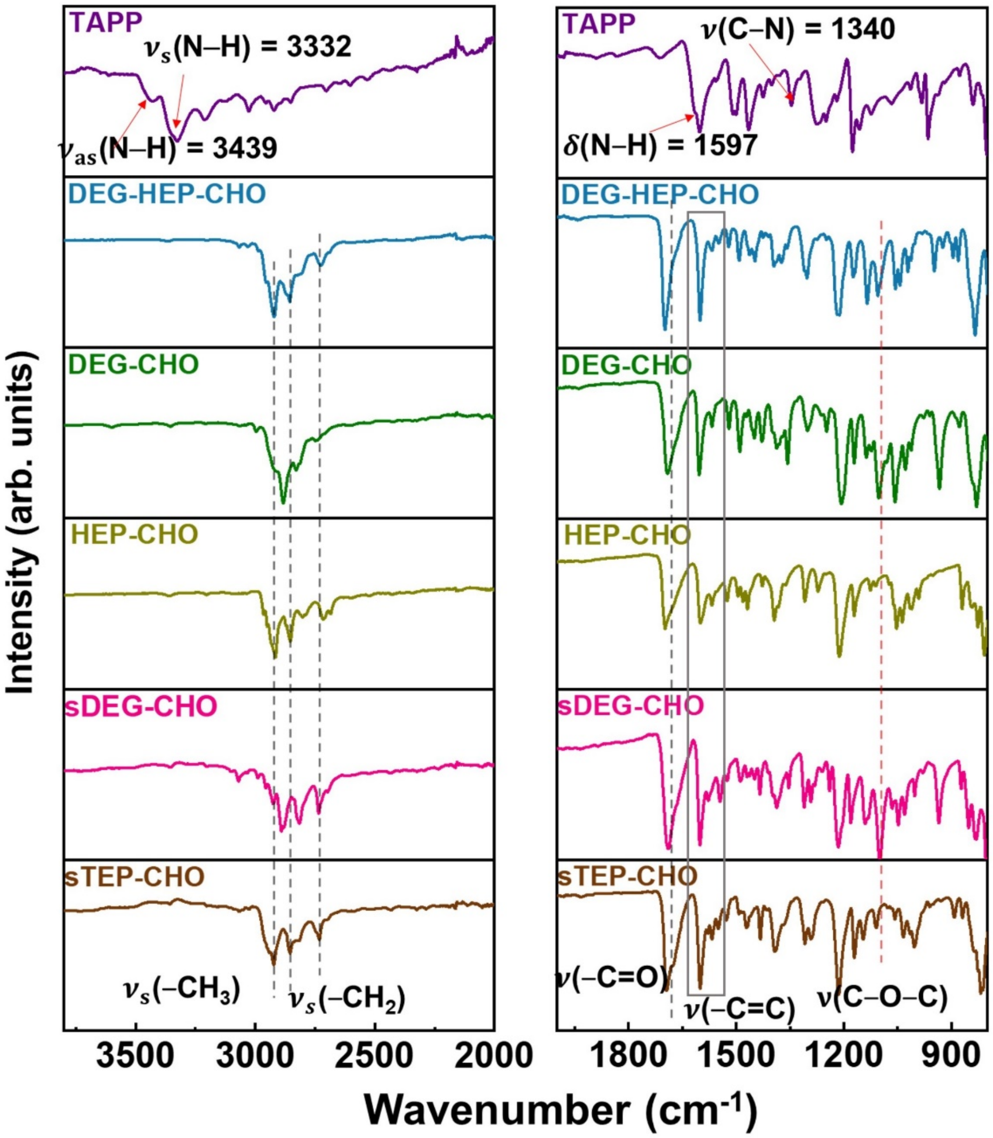

Supplementary Fig. 1. FT-IR spectra. FT-IR spectra of synthesized monomers in this study. Source data are provided as a Source Data file.

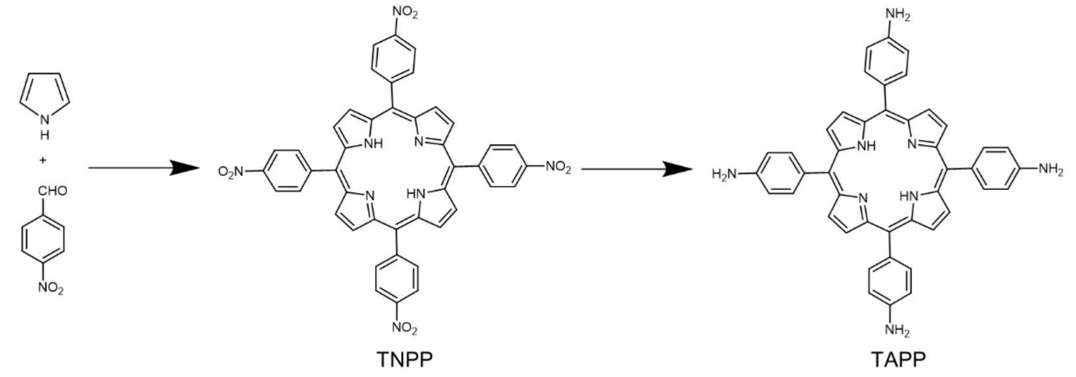

Supplementary Fig. 2. Synthesis scheme. Synthesis route to TAPP.

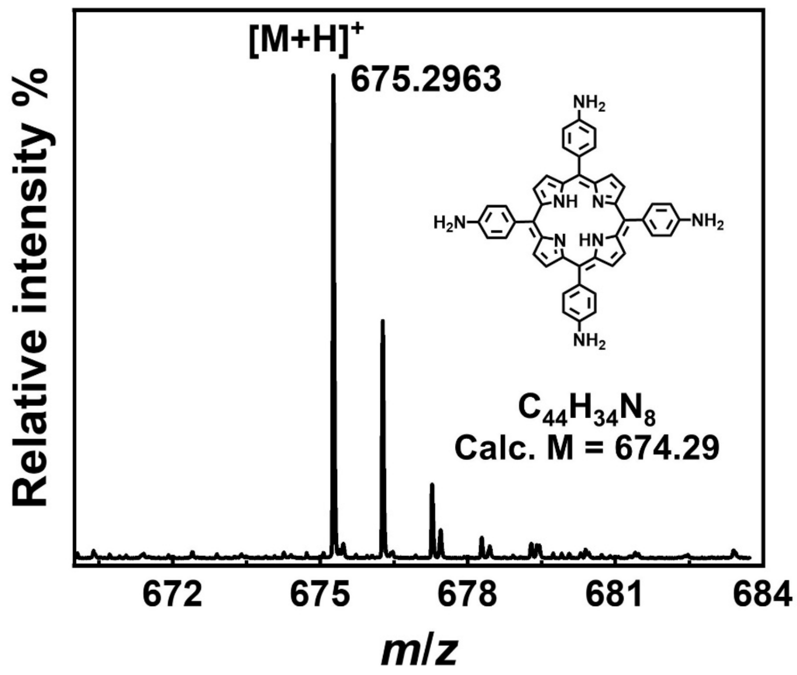

Supplementary Fig. 3. HR-MS spectrum. HR-MS spectrum of TAPP.

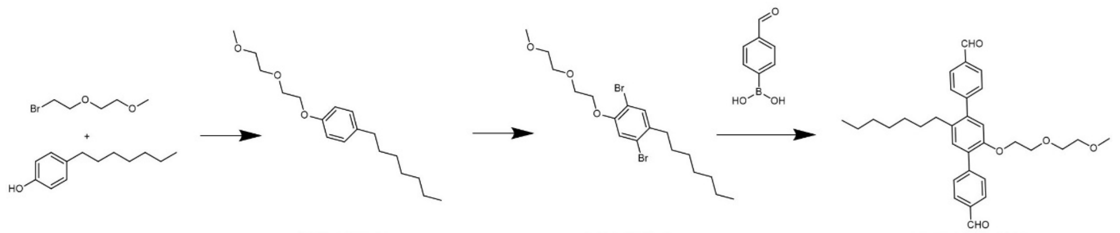

Supplementary Fig. 4. Synthesis scheme. Synthesis route to DEG-HEP-CHO.

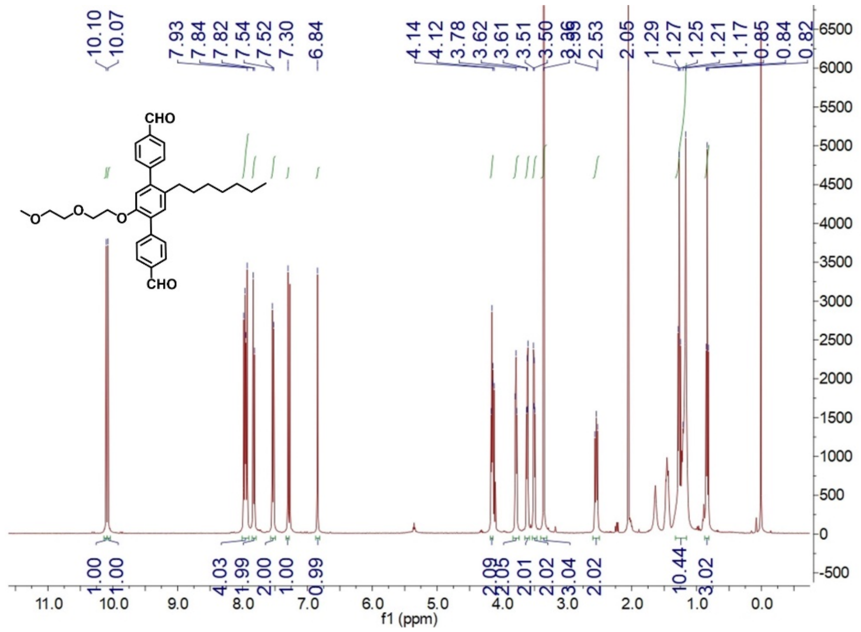

Supplementary Fig. 5. NMR spectrum. ^{1}H NMR spectrum of DEG-HEP-CHO.

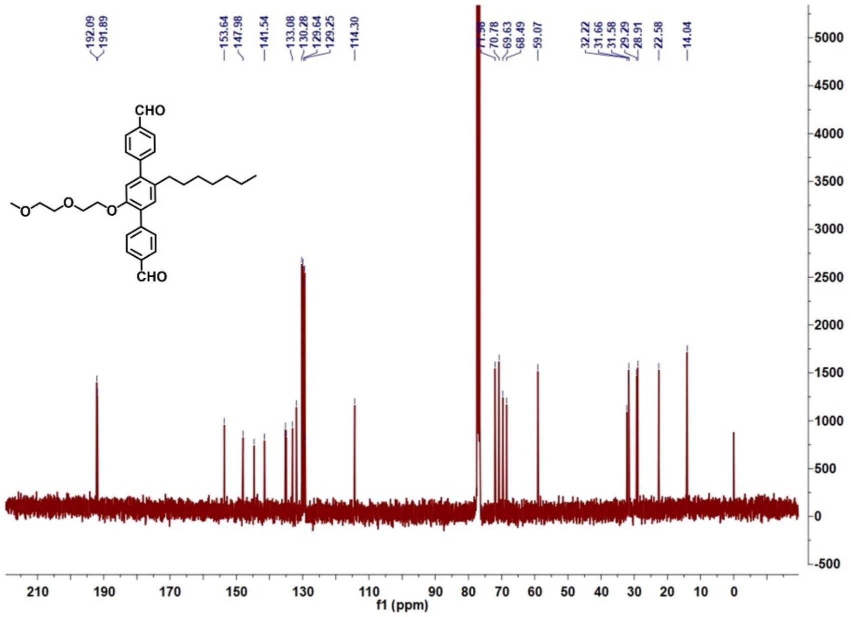

Supplementary Fig. 6. NMR spectrum. ^{13}C NMR spectrum of DEG-HEP-CHO.

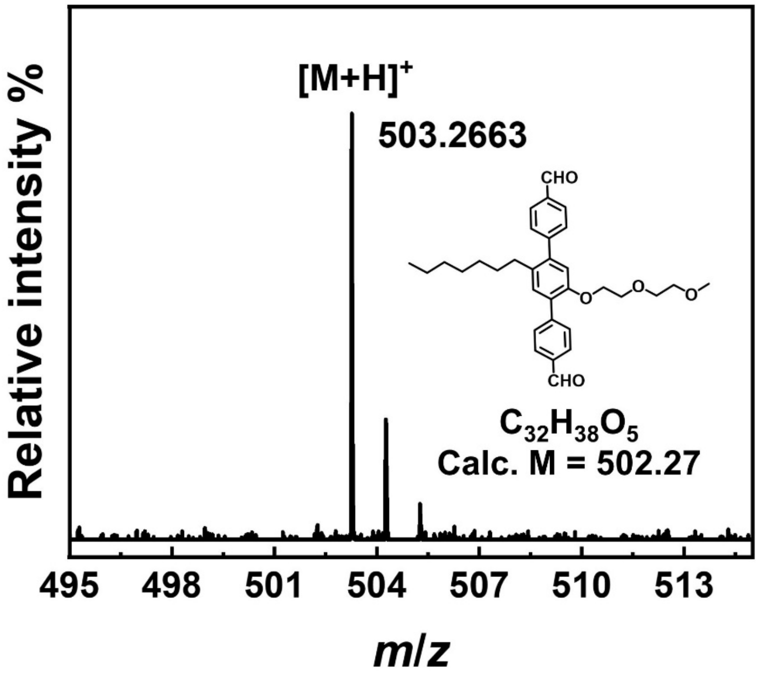

Supplementary Fig. 7. HR-MS spectrum. HR-MS spectrum of DEG-HEP-CHO.

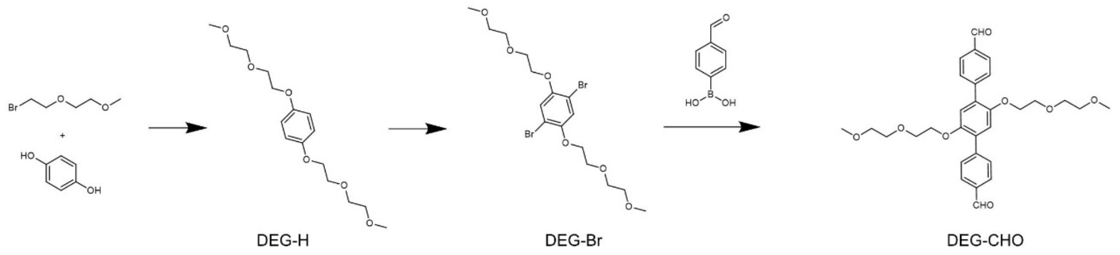

Supplementary Fig. 8. Synthesis scheme. Synthesis route to DEG-CHO.

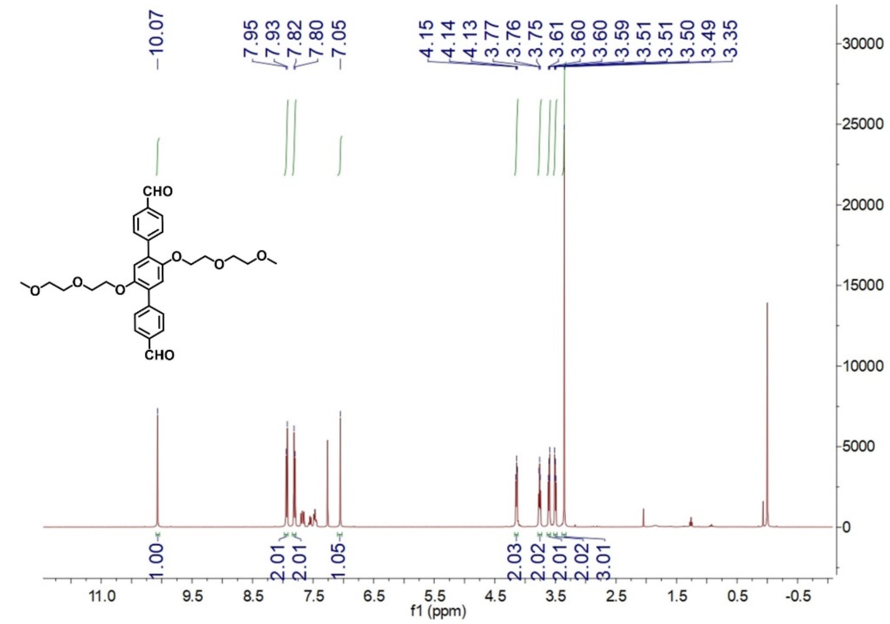

Supplementary Fig. 9. NMR spectrum. ^{1}H NMR spectrum of DEG-CHO.

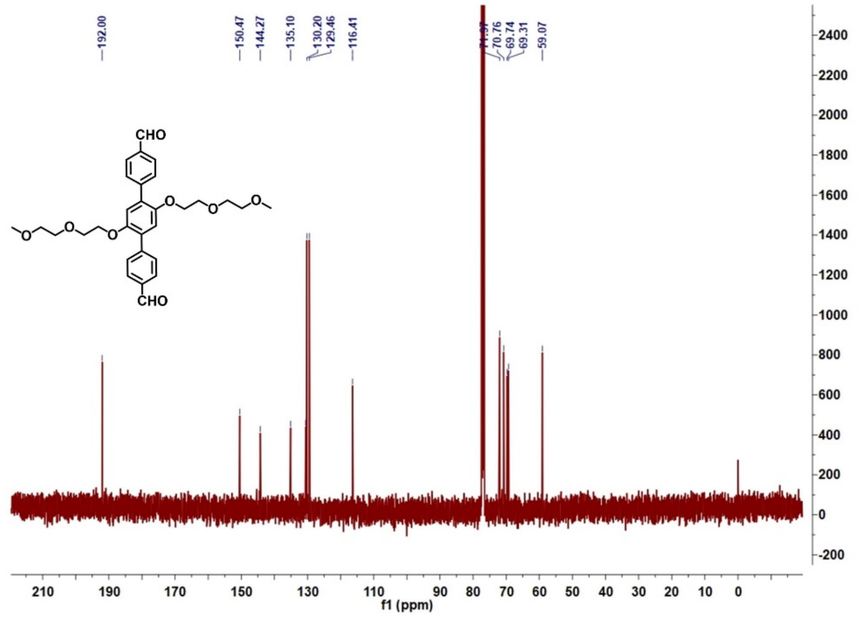

Supplementary Fig. 10. NMR spectrum. ^{13}C NMR spectrum of DEG-CHO.

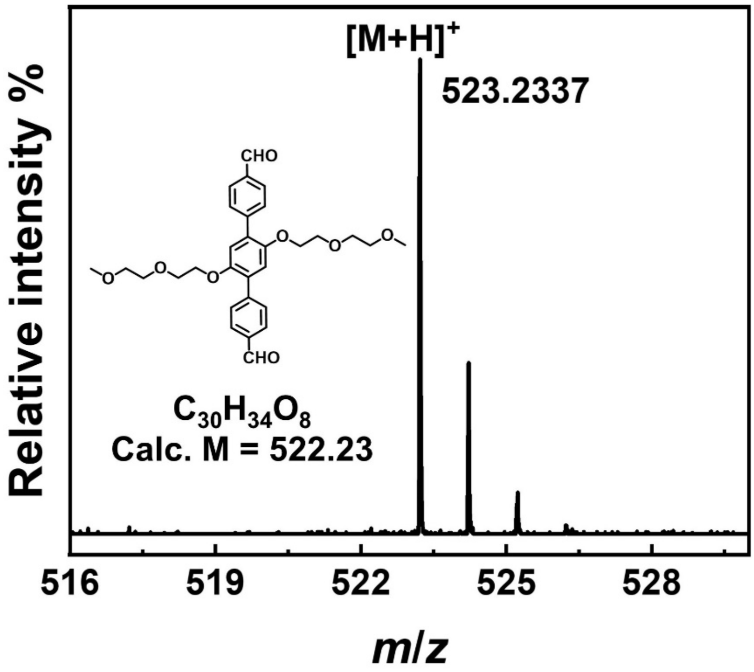

Supplementary Fig. 11. HR-MS spectrum. HR-MS spectrum of DEG-CHO.

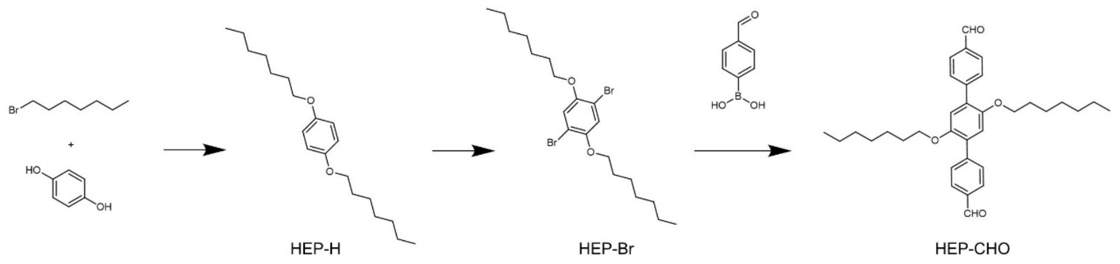

Supplementary Fig. 12. Synthesis scheme. Synthesis route to HEP-CHO.

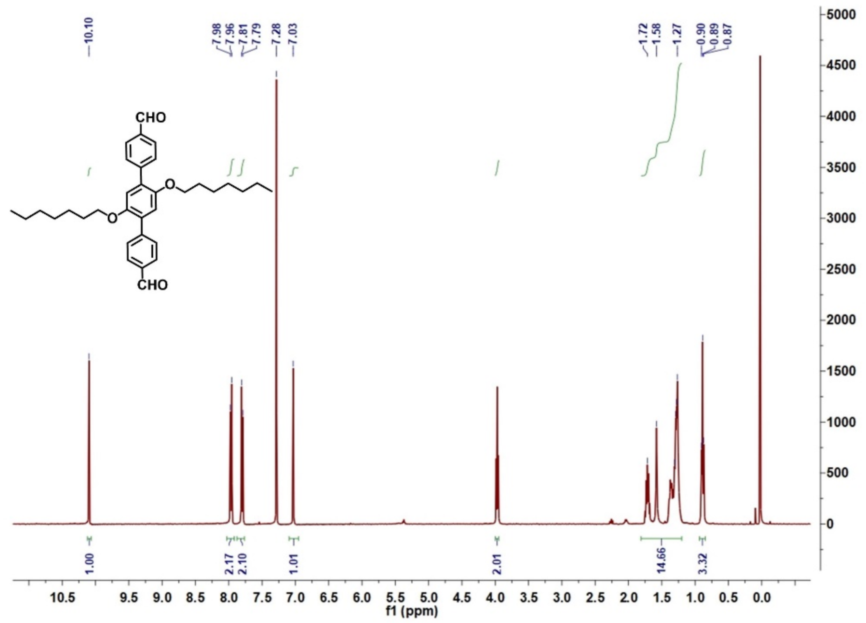

Supplementary Fig. 13. NMR spectrum. ^{1}H NMR spectrum of HEP-CHO.

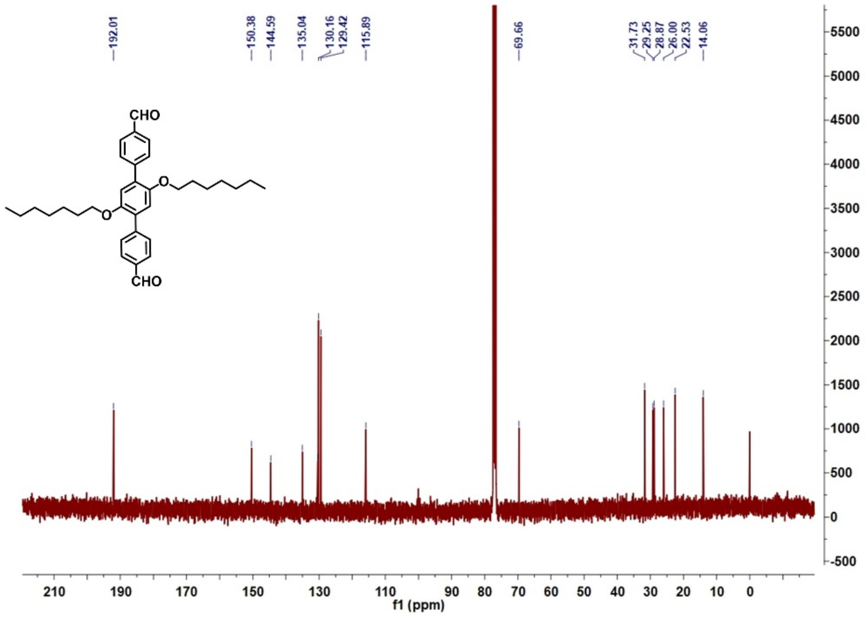

Supplementary Fig. 14. NMR spectrum. ^{13}C NMR spectrum of HEP-CHO.

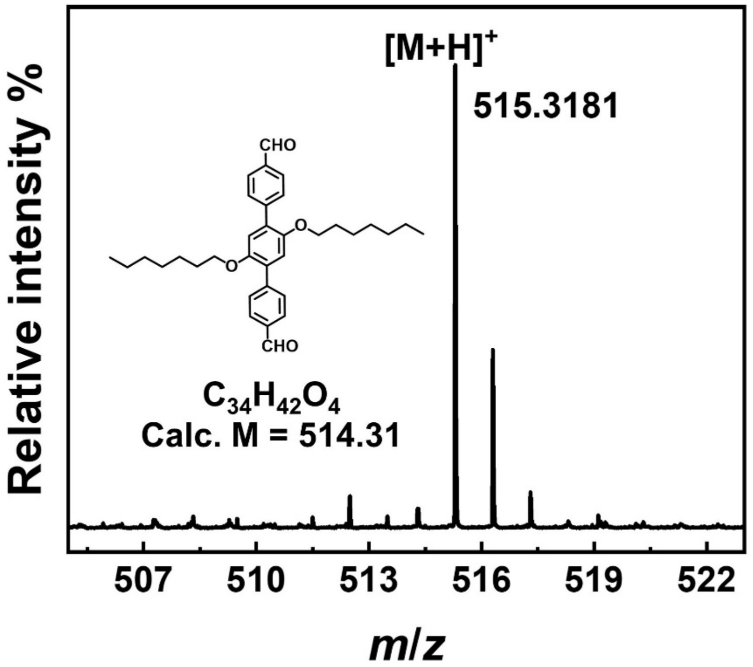

Supplementary Fig. 15. HR-MS spectrum. HR-MS spectrum of HEP-CHO.

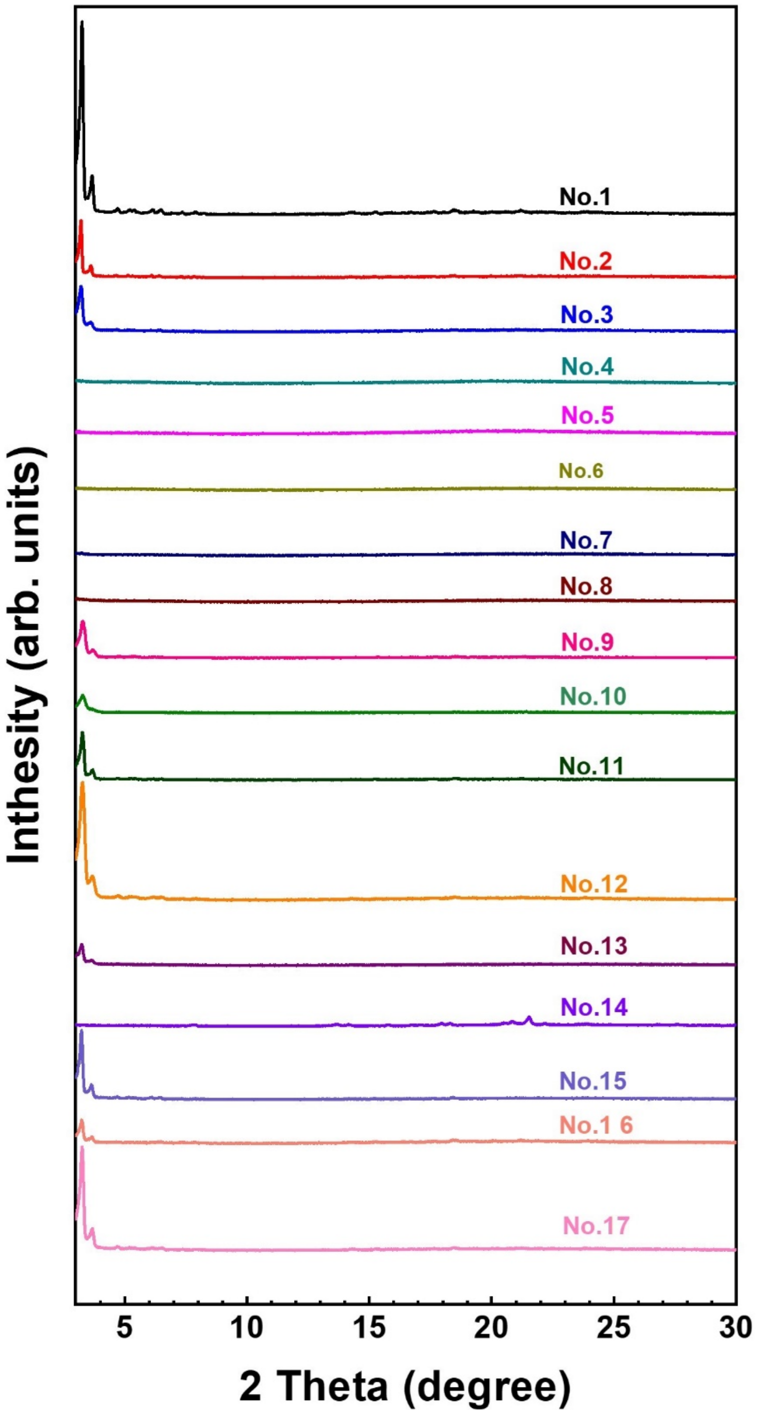

Supplementary Fig. 16. PXRD patterns. PXRD patterns of DEG-HEP-COF obtained under the synthesis conditions listed in Supplementary Table 2. Source data are provided as a Source Data file.

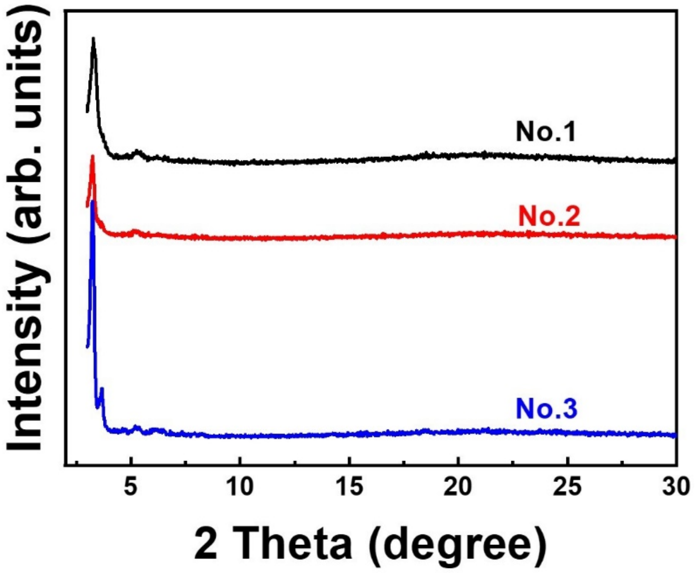

Supplementary Fig. 17. PXRD patterns. PXRD patterns of DEG-COF obtained under the synthesis conditions listed in Supplementary Table 3. Source data are provided as a Source Data file.

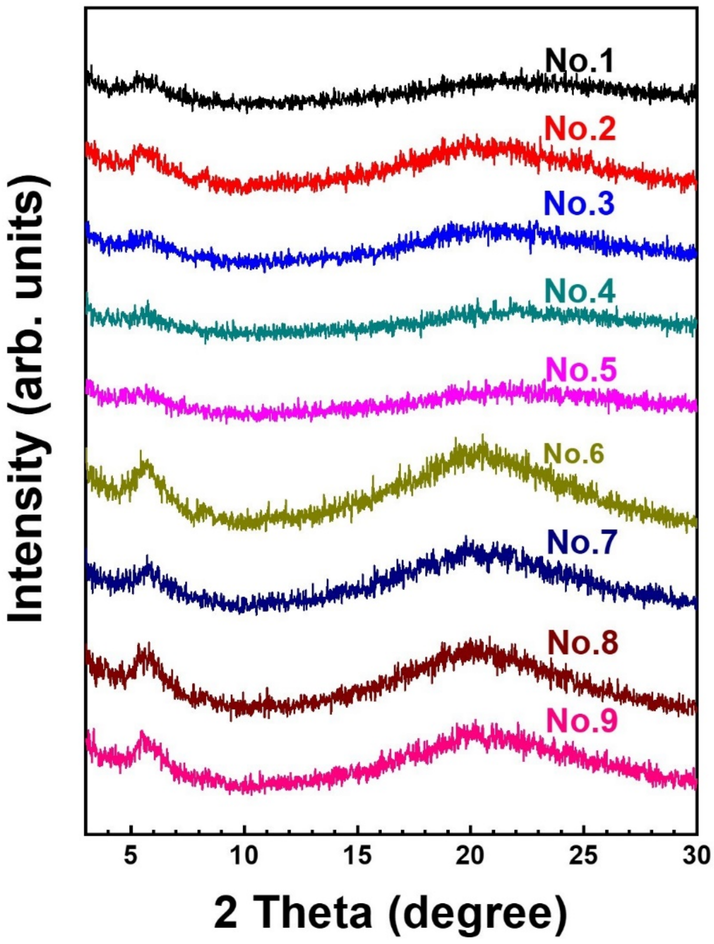

Supplementary Fig. 18. PXRD patterns. PXRD patterns of HEP-COF obtained under the synthesis conditions listed in Supplementary Table 4. Source data are provided as a Source Data file.

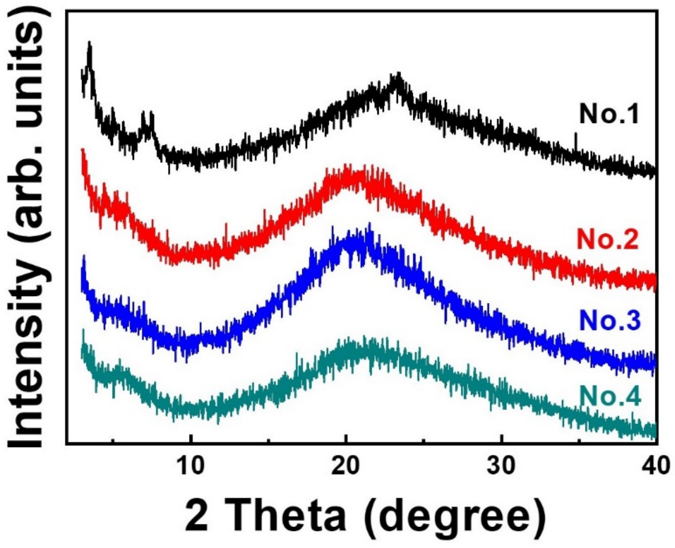

Supplementary Fig. 19. PXRD patterns. PXRD pattern of DEG+HEP-COF obtained under the synthesis condition listed in Supplementary Table 5. Source data are provided as a Source Data file.

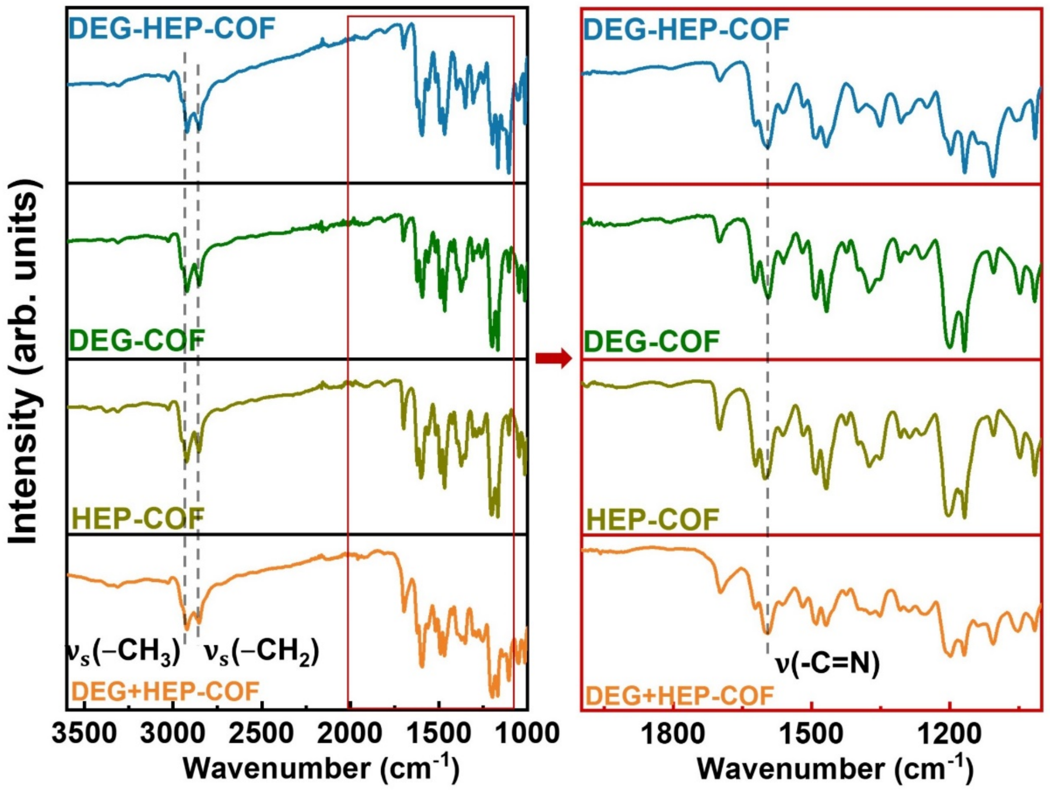

Supplementary Fig. 20. FT-IR spectra. FT-IR spectra of DEG-HEP-COF, DEG-COF, HEP-COF, and DEG+HEP-COF. Source data are provided as a Source Data file.

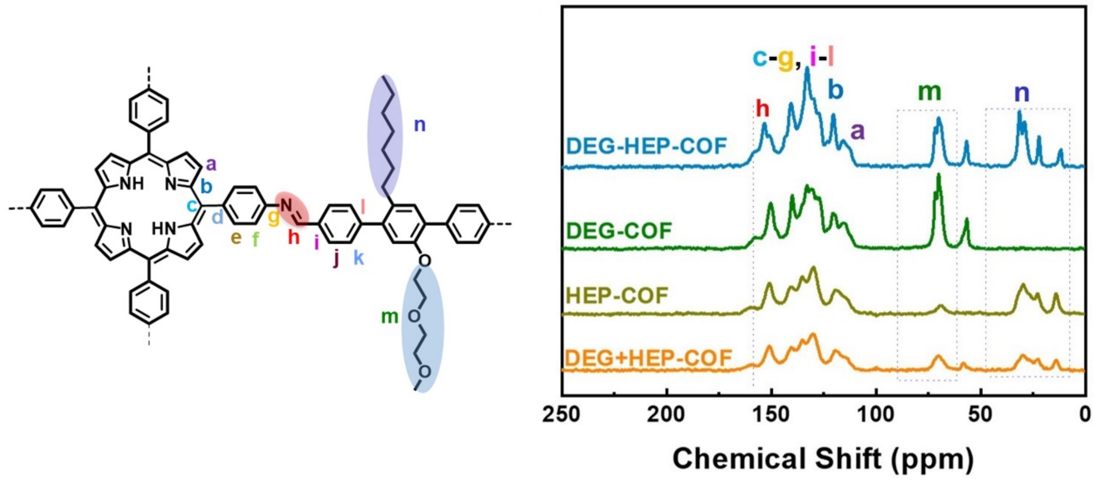

Supplementary Fig. 21. ^{13}C CP/MS NMR spectra. ^{13}C CP/MS NMR spectra of DEG-HEP-COF, DEG-COF, HEP-COF, and DEG+HEP-COF, respectively. The markers in the spectra correspond to markers in the structure. Source data are provided as a Source Data file.

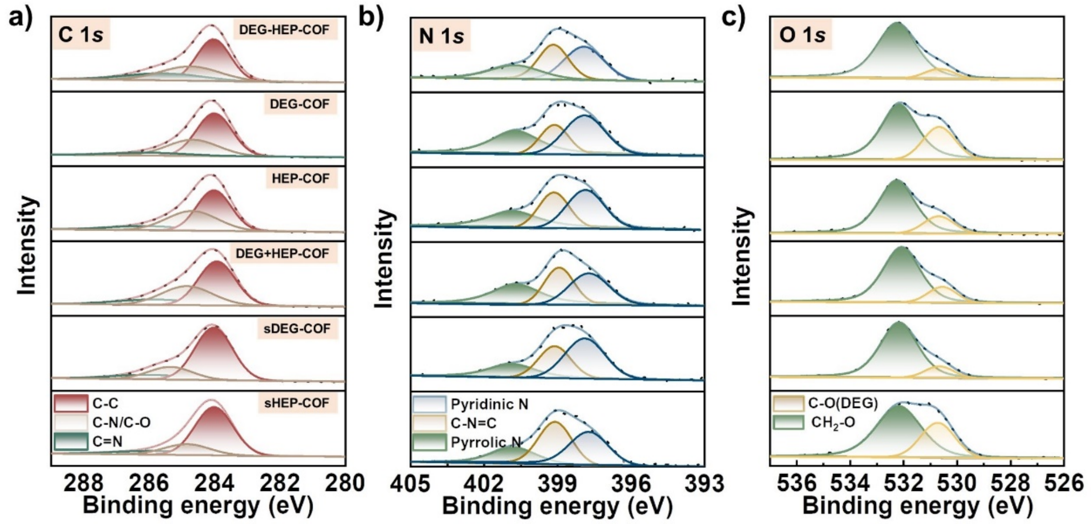

Supplementary Fig. 22. XPS spectra. XPS investigation of synthesized COFs in this study. High-resolution XPS spectra of C 1*s* (a), N 1*s* (b), and O 1*s* (c) for COFs. Source data are provided as a Source Data file.

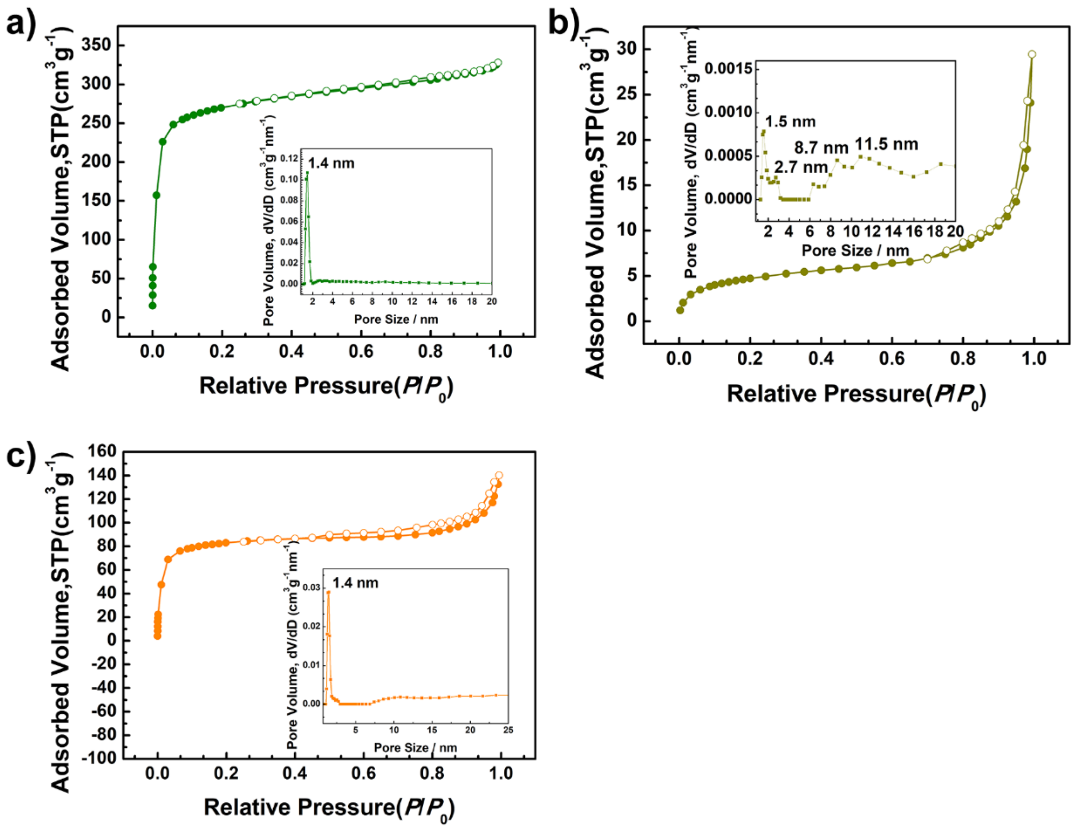

Supplementary Fig. 23. Porous properties. N_2 adsorption and desorption isotherms and pore size distribution profiles (inset) of (a) DEG-COF, (b) HEP-COF, and (C) DEG+HEP-COF. Source data are provided as a Source Data file.

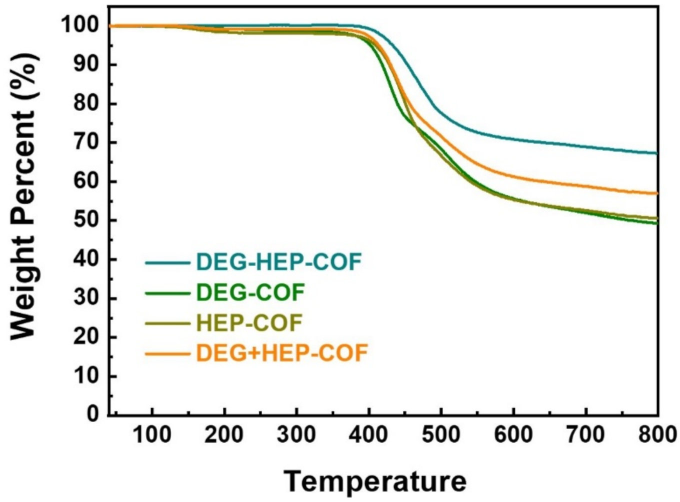

Supplementary Fig. 24. Thermal stability. Thermogravimetric analysis curves of DEG-HEP-COF, DEG-COF, HEP-COF, and DEG+HEP-COF. Source data are provided as a Source Data file.

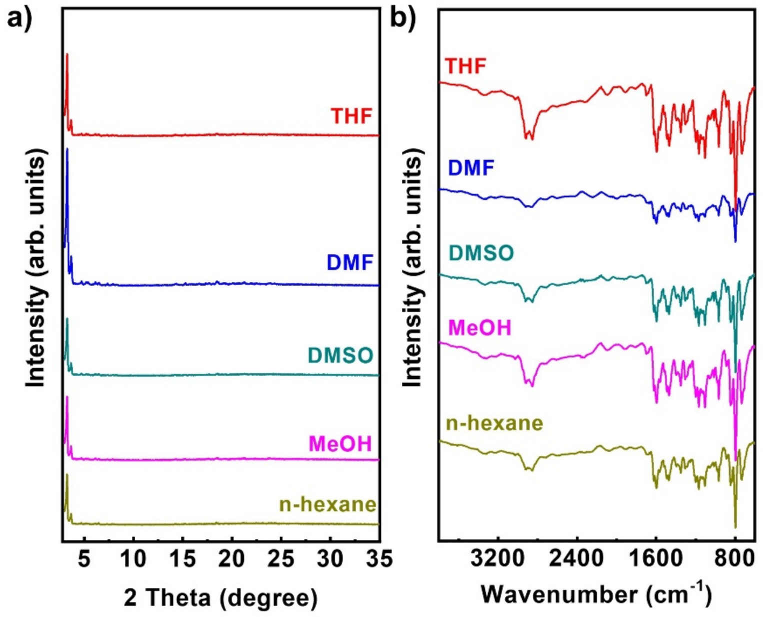

Supplementary Fig. 25. Chemical stability. (a) PXRD patterns and (b) FT-IR spectra of DEG-HEP-COF after chemical treatments. Source data are provided as a Source Data file.

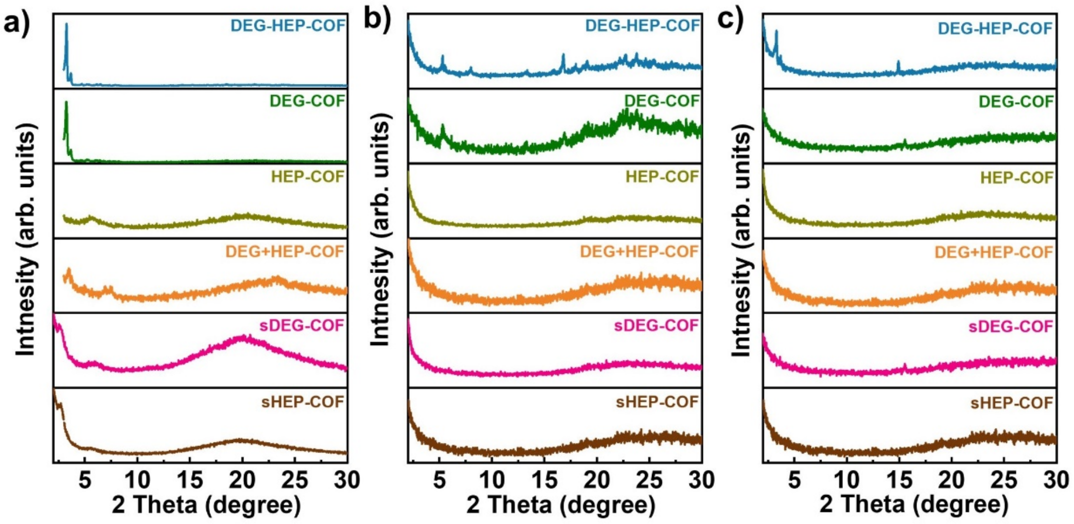

Supplementary Fig. 26. Chemical stability. PXRD patterns of all COFs materials before (a), and after treatments in aqueous 1 M HCl (b) and 1 M NaOH (c) solutions for 1 day. Source data are provided as a Source Data file.

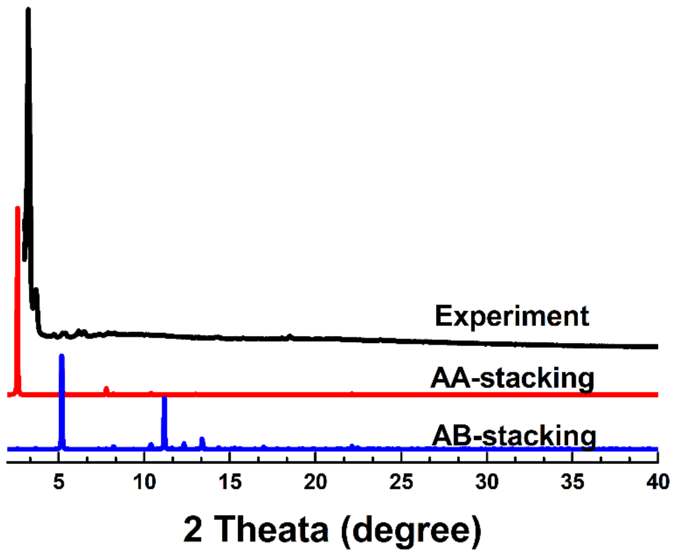

Supplementary Fig. 27. Structure simulation. PXRD profiles of DEG-HEP-COF of the experimentally observed (black) and simulated data using the eclipsed AA (red) and staggered AB (blue) stacking modes. Source data are provided as a Source Data file.

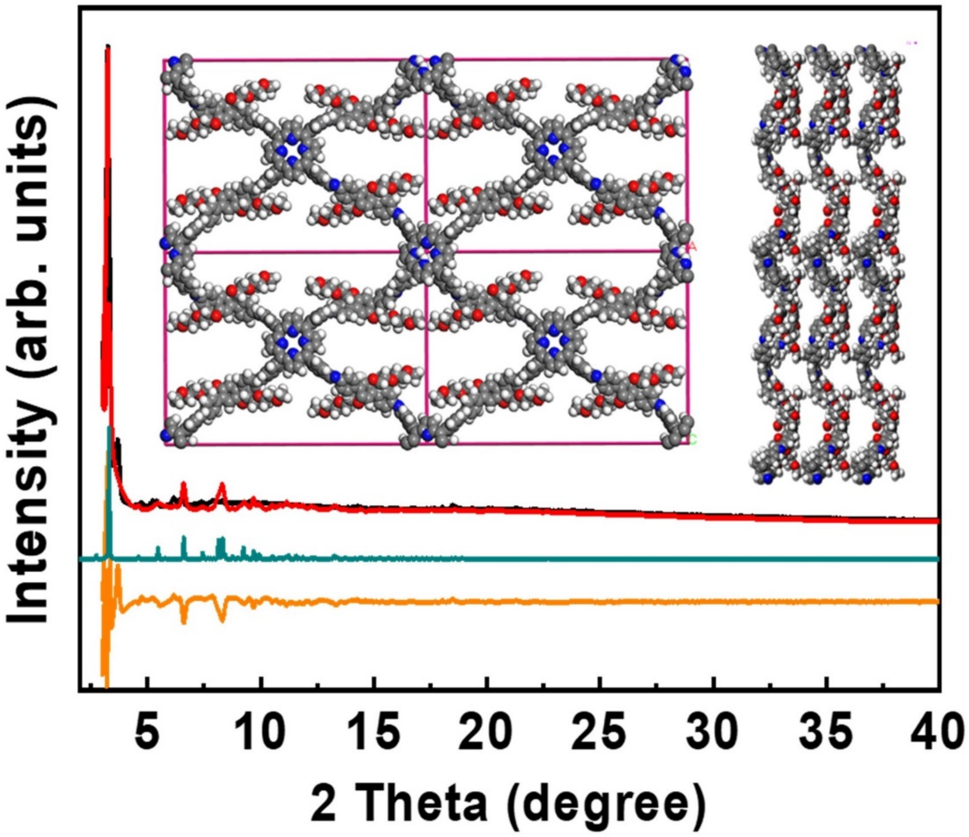

Supplementary Fig. 28. Structure simulation. PXRD profiles of DEG-COF of the experimentally observed (red), Pawley refined (black) and their difference (orange), and simulated (cyan) using the contorted AA stacking mode. The inset is the side and top view of DEG-COF (gray: C, blue: N, red: O white: H). Source data are provided as a Source Data file.

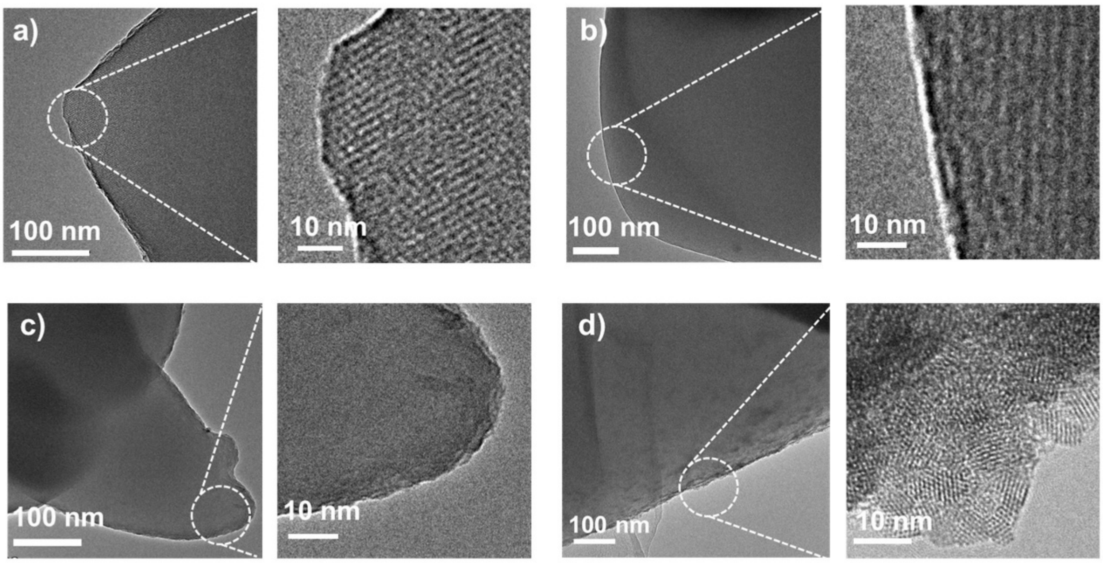

Supplementary Fig. 29. **HR-TEM.** HR-TEM images of DEG-HEP-COF (a), DEG-COF (b), HEP-COF (c), and DEG+HEP-COF (d).

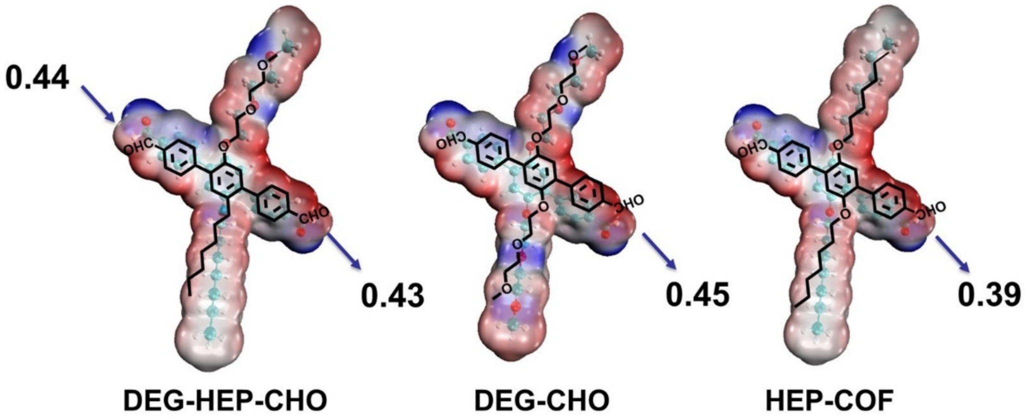

Supplementary Fig. 30. Electrostatic potentials. The electrostatic potentials of DEG-HEP-CHO, DEG-CHO, and HEP-CHO calculated by B3LYP/6-31+g(d,p).

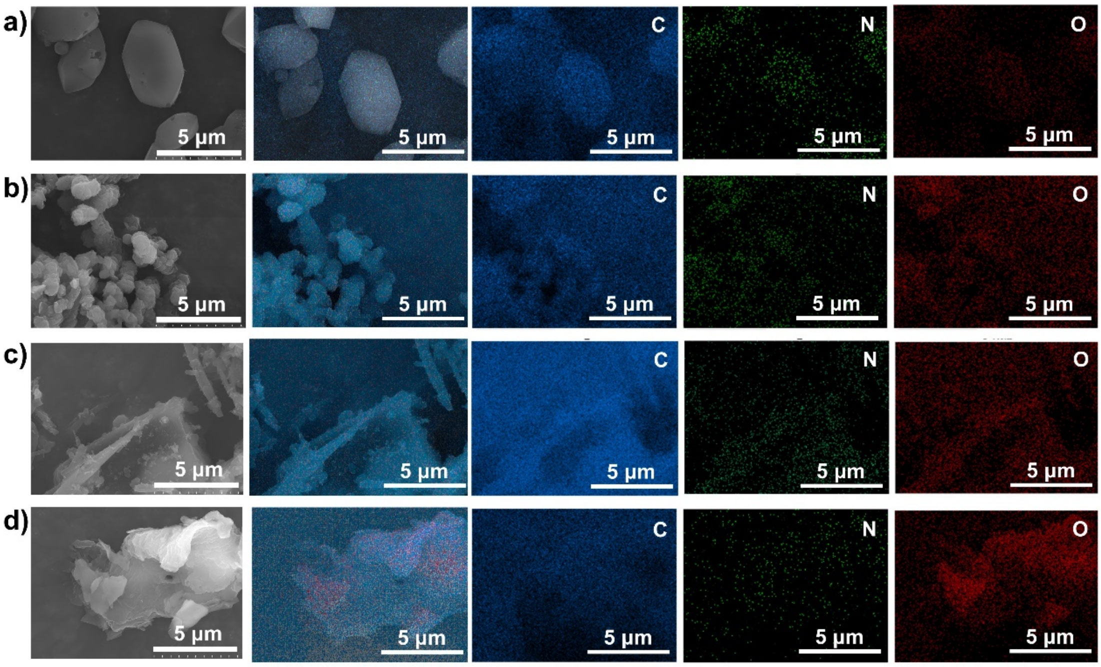

Supplementary Fig. 31. SEM and SEM-EDS. SEM and SEM-EDS images of DEG-HEP-COF (a), DEG-COF (b), HEP-COF (c), and DEG+HEP-COF (d).

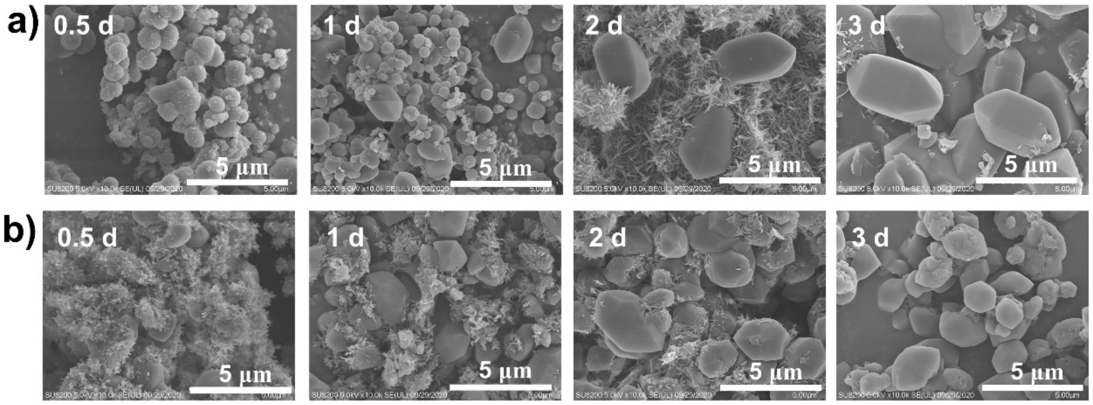

Supplementary Fig. 32. Growth process analysis. SEM images of (a) DEG-HEP-COF and (b) DEG-COF recorded at different reaction time.

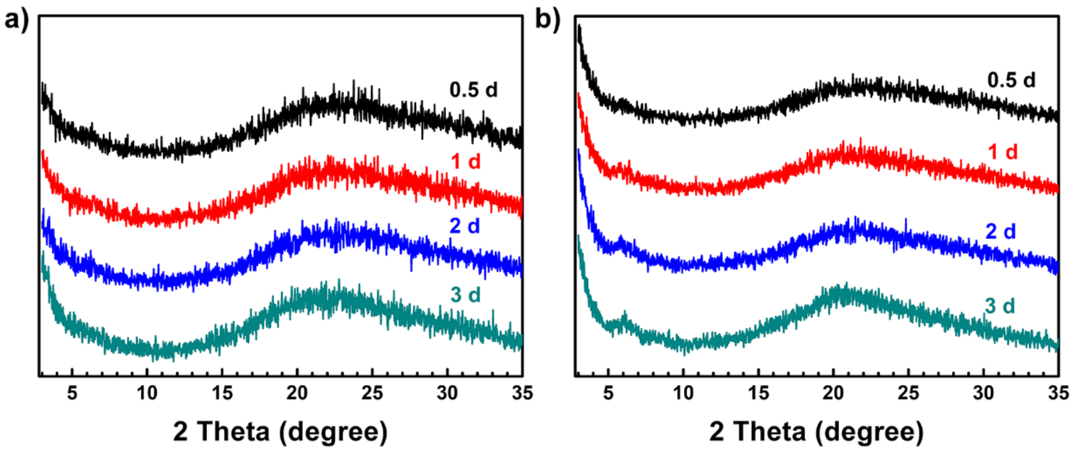

Supplementary Fig. 33. Growth process analysis. PXRD patterns of (a) HEP-COF, and (b) DEG+HEP-COF, respectively, recorded at different intervals of reaction time. Source data are provided as a Source Data file.

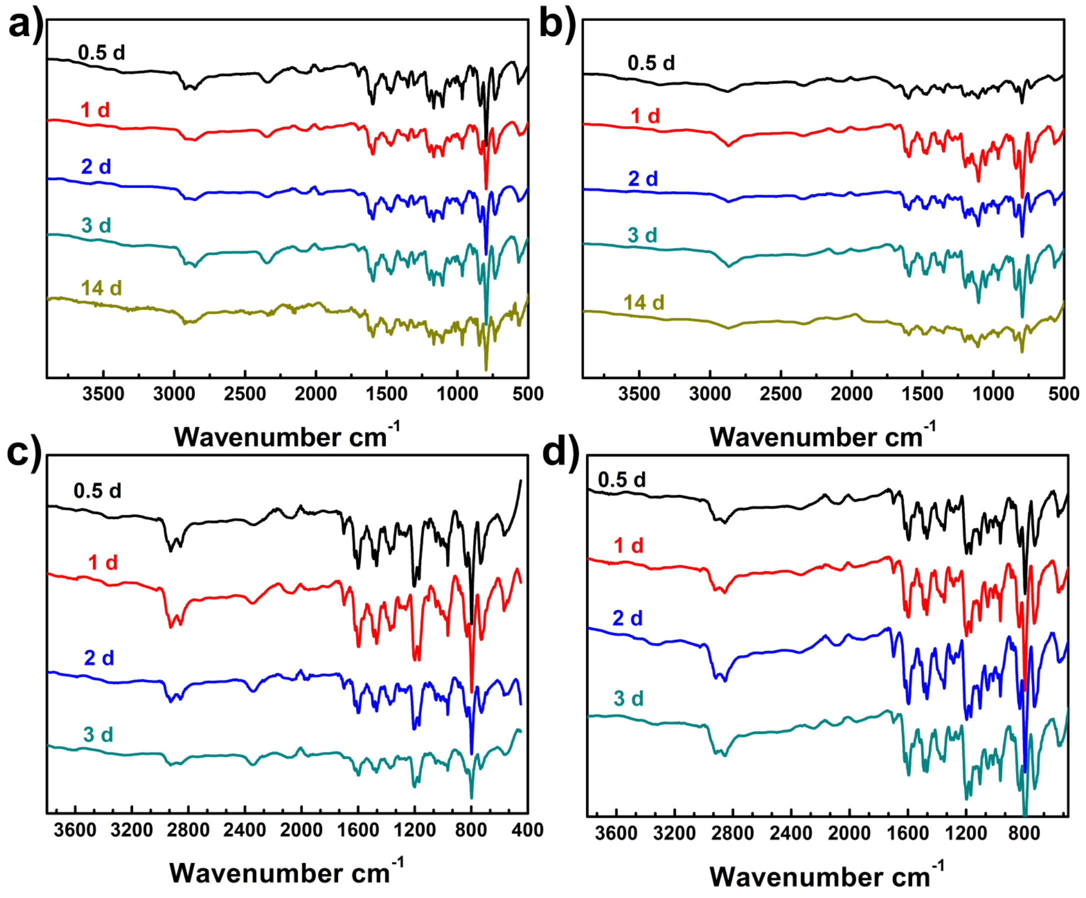

Supplementary Fig. 34. Growth process analysis. FT-IR spectra of (a) DEG-HEP-COF, (b) DEG-COF, (c) HEP-COF, and (d) DEG+HEP-COF, respectively, recorded at different intervals of reaction time. Source data are provided as a Source Data file.

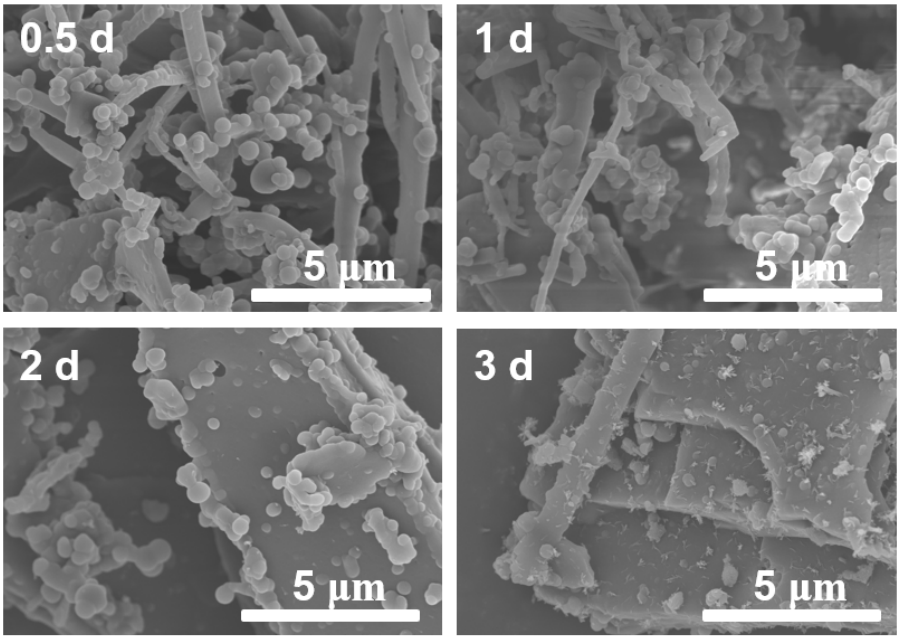

Supplementary Fig. 35. Growth process analysis. SEM images of HEP-COF recorded at different intervals of reaction time.

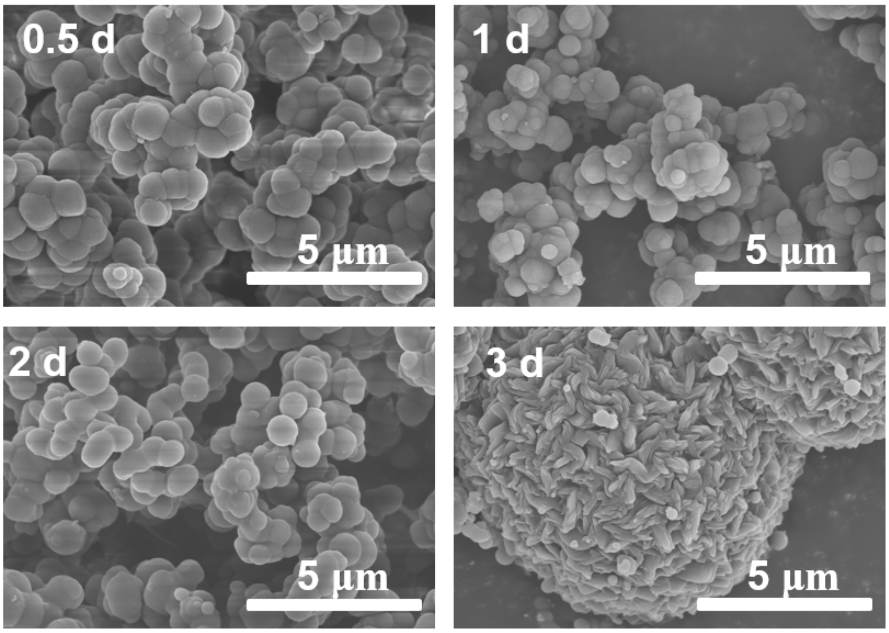

Supplementary Fig. 36. Growth process analysis. SEM images of DEG+HEP-COF recorded at different intervals of reaction time.

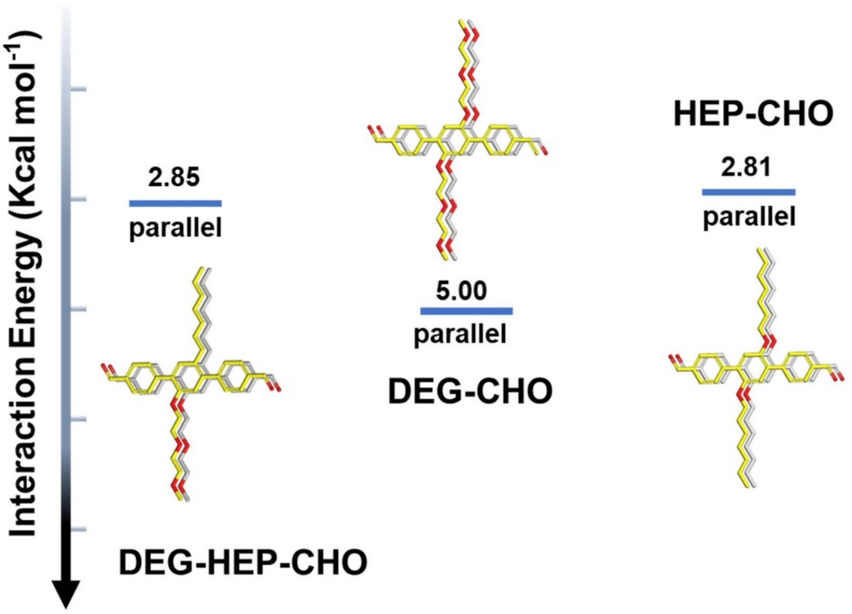

Supplementary Fig. 37. Interlayer interaction. The dimer interaction energies of DEG-HEP-CHO, DEG-CHO, and HEP-CHO calculated by B3LYP/def2tzvp.

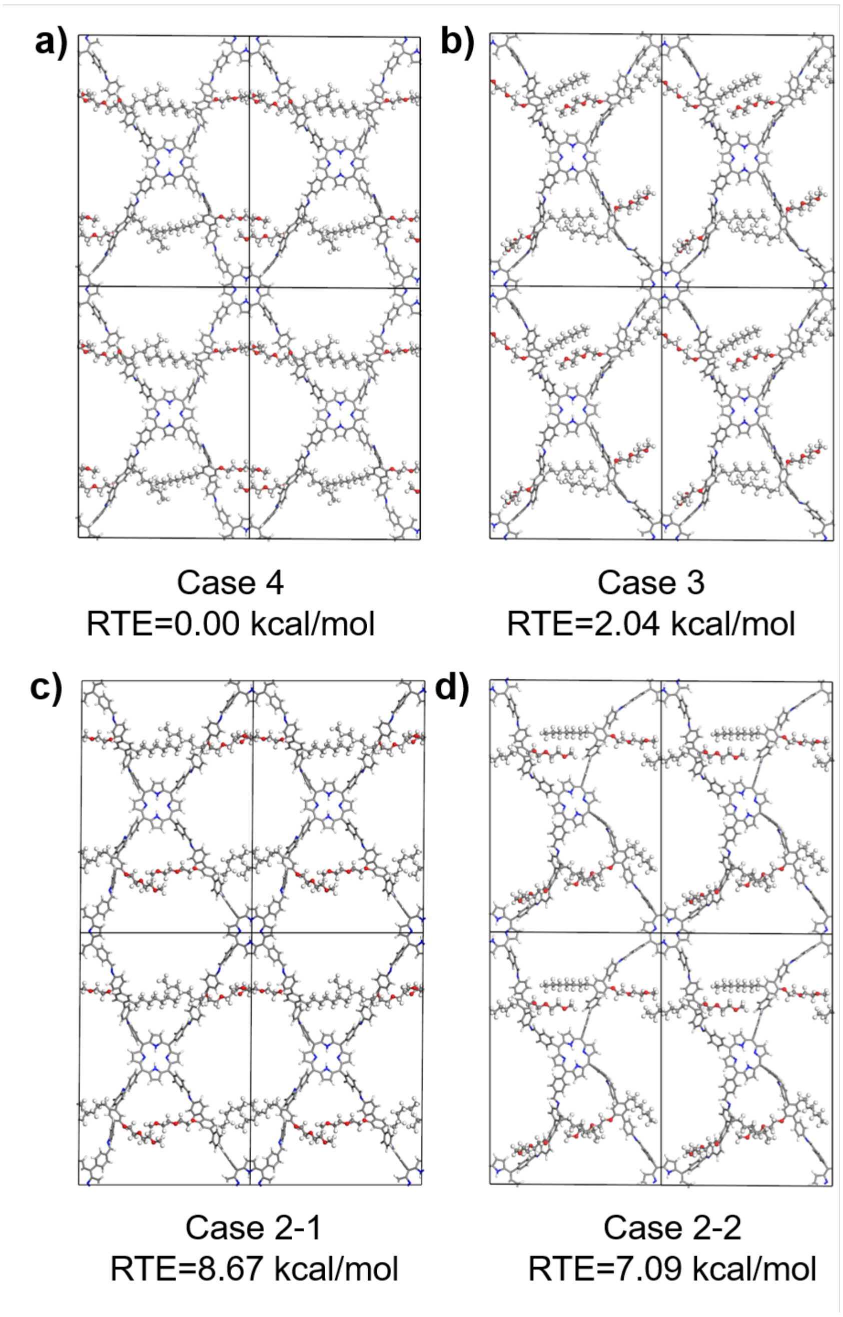

Supplementary Fig. 38. Pore structure verification. The high-resolution image of Fig. 3e. The RTE of tetragons with different chain orientations. (a) Case-4 shows four alkyl chains in the same hole. (b) Case-3 shows three alkyl chains in the same hole. Case-2-1 (c), and Case-2-2 (d) show two alkyl chains in the same hole, which are in ortho-position and in para-position, respectively (gray: C, blue: N, red: O, white: H).

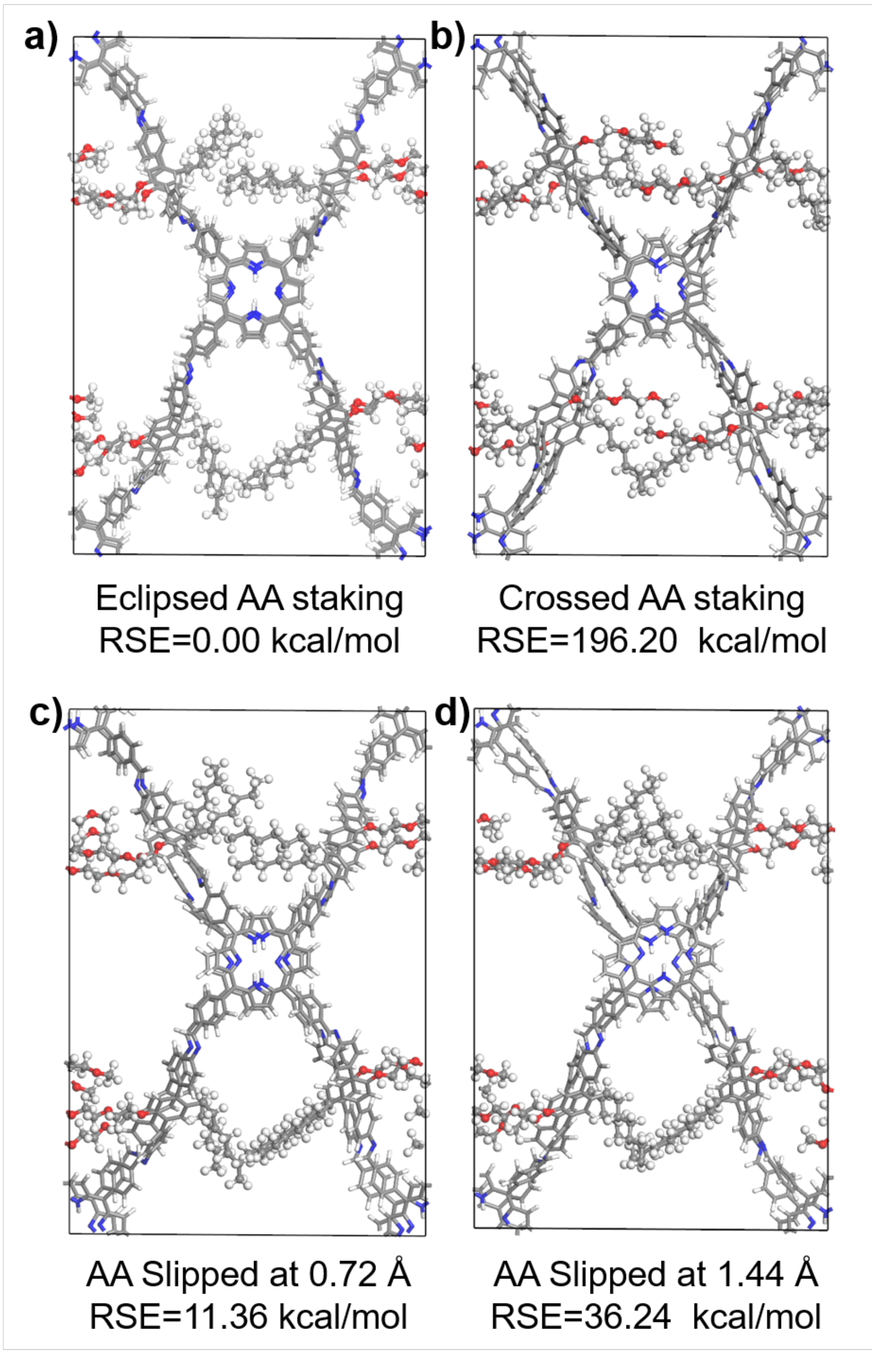

Supplementary Fig. 39. Stacking structure verification. The RSE calculation of eclipsed AA stacking (a), crossed AA stacking (b), AA slipped at 0.72 Å (c), and AA slipped at 1.44 Å (d) modes, respectively (gray: C, blue: N, red: O, white: H). Source data are provided as a Source Data file.

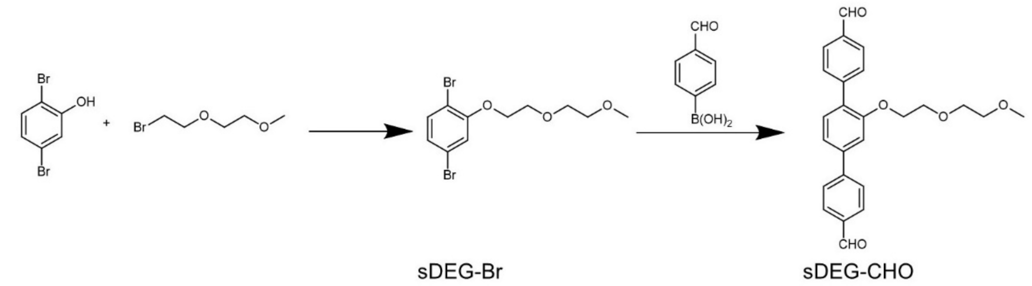

Supplementary Fig. 40. Synthesis scheme. Synthesis route to sDEG-CHO.

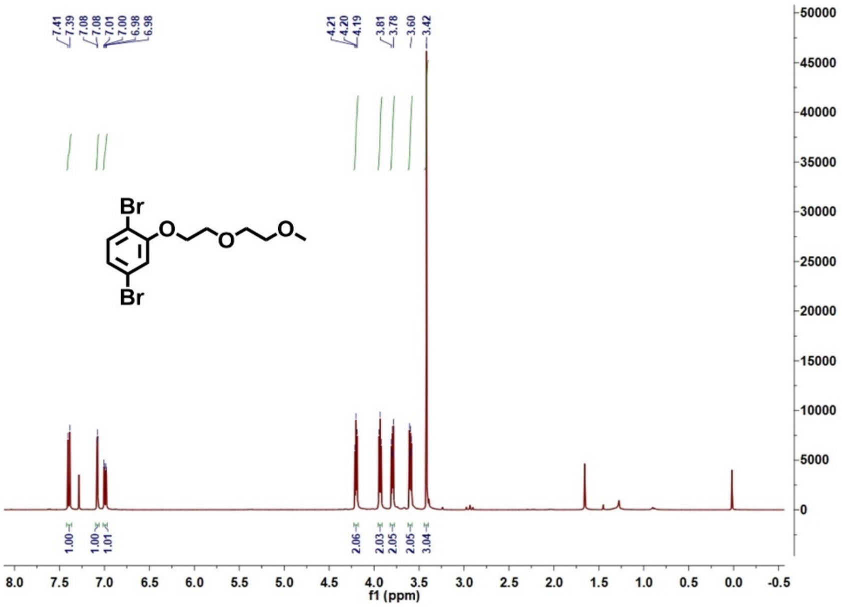

Supplementary Fig. 41. NMR spectrum. ^{1}H NMR spectrum of sDEG-Br.

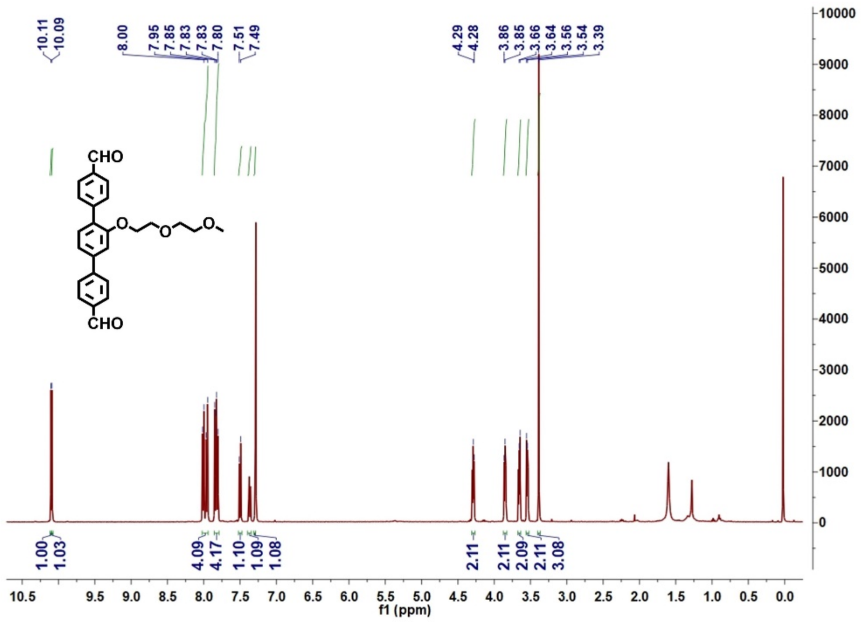

Supplementary Fig. 42. NMR spectrum. ^{1}H NMR spectrum of sDEG-CHO.

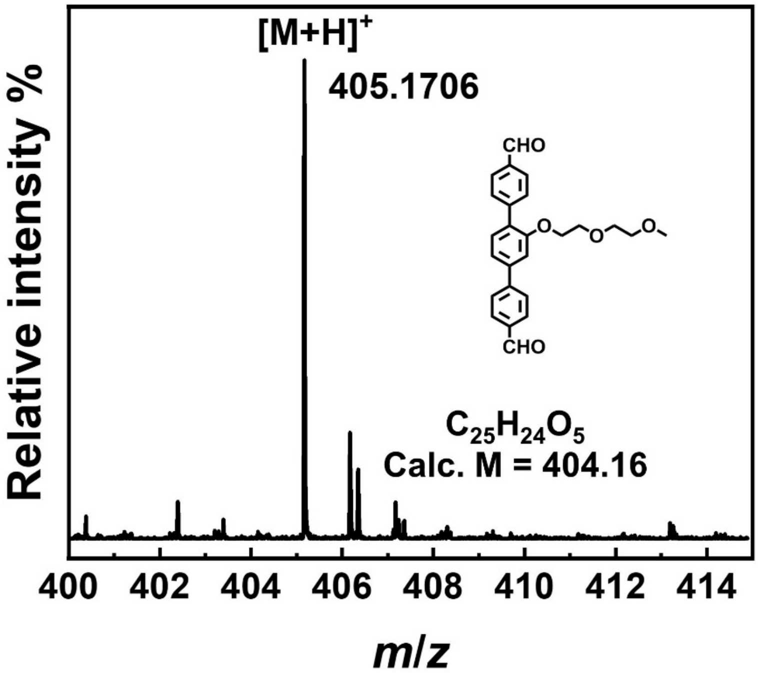

Supplementary Fig. 43. HR-MS spectrum. HR-MS spectrum of sDEG-CHO.

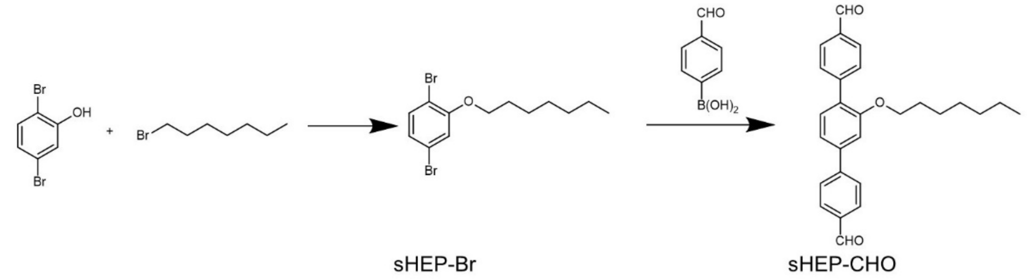

Supplementary Fig. 44. Synthesis scheme. Synthesis route to sHEP-CHO.

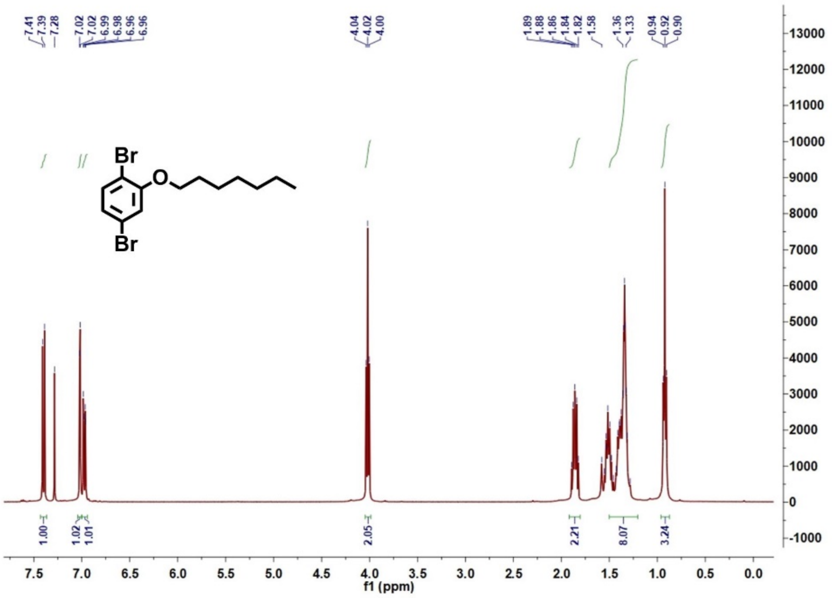

Supplementary Fig. 45. NMR spectrum. ^{1}H NMR spectrum of sHEP-Br.

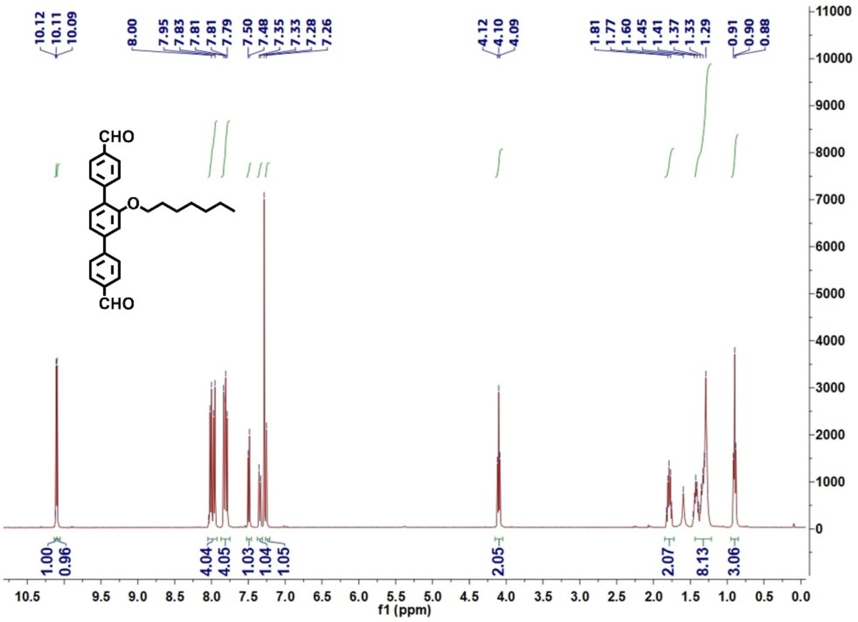

Supplementary Fig. 46. NMR spectrum. ^{1}H NMR spectrum of sHEP-CHO.

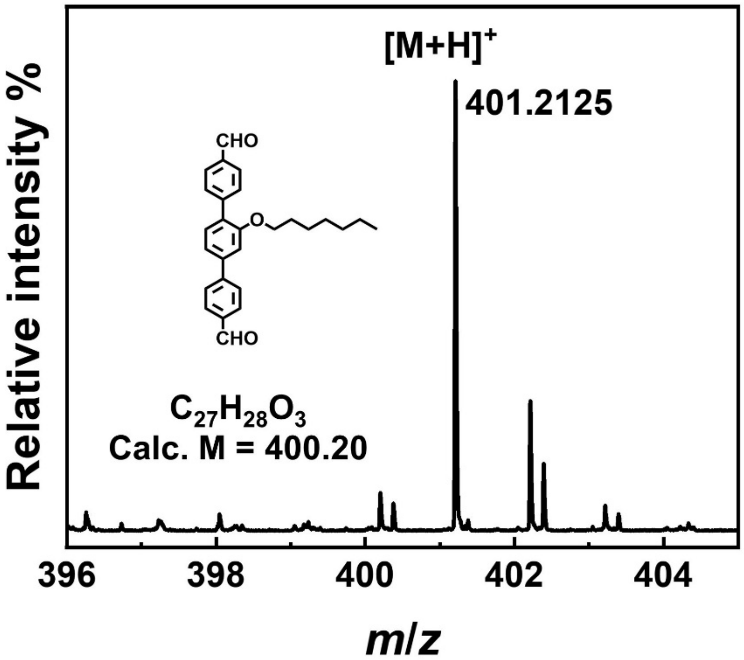

Supplementary Fig. 47. HR-MS spectrum. HR-MS spectrum of sHEP-CHO.

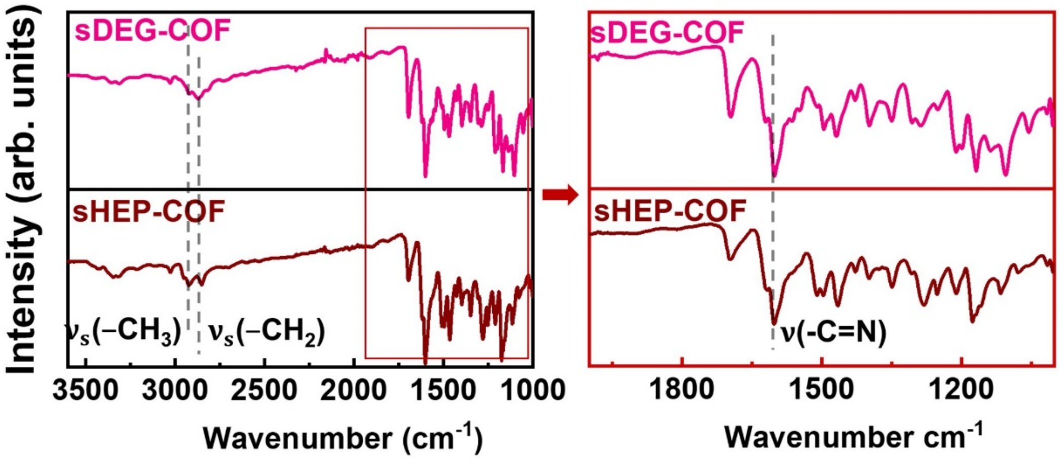

Supplementary Fig. 48. FT-IR spectra. FT-IR spectra of sDEG-COF and sHEP-COF. Source data are provided as a Source Data file.

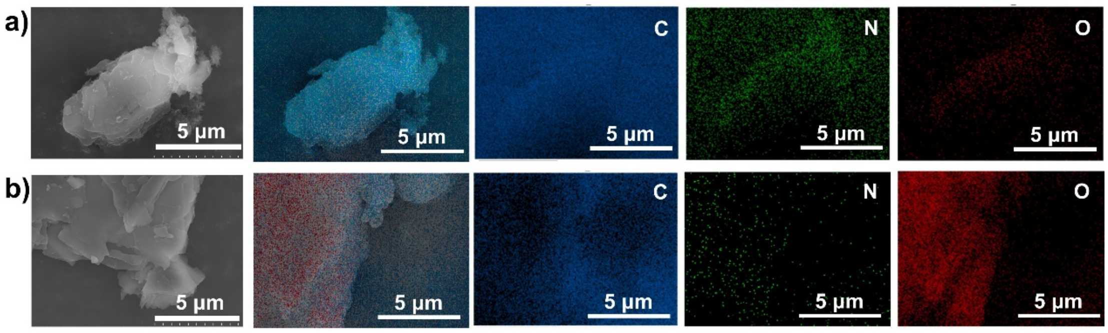

Supplementary Fig. 49. SEM and SEM-EDS. SEM and SEM-EDS images of sDEG-COF (a) and sHEP-COF (b).

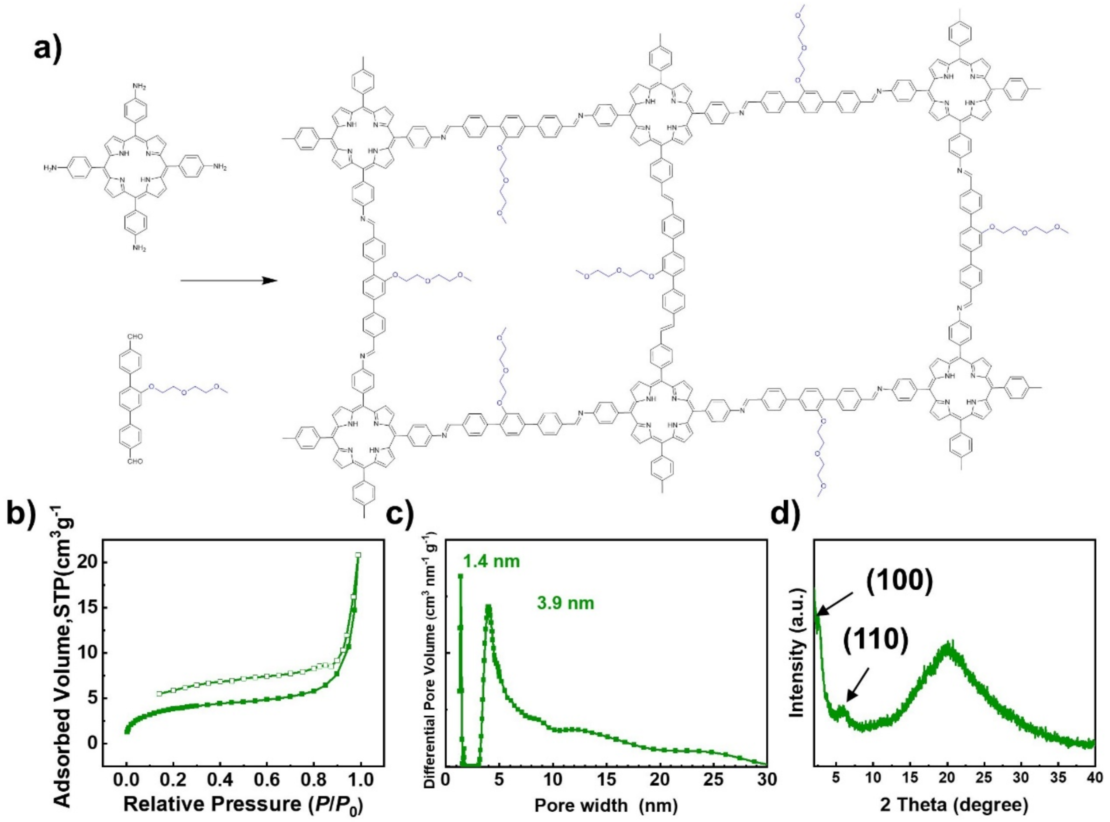

Supplementary Fig. 50. sDEG-COF structure and properties. (a) Schematic illustration of the synthesis of sDEG-COF. (b) N_2 adsorption–desorption isotherm, (c) pore size distribution profile, and (d) PXRD pattern of the sDEG-COF obtained under the optimized synthesis condition (1 mL mesitylene and 200 μL aqueous acetic acid solution (6 M), 120 °C, 72 h). Source data are provided as a Source Data file.

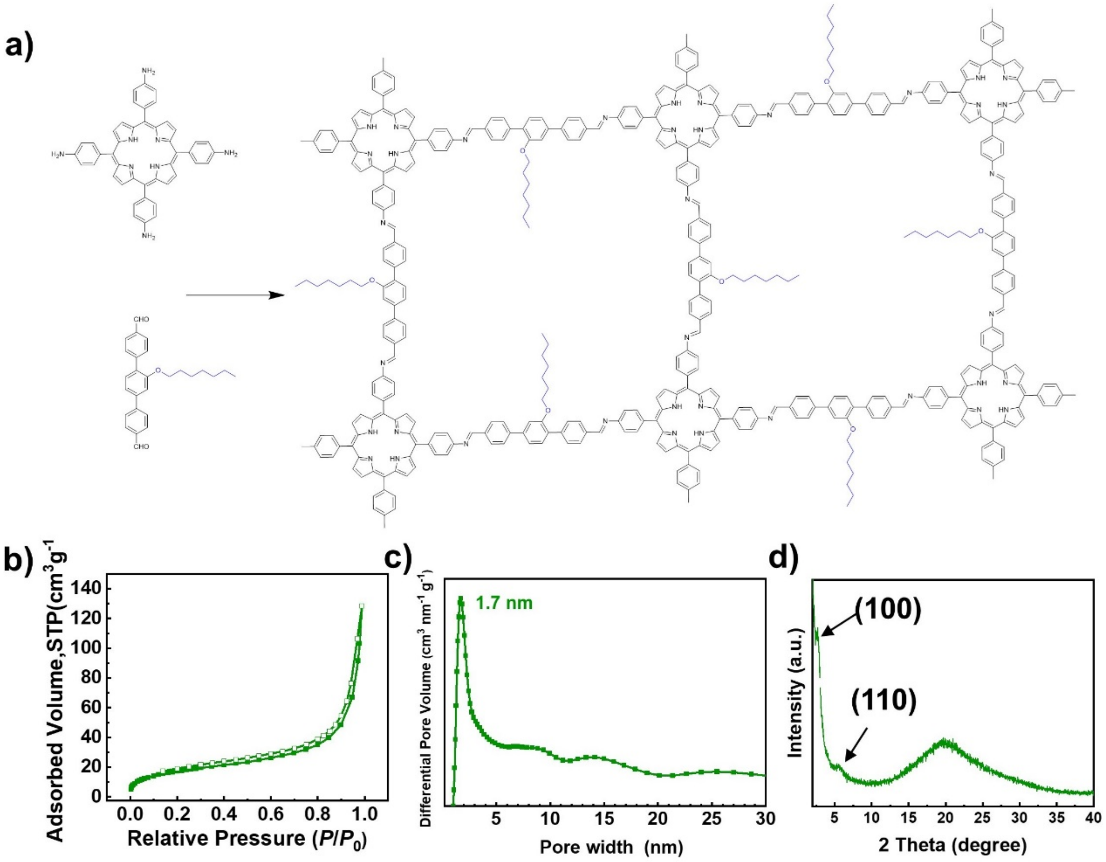

Supplementary Fig. 51. sHEP-COF structure and properties. (a) Schematic illustration of the synthesis of sHEP-COF. (b) N_2 adsorption–desorption isotherm, (c) pore size distribution profile, and (d) PXRD pattern of the sHEP-COF obtained under the optimized synthesis condition (0.5 mL EtOH, 0.5 mL *o*-DCB, and 200 μL aqueous acetic acid solution (6 M), 120 °C, 72 h). Source data are provided as a Source Data file.

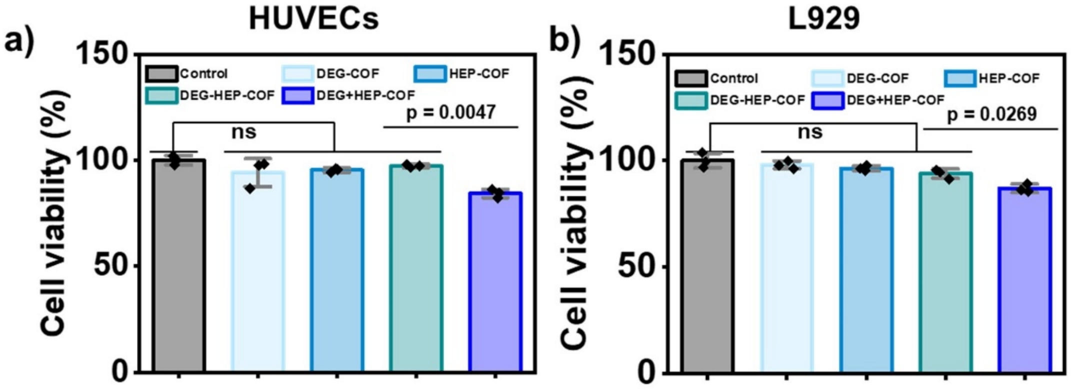

Supplementary Fig. 52 COFs cytoxicity. Evaluation of the cytoxicity of these synthesized COFs on HUVECs (a) and L929 cells (b) by CCK method. For each group, $n = 3$ independent samples. The data are presented as mean ± SD. Statistical analysis was performed using one-way ANOVA analysis and ns means no significant difference. Source data are provided as a Source Data file.

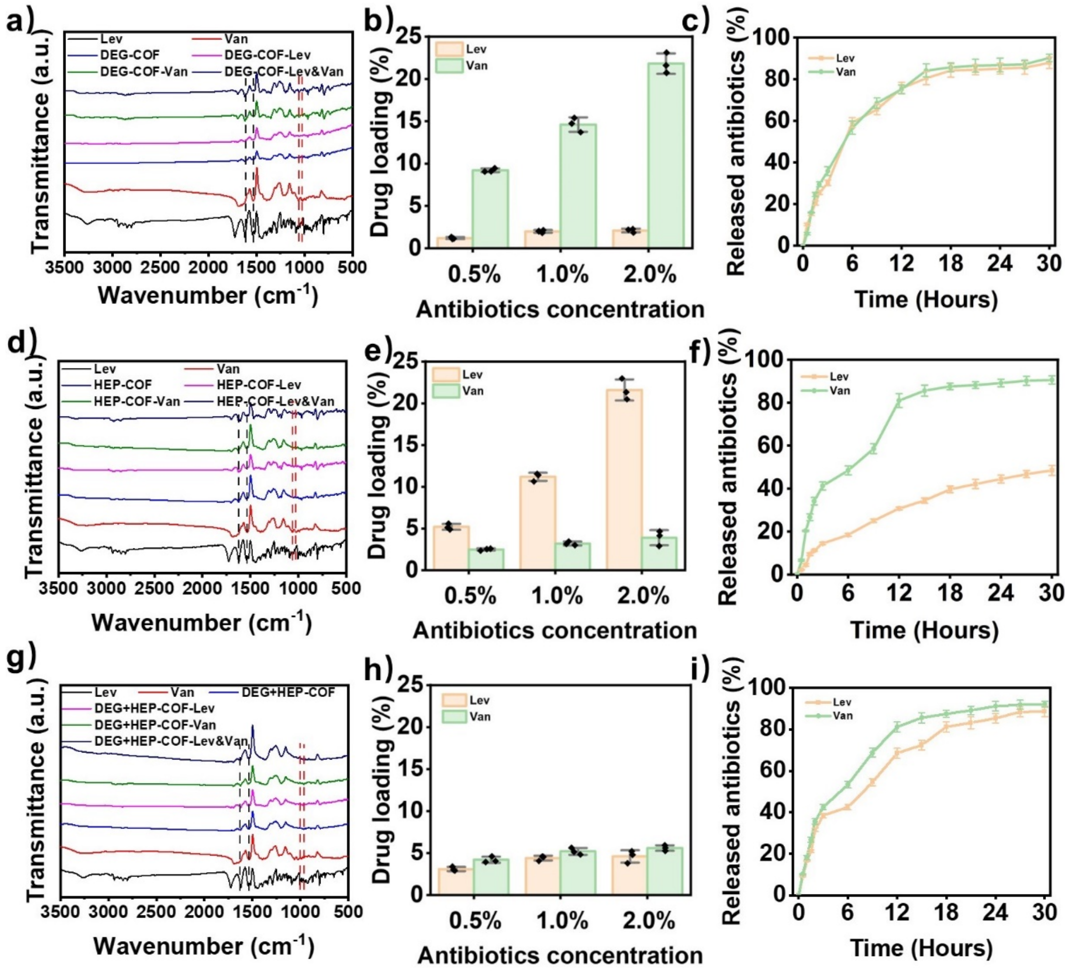

Supplementary Fig. 53. Antibiotic loading/release rates. The FT-IR spectra of DEG-COF (a), HEP-COF (d), and DEG+HEP-COF (g) before and after loading antibiotics. The antibiotic loading rates of DEG-COF (b), HEP-COF (e), and DEG+HEP-COF (h). The antibiotic release efficiencies of DEG-COF (c), HEP-COF (f), and DEG+HEP-COF (i). For each group, $n = 3$ independent samples in (b, e, and h). The data in Fig. 53b, 53e, and 53h are presented as mean ± SD. Source data are provided as a Source Data file.

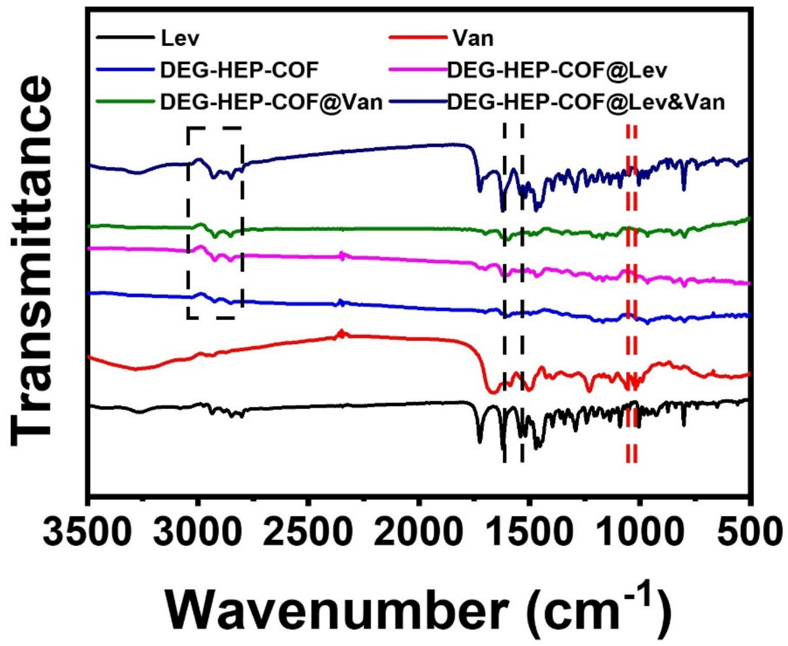

Supplementary Fig. 54. Antibiotic loading. FT-IR plots of DEG-HEP-COF before and after loading antibiotics. The presence of absorption peaks at 1024, 1064, 1539, and 1619 cm^{-1} confirmed that Lev and Van were loaded in DEG-HEP-COF. Source data are provided as a Source Data file.

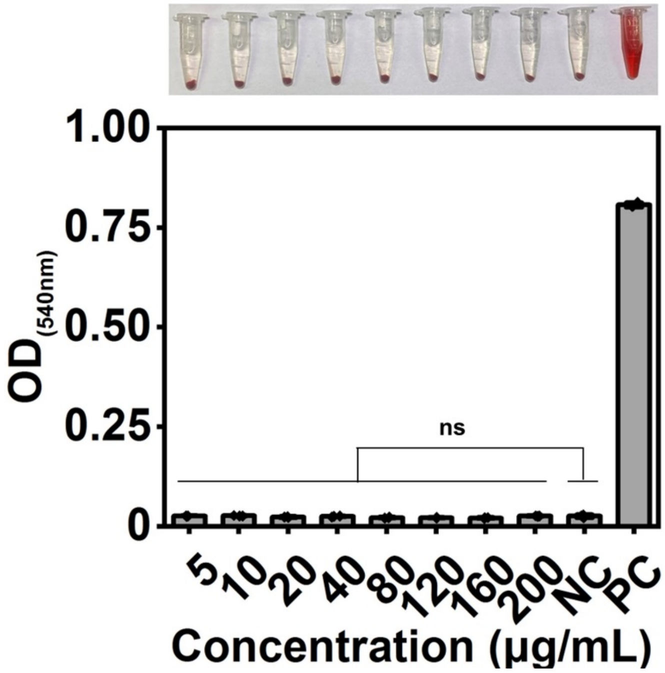

Supplementary Fig. 55. Hemolysis rate. Hemolysis rate of DEG-HEP-COF. For each group, *n* = 3 independent samples. The data are presented as mean ± SD. Statistical analysis was performed using one-way ANOVA analysis and ns means no significant difference. Source data are provided as a Source Data file.

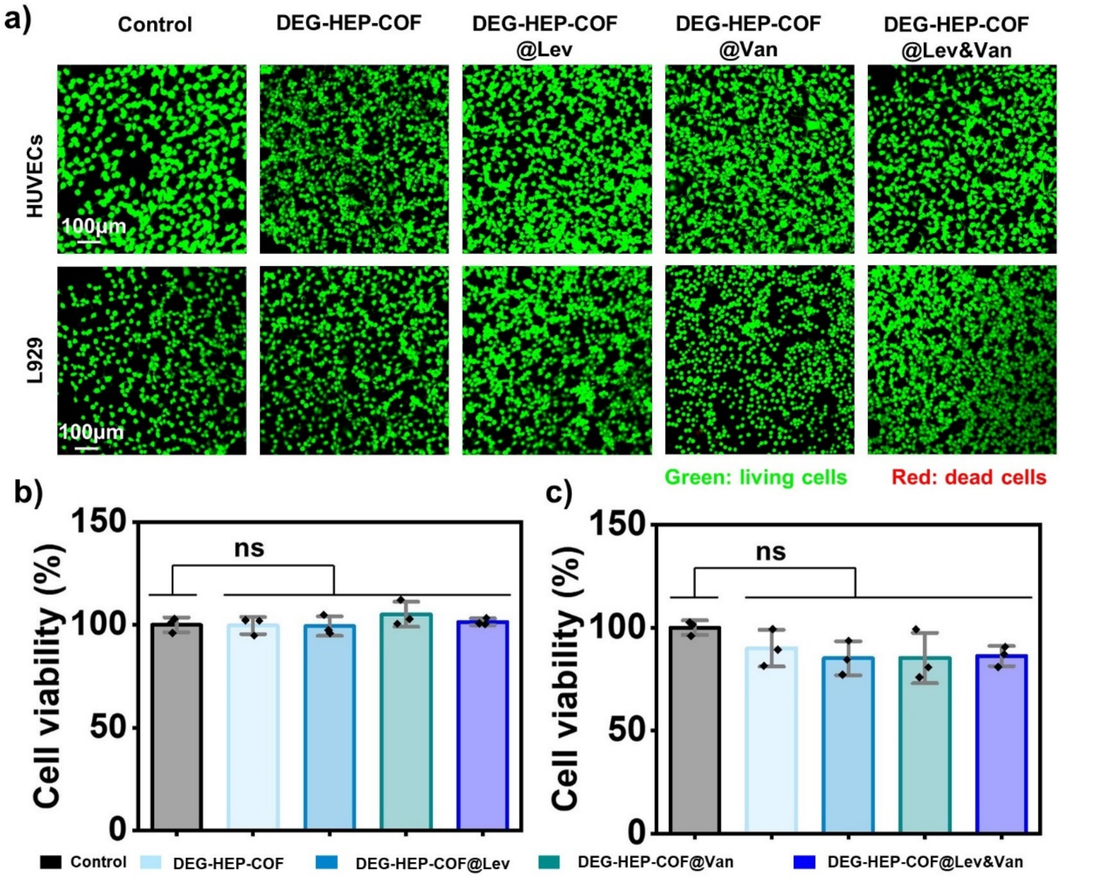

Supplementary Fig. 56. Biological Safety of DEG-HEP-COF@Antibiotics. (a) Evaluation of the cytotoxicity of DEG-HEP-COF@Antibiotics on HUVECs and L929 cells by cell live-dead staining method. After 24 h, numerous live cells (green observed in all groups, and almost no dead cells (red) were observed. (b) Evaluation of the cytoxicity of DEG-HEP-COF@Antibiotics on HUVECs by CCK method. (c) Evaluation of the cytoxicity of DEG-HEP-COF@Antibiotics on L929 cells by CCK method. At a concentration of 100 μg mL^{-1} of DEG-HEP-COF@Antibiotics, the relative cell viability of all DEG-HEP-COF@Antibiotics groups was around 90%. For each group, n = 3 independent samples in (a–c). The data are presented as mean ± SD. Statistical analysis was performed using one-way ANOVA analysis and ns means no significant difference. Source data are provided as a Source Data file.

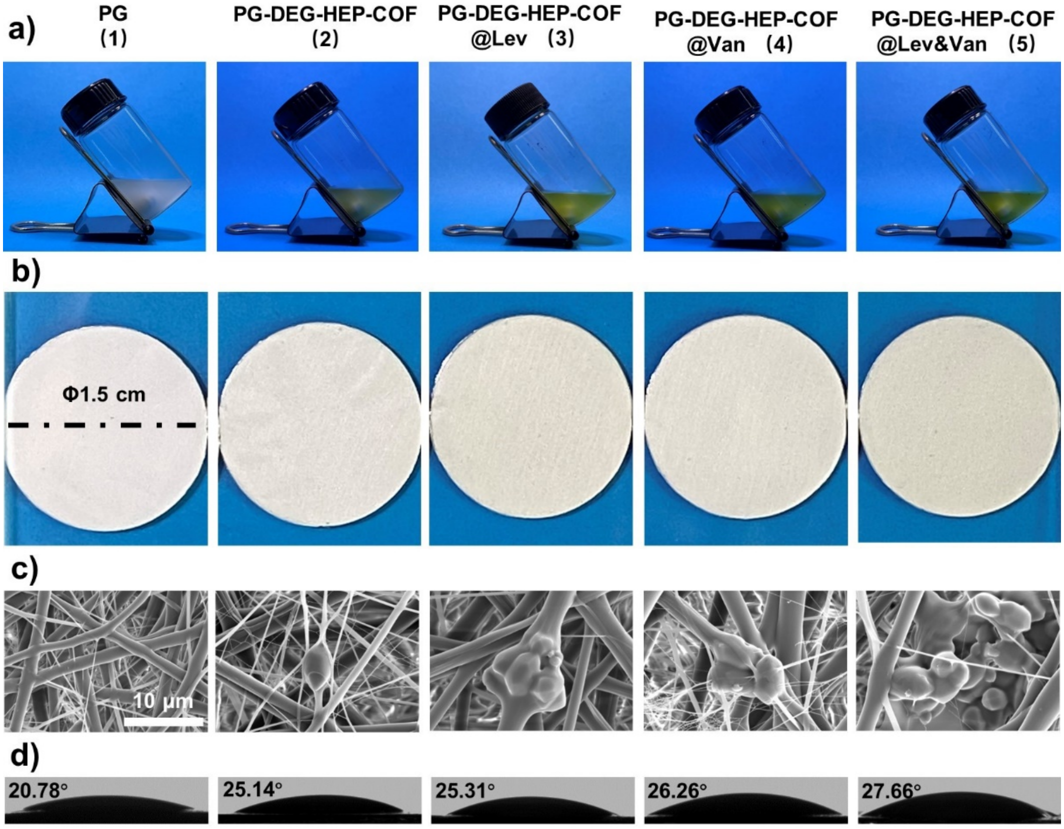

Supplementary Fig. 57. Electrospun membranes preparation and analysis. (a) The DEG-HEP-COF or DEG-HEP-COF@Antibiotics was dispersed in electrospun solution with PCL & gelatin. PCL is approved by the U.S. Food and Drug Administration (FDA) as a biomaterial for tissue engineering applications, with good mechanical property and high biocompatibility. Gelatin possesses good hydrophilicity and degradability. Therefore, the combination of PCL and gelatin can provide sufficient specific surface area for loading DEG-HEP-COF@Antibiotics. (b) Electrospun membranes cut into 0.5 cm diameter after electrostatic spinning. (c) Morphologies of electrospun membranes. SEM images show that the fibers of the membrane are homogeneous and DEG-HEP-COF@Antibiotics could be covered on the fibers. The morphologies of the fibers are not changed after doping with DEG-HEP-COF@Antibiotics. (d) Contact angle of electrospun membranes. Water contact angle analyses show good hydrophilicity of the PG membrane, and the incorporation of DEG-HEP-COF@Antibiotics shows no influence on hydrophilicity.

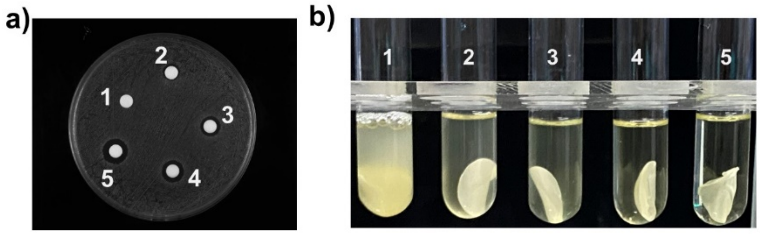

Supplementary Fig. 58. Antibacterial effect. (a) Evaluation of the antibacterial effect of electrospun membranes with/without DEG-HEP-COF@Antibiotics against MRSA by turbidity method. The electrospun membranes were co-cultured with MRSA in the medium for 24 h. The results showed that the medium became turbid in the PG group, and the PG-DEG-HEP-COF group showed slight turbidity, while other groups did not show significant change. (b) Evaluation of the antibacterial effect of electrospun membranes with/without DEG-HEP-COF@Antibiotics against MRSA by disc diffusion method. The diameter of the inhibition zone can be utilized to evaluate the antibacterial activity, and the PG-DEG-HEP-COF@Lev&Van membrane shows the largest diameter of inhibition zone among these tested membranes due to the release of two kinds of antibiotics from PG-DEG-HEP-COF@Lev&Van membrane and the synergistic antibacterial effect of DEG-HEP-COF by itself. (The number of 1, 2, 3, 4, and 5 in the Supplementary Fig. d, e, and f represent the PG, PG-DEG-HEP-COF, PG-DEG-HEP-COF@Lev, PG-DEG-HEP-COF@Van, and PG-DEG-HEP-COF@Lev&Van membranes, respectively).

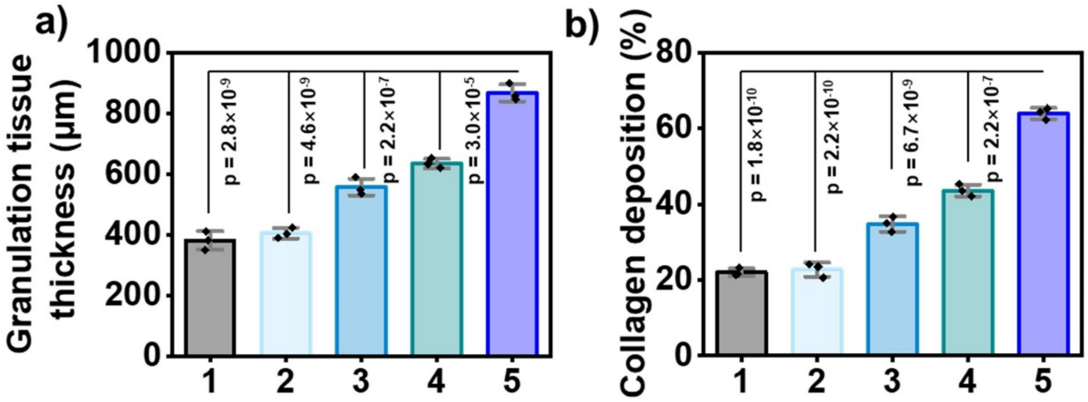

Supplementary Fig. 59. Wound healing assessment. Quantitative analysis of H&E and Masson staining sections acquired from different groups to calculate the granulation tissue thickness and collagen deposition (The number of 1, 2, 3, 4, and 5 represent the PG, PG-DEG-HEP-COF, PG-DEG-HEP-COF@Lev, PG-DEG-HEP-COF@Van, and PG-DEG-HEP-COF@Lev&Van membranes, respectively.). For each group, n = 3 independent samples in (a, b). The data are presented as mean ± SD. Statistical analysis was performed using one-way ANOVA analysis and ns means no significant difference. Source data are provided as a Source Data file.

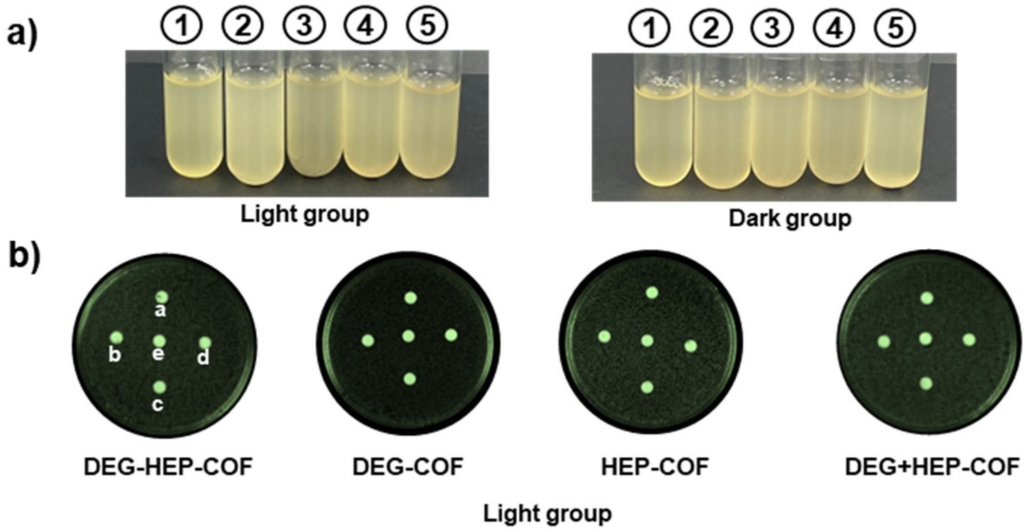

Supplementary Fig. 60. COFs antibacterial effect. (a) Evaluation of the antibacterial effect of COFs (100 μg mL^{-1}) against MRSA by turbidity method. (b) Evaluation of the antibacterial effect of COFs against MRSA by disc diffusion method. The diameter of the inhibition zone can be utilized to evaluate the antibacterial activity. (The numbers 1, 2, 3, 4, and 5 represent the blank, DEG-HEP-COF, DEG-COF, HEP-COF, and DEG+HEP-COF, respectively. The a, b, c, d, and e represent the COFs concentration of 50, 100, 200, 400, 1000 μg·mL^{-1}, respectively).

Supplementary Table 1. The assignment of wavenumber peaks in FT-IR spectra (**Supplementary Fig. 1**).

Wavenumber (cm^{-1})	DEG-HEP-CHO	DEG-CHO	HEP-CHO	sDEF-CHO	sHEP-CHO
$v_s(-CH_3)$, $v_{as}(-CH_3)$	2930, 2813	2825	2925, 2795	2881	2922, 2805
$v_s(-CH_2)$, $v_{as}(-CH_2)$	2860, 2725	2875, 2739	2861, 2710	2885, 2735	2853, 2740
$v(-C{=}O)$	1701	1683	1696	1689	1692
$v(-C{=}C)$	1602	1597	1594	1594	1602
$v(C{-}O{-}C)$	1105	1102	–	1096	–

Supplementary Table 2. The optimization of synthesis conditions for DEG-HEP-COF.

No.	Solvent (mL)	Temp (°C)	Time (h)
1	*o*-DCB:EtOH:HOAc (6 M) = 0:1.0:0.2	120	72
2	*o*-DCB:EtOH:HOAc (6 M) = 0.1:0.9:0.2	120	72
3	*o*-DCB:EtOH:HOAc (6 M) = 0.3:0.7:0.2	120	72
4	*o*-DCB:EtOH:HOAc (6 M) = 0.5:0.5:0.2	120	72
5	*o*-DCB:EtOH:HOAc (6 M) = 0.7:0.3:0.2	120	72
6	*o*-DCB:EtOH:HOAc (6 M) = 0.9:0.1:0.2	120	72
7	*o*-DCB:EtOH:HOAc (6 M) = 0.2:1.8:0.2	120	72
8	*o*-DCB:EtOH:HOAc (6 M) = 1.0:1.0:0.2	120	72
9	*o*-DCB:EtOH:HOAc (6 M) = 0.1:0.9:0.2	80	72
10	*o*-DCB:EtOH:HOAc (6 M) = 0.1:0.9:0.2	100	72
11	*o*-DCB:EtOH:HOAc (6 M) = 0.1:0.9:0.2	120	72
12	1,4-Dioxane:EtOH:HOAc (6 M) = 0.1:0.9:0.2	120	72
13	1,4-Dioxane:EtOH:HOAc (6 M) = 0.5:0.5:0.2	120	72
14	1,4-Dioxane:EtOH:HOAc (6 M) = 0.9:0.1:0.2	120	72
15	Mesitylene:EtOH:HOAc (6 M) =0.1:0.9:0.2	120	72
16	Mesitylene:EtOH:HOAc (6 M) =0.5:0.5:0.2	120	72
17	Mesitylene:EtOH:HOAc (6 M) =0.9:0.1:0.2	120	72

Supplementary Table 3. The optimization of synthesis conditions for DEG-COF.

No.	Solvent (mL)	Temperature (°C)	Time (h)
1	*o*-DCB:EtOH:HOAc (6 M) = 0:1.0:0.2	80	72
2	*o*-DCB:EtOH:HOAc (6 M) = 0:1.0:0.2	100	72
3	*o*-DCB:EtOH:HOAc (6 M) = 0:1.0:0.2	120	72

Supplementary Table 4. The optimization of synthesis conditions for HEP-COF.

No.	Solvent (mL)	Temperature (°C)	Time (h)
1	*o*-DCB:EtOH:HOAc (6 M) = 0.3:0.7:0.2	80	72
2	*o*-DCB:EtOH:HOAc (6 M) = 0.5:0.5:0.2	80	72
3	*o*-DCB:EtOH:HOAc (6 M) = 0.7:0.3:0.2	80	72
4	*o*-DCB:EtOH:HOAc (6 M) = 0.9:0.1:0.2	80	72
5	*o*-DCB:EtOH:HOAc (6 M) = 0.3:0.7:0.2	120	72
6	*o*-DCB:EtOH:HOAc (6 M) = 0.5:0.5:0.2	120	72
7	*o*-DCB:EtOH:HOAc (6 M) = 0.7:0.3:0.2	120	72
8	*o*-DCB:EtOH:HOAc (6 M) = 0.9:0.1:0.2	120	72
9	*o*-DCB:EtOH:HOAc (6 M) = 0.7:0.3:0.2	100	72

Supplementary Table 5. The optimization of synthesis conditions for DEG+HEP-COF.

No.	Solvent (mL)	Temperature (°C)	Time (h)
1	*o*-DCB:EtOH:HOAc (6 M) = 1.0:1.0:0.2	120	72
2	*o*-DCB:EtOH:HOAc (6 M) = 0.5:0.5:0.2	120	72
3	*o*-DCB:EtOH:HOAc (6 M) = 0.2:1.8:0.2	120	72
4	*o*-DCB:EtOH:HOAc (6 M) = 1.8:0.2:0.2	120	72

Supplementary Table 6. Elemental analysis data of DEG-HEP-COF, DEG-COF, HEP-COF, DEG+HEP-COF.

COF		C%	N%	H%
DEG-HEP-COF	Calculated	80.66	6.96	6.34
	Found	79.01	7.08	6.35
DEG-COF	Calculated	75.89	6.80	5.59
	Found	73.41	7.14	5.64
HEP-COF	Calculated	82.52	6.63	6.99
	Found	80.32	6.94	6.72
DEG+HEP-COF	Calculated	77.58	7.23	6.84
	Found	77.71	6.78	6.24

Supplementary Table 7. Fractional atomic coordinates for contorted AA stacking mode of DEG-HEP-COF.

**DEG-HEP-COF**<br>**Space group: *P*1**<br>**Unit cell parameters: *a* = 32.4 Å, *b* = 47.6 Å, *c* = 11.9 Å; *α* = *β* = *γ* = 90°.**			
**Atom**	**x**	**y**	**z**
C1	0.41361	0.43192	0.16087
C2	0.38768	0.41064	0.13897
C3	0.85352	0.9446	0.23722
C4	0.82442	0.92581	0.21186
C5	0.61817	0.57713	–0.03494
C6	0.63965	0.59524	–0.10093
C7	0.07907	0.05394	–0.13639
C8	0.10512	0.06751	–0.20792
C9	0.6506	0.41969	0.23623
C10	0.67196	0.399	0.18276
C11	0.0999	0.90526	0.23356
C12	0.12287	0.88542	0.17996
C13	0.39639	0.59353	0.2232
C14	0.36978	0.61095	0.16928
C15	0.87019	0.09151	0.18133
C16	0.84185	0.1073	0.12629
C17	0.64504	0.59742	0.13482
C18	0.66674	0.61549	0.06862
C19	0.11993	0.06529	0.0241
C20	0.14605	0.07854	–0.0472
C21	0.41696	0.41824	0.35707
C22	0.39111	0.39677	0.33494
C23	0.87033	0.91906	0.40519
C24	0.84104	0.90003	0.37925
C25	0.4079	0.57033	0.04578
C26	0.38172	0.5878	–0.00803
C27	0.87007	0.05993	0.02393
C28	0.84205	0.07573	–0.03111
C29	0.637	0.44014	0.05442
C30	0.65802	0.41952	0.00128
C31	0.07409	0.91913	0.05186
C32	0.09702	0.89955	–0.00127

N33	0.53198	0.46626	0.26901
N34	0.52305	0.55222	0.20095
N35	–0.01399	0.95993	0.28869
N36	–0.01119	0.03978	0.13365
C37	0.24259	0.78087	–0.31837
C38	0.2839	0.7797	–0.32791
C39	0.30351	0.75468	–0.32875
C40	0.70719	0.26704	–0.31709
C41	0.74607	0.25843	–0.33822
C42	0.75514	0.23076	–0.34829
N43	0.34751	0.34753	0.25974
C44	0.51302	0.42206	0.26787
C45	0.49924	0.44976	0.27785
C46	0.45934	0.45823	0.28478
C47	0.44486	0.48547	0.28099
C48	0.40295	0.49229	0.28266
C49	0.42916	0.43577	0.26987
C50	0.37661	0.39235	0.22507
C51	0.35325	0.36825	0.19382
C52	0.32893	0.32415	0.21284
C53	0.34793	0.29867	0.22282
C54	0.33398	0.27619	0.16288
C55	0.29998	0.2782	0.09524
C56	0.27909	0.30312	0.09481
C57	0.29337	0.32593	0.15199
N58	0.78088	0.88192	0.13232
C59	0.9591	0.91727	0.31145
C60	0.95108	0.94617	0.31694
C61	0.91424	0.95915	0.33594
C62	0.90689	0.98783	0.32432
C63	0.869	1.00056	0.35012
C64	0.87842	0.94097	0.33162
C65	0.81879	0.90237	0.27933
C66	0.79502	0.8797	0.23326
C67	0.76794	0.86063	0.0626
C68	0.79106	0.83676	0.04977
C69	0.78196	0.81774	–0.03341
C70	0.74932	0.82138	–0.10546
C71	0.72607	0.84505	–0.0911
C72	0.73554	0.86461	–0.01023
C73	0.72576	0.21057	–0.33773
C74	0.68631	0.21944	–0.33228
C75	0.67724	0.24711	–0.32216
C76	0.2826	0.72984	–0.31982
C77	0.24123	0.73104	–0.32594

C78	0.22165	0.75604	–0.32528
N79	0.67711	0.64044	–0.22305
C80	0.53819	0.59373	0.13018
C81	0.55412	0.56763	0.16225
C82	0.59381	0.55904	0.15006
C83	0.60943	0.53297	0.17706
C84	0.65055	0.5254	0.15941
C85	0.61992	0.57829	0.08376
C86	0.66386	0.6151	–0.05
C87	0.68294	0.63678	–0.11595
C88	0.69242	0.6648	–0.27123
C89	0.66652	0.68229	–0.32985
C90	0.67727	0.70931	–0.35074
C91	0.71444	0.71931	–0.31728
C92	0.74228	0.70015	–0.27723
C93	0.73162	0.67327	–0.25399
N94	0.19614	0.10792	–0.18588
C95	0.00458	0.07491	0.01923
C96	0.01912	0.05023	0.0699
C97	0.0573	0.03863	0.05863
C98	0.06988	0.01389	0.10962
C99	0.10885	0.00177	0.09418
C100	0.08569	0.05293	–0.01912
C101	0.13904	0.08024	–0.16438
C102	0.16461	0.09827	–0.23221
C103	0.21658	0.13197	–0.2088
C104	0.19571	0.15622	–0.23619
C105	0.2123	0.18167	–0.21455
C106	0.25031	0.18399	–0.16763
C107	0.27302	0.15986	–0.15736
C108	0.25653	0.13428	–0.17643
C109	0.73901	0.7992	–0.1866
C110	0.7001	0.78948	–0.19415
C111	0.69179	0.7649	–0.24823
C112	0.2871	0.25485	0.02327
C113	0.24741	0.24679	0.01851
C114	0.23587	0.2247	–0.04751
N115	0.687	0.37094	–0.10221
C116	0.55282	0.42269	0.24934
C117	0.56514	0.45081	0.24776
C118	0.60314	0.46088	0.22003
C119	0.61361	0.48888	0.20859
C120	0.65318	0.49814	0.1793
C121	0.63163	0.44007	0.17246
C122	0.67482	0.39799	0.06424

C123	0.6901	0.37356	0.00612
C124	0.69245	0.3457	–0.15732
C125	0.67236	0.32227	–0.11812
C126	0.67573	0.29756	–0.17517
C127	0.69949	0.29505	–0.27215
C128	0.71839	0.31874	–0.31285
C129	0.71434	0.34382	–0.258
N130	0.15052	0.8634	–0.10673
C131	–0.0024	0.91456	0.27731
C132	0.01469	0.94113	0.26073
C133	0.0524	0.9467	0.21597
C134	0.06735	0.97268	0.18212
C135	0.10733	0.97638	0.13938
C136	0.07575	0.92276	0.16986
C137	0.12247	0.88265	0.0617
C138	0.14817	0.86312	0.0023
C139	0.1754	0.84543	–0.16644
C140	0.16056	0.8321	–0.26298
C141	0.18301	0.81235	–0.31789
C142	0.22143	0.80603	–0.28113
C143	0.23747	0.82168	–0.19225
C144	0.21496	0.84101	–0.13513
C145	0.26333	0.20979	–0.11227
C146	0.30284	0.21843	–0.11014
C147	0.31445	0.24047	–0.04358
C148	0.72193	0.74901	–0.29749
C149	0.75986	0.76059	–0.30274
C150	0.76821	0.78514	–0.2482
N151	0.34188	0.63222	–0.11721
C152	0.49823	0.59296	0.14648
C153	0.48822	0.56637	0.18926
C154	0.44974	0.55606	0.20837
C155	0.44036	0.52937	0.24589
C156	0.40018	0.51942	0.26034
C157	0.41679	0.57357	0.16119
C158	0.36316	0.60905	0.05219
C159	0.34151	0.63068	–0.00815
C160	0.32852	0.65589	–0.17353
C161	0.34298	0.6818	–0.14111
C162	0.33002	0.70528	–0.19574
C163	0.30204	0.70394	–0.28351
C164	0.28968	0.67784	–0.3199
C165	0.30324	0.6541	–0.26749
N166	0.79404	0.11514	–0.15071
C167	0.96592	0.07739	0.05309

C168	0.95558	0.055	0.12549
C169	0.91839	0.05059	0.17723
C170	0.90838	0.02899	0.25141
C171	0.86982	0.02595	0.30415
C172	0.88531	0.06788	0.1296
C173	0.82804	0.10009	0.01821
C174	0.80206	0.11872	–0.04484
C175	0.77226	0.13542	–0.20905
C176	0.78763	0.14606	–0.31031
C177	0.77096	0.16931	–0.35879
C178	0.73767	0.18192	–0.31043
C179	0.7195	0.16875	–0.21818
C180	0.73646	0.14593	–0.16749
N181	0.46683	0.50856	0.26326
N182	0.58813	0.51062	0.21152
N183	0.93103	0.00612	0.2714
N184	0.04599	–0.00387	0.16803
O185	–0.34229	0.2025	–0.31701
C186	–0.32653	0.17203	–0.30896
C187	–0.35348	0.15553	–0.22509
O188	–0.33766	0.12508	–0.2168
C189	–0.36447	0.10862	–0.13246
C190	–0.34855	0.0782	–0.12382
O191	–0.37527	0.06178	–0.03915
C192	–0.35934	0.03136	–0.03046
C193	–0.21904	0.27787	–0.37973
C194	–0.23612	0.30156	–0.30463
C195	–0.20038	0.32071	–0.26678
C196	–0.21744	0.34436	–0.19144
C197	–0.18167	0.36342	–0.15308
C198	–0.1987	0.38701	–0.07737
C199	–0.1629	0.406	–0.03868
C200	–0.64138	0.04415	–0.41817
O201	–0.62384	0.07271	–0.38051
C202	–0.65567	0.08782	–0.30544
C203	–0.63809	0.11635	–0.26762
O204	–0.66987	0.13143	–0.19231
C205	–0.65221	0.1599	–0.15398
C206	–0.68392	0.17493	–0.0783
O207	–0.66617	0.20334	–0.03964
C208	–0.83045	0.39182	–0.38091
C209	–0.79824	0.37709	–0.30571
C210	–0.81508	0.34835	–0.26796
C211	–0.78291	0.33366	–0.19253
C212	–0.79985	0.30498	–0.15426

C213	–0.76776	0.29033	–0.07846
C214	–0.78477	0.26171	–0.03987
C215	–0.20211	0.74337	–0.01852
C216	–0.2161	0.71878	–0.09368
C217	–0.17804	0.70177	–0.13139
C218	–0.192	0.67722	–0.20679
C219	–0.15392	0.66029	–0.24501
C220	–0.16786	0.63581	–0.32078
C221	–0.12975	0.61895	–0.35933
C222	0.63378	0.96269	–0.41573
O223	0.61772	0.93373	–0.37813
C224	0.6503	0.91939	–0.30293
C225	0.6342	0.89045	–0.26518
O226	0.66674	0.87614	–0.18975
C227	0.65054	0.84727	–0.15148
C228	0.68301	0.833	–0.07568
O229	0.66673	0.80417	–0.03708
C230	0.21695	0.70526	–0.3789
C231	0.24492	0.68696	–0.30371
C232	0.22098	0.66065	–0.26597
C233	0.24891	0.64238	–0.19055
C234	0.2249	0.61615	–0.1523
C235	0.25277	0.59793	–0.0765
C236	0.2287	0.57176	–0.03792
C237	0.2356	0.95565	0.01995
O238	0.26817	0.93398	–0.01762
C239	0.24754	0.91167	–0.09279
C240	0.28013	0.89003	–0.13051
O241	0.25953	0.86775	–0.20592
C242	0.29217	0.8462	–0.24416
C243	0.27161	0.82398	–0.31993
O244	0.30429	0.8025	–0.3585
H245	0.42304	0.44693	0.09067
H246	0.3749	0.40766	0.0503
H247	0.85814	0.96381	0.18133
H248	0.80407	0.929	0.13448
H249	0.5979	0.56031	–0.0751
H250	0.63728	0.59369	–0.19651
H251	0.05056	0.04279	–0.17071
H252	0.09819	0.06798	–0.30203
H253	0.64871	0.41989	0.33208
H254	0.6881	0.38193	0.2338
H255	0.10106	0.90701	0.32925
H256	0.14315	0.87046	0.23072
H257	0.40136	0.59568	0.31785

H258	0.35214	0.62762	0.2187
H259	0.88104	0.09794	0.26897
H260	0.82867	0.12696	0.16776
H261	0.64816	0.59853	0.23034
H262	0.68815	0.63167	0.10868
H263	0.12697	0.0647	0.11814
H264	0.17523	0.08896	–0.01332
H265	0.42855	0.42183	0.44659
H266	0.38095	0.38214	0.40565
H267	0.88833	0.91711	0.48738
H268	0.8344	0.88189	0.43924
H269	0.4234	0.55244	–0.00214
H270	0.37531	0.58482	–0.10167
H271	0.88188	0.0396	–0.01541
H272	0.83029	0.06886	–0.11732
H273	0.62307	0.45819	0.00389
H274	0.66166	0.42007	–0.09416
H275	0.05257	0.93335	0.00145
H276	0.09503	0.89729	–0.09669
H277	–0.00581	0.0205	0.18418
H278	0.33859	0.7538	–0.3371
H279	0.78824	0.22384	–0.36615
H280	0.49019	0.40396	0.27622
H281	0.37899	0.47536	0.30111
H282	0.33941	0.36801	0.10552
H283	0.3756	0.29613	0.28147
H284	0.35077	0.25517	0.16867
H285	0.24893	0.3052	0.04578
H286	0.27545	0.34655	0.1488
H287	0.93392	0.90159	0.33479
H288	0.84499	0.98869	0.40174
H289	0.78858	0.85994	0.28453
H290	0.81823	0.8325	0.10854
H291	0.80186	0.79814	–0.04421
H292	0.69799	0.84895	–0.14702
H293	0.71589	0.88445	–0.00425
H294	0.6433	0.25348	–0.31791
H295	0.18646	0.75603	–0.33073
H296	0.55977	0.61074	0.09712
H297	0.67405	0.54197	0.13294
H298	0.70452	0.65138	–0.06766
H299	0.63551	0.67417	–0.36172
H300	0.65491	0.72383	–0.39689
H301	0.77577	0.7066	–0.26231
H302	0.75556	0.65784	–0.22005

H303	0.02586	0.08774	–0.03732
H304	0.13423	0.01404	0.04939
H305	0.15741	0.10388	–0.32346
H306	0.16398	0.15499	–0.27771
H307	0.19427	0.20157	–0.23581
H308	0.30697	0.161	–0.13197
H309	0.27626	0.11461	–0.16528
H310	0.65865	0.75689	–0.25361
H311	0.20211	0.2179	–0.05054
H312	0.57072	0.40224	0.23794
H313	0.68417	0.48691	0.17083
H314	0.70526	0.35623	0.05803
H315	0.65297	0.32356	–0.03816
H316	0.65877	0.27811	–0.14369
H317	0.73766	0.31761	–0.39306
H318	0.72918	0.36338	–0.29574
H319	0.01698	0.89496	0.26106
H320	0.13118	0.95886	0.14791
H321	0.16678	0.84712	0.05274
H322	0.12875	0.83774	–0.29767
H323	0.16944	0.80123	–0.39444
H324	0.27091	0.81849	–0.16468
H325	0.22874	0.85341	–0.06209
H326	0.34843	0.24677	–0.04511
H327	0.80089	0.79391	–0.25523
H328	0.47844	0.61211	0.1259
H329	0.36858	0.53	0.25628
H330	0.32355	0.64685	0.0431
H331	0.36584	0.68358	–0.06838
H332	0.34225	0.72675	–0.16944
H333	0.26782	0.67588	–0.3948
H334	0.29367	0.63267	–0.30138
H335	0.94194	0.09417	0.03224
H336	0.84639	0.04383	0.30126
H337	0.78821	0.13736	0.0022
H338	0.81468	0.13521	–0.35387
H339	0.78488	0.17813	–0.43954
H340	0.68918	0.17709	–0.18241
H341	0.72122	0.13563	–0.09148
H342	0.96382	0.00533	0.2441
H343	–0.29309	0.17201	–0.27862
H344	–0.32835	0.1617	–0.39542
H345	–0.38691	0.15551	–0.25554
H346	–0.35172	0.1659	–0.13868
H347	–0.39793	0.10852	–0.16264

H348	–0.36265	0.11907	–0.04623
H349	–0.31506	0.0783	–0.09394
H350	–0.35049	0.06772	–0.20999
H351	–0.36025	0.02111	–0.11719
H352	–0.37977	0.01901	0.02999
H353	–0.3262	0.03144	0.00224
H354	–0.18716	0.27042	–0.35159
H355	–0.21947	0.28493	–0.47145
H356	–0.2517	0.29204	–0.22748
H357	–0.25965	0.31443	–0.35421
H358	–0.18486	0.33026	–0.34392
H359	–0.17682	0.30782	–0.21738
H360	–0.23312	0.3348	–0.11453
H361	–0.24088	0.35731	–0.24098
H362	–0.16607	0.37304	–0.22997
H363	–0.15817	0.35045	–0.10382
H364	–0.2144	0.37738	–0.00062
H365	–0.22211	0.40003	–0.12672
H366	–0.14797	0.41626	–0.11522
H367	–0.1389	0.39282	0.00783
H368	–0.17532	0.42297	0.02001
H369	–0.62563	0.0266	–0.36886
H370	–0.63542	0.04111	–0.51199
H371	–0.67607	0.04361	–0.40155
H372	–0.6625	0.07436	–0.22827
H373	–0.68541	0.09133	–0.35503
H374	–0.63134	0.12983	–0.34478
H375	–0.60831	0.11284	–0.21821
H376	–0.64544	0.17346	–0.23089
H377	–0.62241	0.15629	–0.10471
H378	–0.69077	0.16133	–0.00154
H379	–0.71367	0.17861	–0.12766
H380	–0.8571	0.3768	–0.39865
H381	–0.81539	0.39826	–0.46372
H382	–0.84271	0.41128	–0.33601
H383	–0.79176	0.39063	–0.22852
H384	–0.76841	0.37393	–0.35519
H385	–0.82149	0.3348	–0.34514
H386	–0.84495	0.35151	–0.21866
H387	–0.77638	0.34726	–0.11557
H388	–0.7531	0.33039	–0.24197
H389	–0.80627	0.29134	–0.2312
H390	–0.82973	0.30824	–0.10511
H391	–0.76125	0.304	–0.00167
H392	–0.73792	0.287	–0.12771

H393	–0.79509	0.24905	–0.11636
H394	–0.81218	0.26516	0.0188
H395	–0.16733	0.74542	–0.00548
H396	–0.22168	0.74385	0.06125
H397	–0.23284	0.72736	–0.17089
H398	–0.23789	0.7046	–0.04418
H399	–0.16134	0.69314	–0.05419
H400	–0.15621	0.71596	–0.1807
H401	–0.20884	0.68584	–0.28376
H402	–0.21369	0.66296	–0.15733
H403	–0.13714	0.6516	–0.16805
H404	–0.13217	0.67457	–0.29418
H405	–0.18473	0.64449	–0.39758
H406	–0.18951	0.62148	–0.27152
H407	–0.12641	0.62071	–0.45462
H408	–0.13374	0.59595	–0.33497
H409	–0.10089	0.62772	–0.31693
H410	0.63585	0.96332	–0.51152
H411	0.66572	0.96644	–0.37806
H412	0.61165	0.97974	–0.38544
H413	0.65643	0.933	–0.22574
H414	0.68021	0.91658	–0.35241
H415	0.62814	0.87682	–0.34236
H416	0.60426	0.89326	–0.21588
H417	0.64448	0.83355	–0.22842
H418	0.62059	0.85017	–0.10233
H419	0.68916	0.84675	0.00111
H420	0.71293	0.83002	–0.12492
H421	0.18305	0.70458	–0.35291
H422	0.22201	0.6994	–0.47095
H423	0.25476	0.69952	–0.22651
H424	0.27349	0.68033	–0.35319
H425	0.21121	0.64806	–0.34316
H426	0.19237	0.66729	–0.21667
H427	0.25882	0.65501	–0.11359
H428	0.27744	0.63565	–0.23999
H429	0.2151	0.60348	–0.22924
H430	0.1963	0.62288	–0.10314
H431	0.26267	0.61063	0.00029
H432	0.28131	0.59113	–0.12575
H433	0.25075	0.55649	0.00535
H434	0.2141	0.56111	–0.11416
H435	0.20342	0.5783	0.0236
H436	0.21039	0.94463	0.07031
H437	0.22134	0.96605	–0.05736

H438	0.25095	0.97232	0.07472
H439	0.23351	0.92226	–0.16999
H440	0.22216	0.90058	–0.0433
H441	0.2941	0.87941	–0.05331
H442	0.30554	0.90113	–0.17982
H443	0.3062	0.8355	–0.1672
H444	0.31753	0.85739	–0.29332
H445	0.25748	0.83469	–0.39674
H446	0.24632	0.81273	–0.27068

Supplementary Table 8. Fractional atomic coordinates for contorted AA stacking mode of DEG-COF.

**DEG-COF**<br>**Space group: *P*1**<br>**Unit cell parameters: *a* = 32.4 Å, *b* = 47.6 Å, *c* = 11.9 Å; *α* = *β* = *γ* = 90°.**			
**Atom**	**x**	**y**	**z**
C1	0.41361	0.43192	0.16087
C2	0.38768	0.41064	0.13897
C3	0.85352	0.9446	0.23722
C4	0.82442	0.92581	0.21186
C5	0.61817	0.57713	–0.03494
C6	0.63965	0.59524	–0.10093
C7	0.07907	0.05394	–0.13639
C8	0.10512	0.06751	–0.20792
C9	0.6506	0.41969	0.23623
C10	0.67196	0.399	0.18276
C11	0.0999	0.90526	0.23356
C12	0.12287	0.88542	0.17996
C13	0.39639	0.59353	0.2232
C14	0.36978	0.61095	0.16928
C15	0.87019	0.09151	0.18133
C16	0.84185	0.1073	0.12629
C17	0.64504	0.59742	0.13482
C18	0.66674	0.61549	0.06862
C19	0.11993	0.06529	0.0241
C20	0.14605	0.07854	–0.0472
C21	0.41696	0.41824	0.35707
C22	0.39111	0.39677	0.33494
C23	0.87033	0.91906	0.40519
C24	0.84104	0.90003	0.37925
C25	0.4079	0.57033	0.04578
C26	0.38172	0.5878	–0.00803
C27	0.87007	0.05993	0.02393
C28	0.84205	0.07573	–0.03111
C29	0.637	0.44014	0.05442
C30	0.65802	0.41952	0.00128
C31	0.07409	0.91913	0.05186
C32	0.09702	0.89955	–0.00127

N33	0.53198	0.46626	0.26901
N34	0.52305	0.55222	0.20095
N35	–0.01399	0.95993	0.28869
N36	–0.01119	0.03978	0.13365
C37	0.24259	0.78087	–0.31837
C38	0.2839	0.7797	–0.32791
C39	0.30351	0.75468	–0.32875
C40	0.70719	0.26704	–0.31709
C41	0.74607	0.25843	–0.33822
C42	0.75514	0.23076	–0.34829
N43	0.34751	0.34753	0.25974
C44	0.51302	0.42206	0.26787
C45	0.49924	0.44976	0.27785
C46	0.45934	0.45823	0.28478
C47	0.44486	0.48547	0.28099
C48	0.40295	0.49229	0.28266
C49	0.42916	0.43577	0.26987
C50	0.37661	0.39235	0.22507
C51	0.35325	0.36825	0.19382
C52	0.32893	0.32415	0.21284
C53	0.34793	0.29867	0.22282
C54	0.33398	0.27619	0.16288
C55	0.29998	0.2782	0.09524
C56	0.27909	0.30312	0.09481
C57	0.29337	0.32593	0.15199
N58	0.78088	0.88192	0.13232
C59	0.9591	0.91727	0.31145
C60	0.95108	0.94617	0.31694
C61	0.91424	0.95915	0.33594
C62	0.90689	0.98783	0.32432
C63	0.869	1.00056	0.35012
C64	0.87842	0.94097	0.33162
C65	0.81879	0.90237	0.27933
C66	0.79502	0.8797	0.23326
C67	0.76794	0.86063	0.0626
C68	0.79106	0.83676	0.04977
C69	0.78196	0.81774	–0.03341
C70	0.74932	0.82138	–0.10546
C71	0.72607	0.84505	–0.0911
C72	0.73554	0.86461	–0.01023
C73	0.72576	0.21057	–0.33773
C74	0.68631	0.21944	–0.33228
C75	0.67724	0.24711	–0.32216
C76	0.2826	0.72984	–0.31982
C77	0.24123	0.73104	–0.32594

C78	0.22165	0.75604	–0.32528
N79	0.67711	0.64044	–0.22305
C80	0.53819	0.59373	0.13018
C81	0.55412	0.56763	0.16225
C82	0.59381	0.55904	0.15006
C83	0.60943	0.53297	0.17706
C84	0.65055	0.5254	0.15941
C85	0.61992	0.57829	0.08376
C86	0.66386	0.6151	–0.05
C87	0.68294	0.63678	–0.11595
C88	0.69242	0.6648	–0.27123
C89	0.66652	0.68229	–0.32985
C90	0.67727	0.70931	–0.35074
C91	0.71444	0.71931	–0.31728
C92	0.74228	0.70015	–0.27723
C93	0.73162	0.67327	–0.25399
N94	0.19614	0.10792	–0.18588
C95	0.00458	0.07491	0.01923
C96	0.01912	0.05023	0.0699
C97	0.0573	0.03863	0.05863
C98	0.06988	0.01389	0.10962
C99	0.10885	0.00177	0.09418
C100	0.08569	0.05293	–0.01912
C101	0.13904	0.08024	–0.16438
C102	0.16461	0.09827	–0.23221
C103	0.21658	0.13197	–0.2088
C104	0.19571	0.15622	–0.23619
C105	0.2123	0.18167	–0.21455
C106	0.25031	0.18399	–0.16763
C107	0.27302	0.15986	–0.15736
C108	0.25653	0.13428	–0.17643
C109	0.73901	0.7992	–0.1866
C110	0.7001	0.78948	–0.19415
C111	0.69179	0.7649	–0.24823
C112	0.2871	0.25485	0.02327
C113	0.24741	0.24679	0.01851
C114	0.23587	0.2247	–0.04751
N115	0.687	0.37094	–0.10221
C116	0.55282	0.42269	0.24934
C117	0.56514	0.45081	0.24776
C118	0.60314	0.46088	0.22003
C119	0.61361	0.48888	0.20859
C120	0.65318	0.49814	0.1793
C121	0.63163	0.44007	0.17246
C122	0.67482	0.39799	0.06424

C123	0.6901	0.37356	0.00612
C124	0.69245	0.3457	–0.15732
C125	0.67236	0.32227	–0.11812
C126	0.67573	0.29756	–0.17517
C127	0.69949	0.29505	–0.27215
C128	0.71839	0.31874	–0.31285
C129	0.71434	0.34382	–0.258
N130	0.15052	0.8634	–0.10673
C131	–0.0024	0.91456	0.27731
C132	0.01469	0.94113	0.26073
C133	0.0524	0.9467	0.21597
C134	0.06735	0.97268	0.18212
C135	0.10733	0.97638	0.13938
C136	0.07575	0.92276	0.16986
C137	0.12247	0.88265	0.0617
C138	0.14817	0.86312	0.0023
C139	0.1754	0.84543	–0.16644
C140	0.16056	0.8321	–0.26298
C141	0.18301	0.81235	–0.31789
C142	0.22143	0.80603	–0.28113
C143	0.23747	0.82168	–0.19225
C144	0.21496	0.84101	–0.13513
C145	0.26333	0.20979	–0.11227
C146	0.30284	0.21843	–0.11014
C147	0.31445	0.24047	–0.04358
C148	0.72193	0.74901	–0.29749
C149	0.75986	0.76059	–0.30274
C150	0.76821	0.78514	–0.2482
N151	0.34188	0.63222	–0.11721
C152	0.49823	0.59296	0.14648
C153	0.48822	0.56637	0.18926
C154	0.44974	0.55606	0.20837
C155	0.44036	0.52937	0.24589
C156	0.40018	0.51942	0.26034
C157	0.41679	0.57357	0.16119
C158	0.36316	0.60905	0.05219
C159	0.34151	0.63068	–0.00815
C160	0.32852	0.65589	–0.17353
C161	0.34298	0.6818	–0.14111
C162	0.33002	0.70528	–0.19574
C163	0.30204	0.70394	–0.28351
C164	0.28968	0.67784	–0.3199
C165	0.30324	0.6541	–0.26749
N166	0.79404	0.11514	–0.15071
C167	0.96592	0.07739	0.05309

C168	0.95558	0.055	0.12549
C169	0.91839	0.05059	0.17723
C170	0.90838	0.02899	0.25141
C171	0.86982	0.02595	0.30415
C172	0.88531	0.06788	0.1296
C173	0.82804	0.10009	0.01821
C174	0.80206	0.11872	–0.04484
C175	0.77226	0.13542	–0.20905
C176	0.78763	0.14606	–0.31031
C177	0.77096	0.16931	–0.35879
C178	0.73767	0.18192	–0.31043
C179	0.7195	0.16875	–0.21818
C180	0.73646	0.14593	–0.16749
N181	0.46683	0.50856	0.26326
N182	0.58813	0.51062	0.21152
N183	0.93103	0.00612	0.2714
N184	0.04599	–0.00387	0.16803
O185	–0.34229	0.2025	–0.31701
C186	–0.32653	0.17203	–0.30896
C187	–0.35348	0.15553	–0.22509
O188	–0.33766	0.12508	–0.2168
C189	–0.36447	0.10862	–0.13246
C190	–0.34855	0.0782	–0.12382
O191	–0.37527	0.06178	–0.03915
C192	–0.35934	0.03136	–0.03046
O193	–0.21904	0.27787	–0.37973
C194	–0.23612	0.30156	–0.30463
C195	–0.20038	0.32071	–0.26678
O196	–0.21744	0.34436	–0.19144
C197	–0.18167	0.36342	–0.15308
C198	–0.1987	0.38701	–0.07737
O199	–0.1629	0.406	–0.03868
C200	–0.17993	0.42957	0.03709
C201	–0.64138	0.04415	–0.41817
O202	–0.62384	0.07271	–0.38051
C203	–0.65567	0.08782	–0.30544
C204	–0.63809	0.11635	–0.26762
O205	–0.66987	0.13143	–0.19231
C206	–0.65221	0.1599	–0.15398
C207	–0.68392	0.17493	–0.0783
O208	–0.66617	0.20334	–0.03964
C209	–0.81365	0.42058	–0.4185
O210	–0.83045	0.39182	–0.38091
C211	–0.79824	0.37709	–0.30571
C212	–0.81508	0.34835	–0.26796

O213	–0.78291	0.33366	–0.19253
C214	–0.79985	0.30498	–0.15426
C215	–0.76776	0.29033	–0.07846
O216	–0.78477	0.26171	–0.03987
O217	–0.20211	0.74337	–0.01852
C218	–0.2161	0.71878	–0.09368
C219	–0.17804	0.70177	–0.13139
O220	–0.192	0.67722	–0.20679
C221	–0.15392	0.66029	–0.24501
C222	–0.16786	0.63581	–0.32078
O223	–0.12975	0.61895	–0.35933
C224	–0.14369	0.59449	–0.43516
C225	0.63378	0.96269	–0.41573
O226	0.61772	0.93373	–0.37813
C227	0.6503	0.91939	–0.30293
C228	0.6342	0.89045	–0.26518
O229	0.66674	0.87614	–0.18975
C230	0.65054	0.84727	–0.15148
C231	0.68301	0.833	–0.07568
O232	0.66673	0.80417	–0.03708
O233	0.21695	0.70526	–0.3789
C234	0.24492	0.68696	–0.30371
C235	0.22098	0.66065	–0.26597
O236	0.24891	0.64238	–0.19055
C237	0.2249	0.61615	–0.1523
C238	0.25277	0.59793	–0.0765
O239	0.2287	0.57176	–0.03792
C240	0.25656	0.55355	0.03793
C241	0.2356	0.95565	0.01995
O242	0.26817	0.93398	–0.01762
C243	0.24754	0.91167	–0.09279
C244	0.28013	0.89003	–0.13051
O245	0.25953	0.86775	–0.20592
C246	0.29217	0.8462	–0.24416
C247	0.27161	0.82398	–0.31993
O248	0.30429	0.8025	–0.3585
H249	0.42304	0.44693	0.09067
H250	0.3749	0.40766	0.0503
H251	0.85814	0.96381	0.18133
H252	0.80407	0.929	0.13448
H253	0.5979	0.56031	–0.0751
H254	0.63728	0.59369	–0.19651
H255	0.05056	0.04279	–0.17071
H256	0.09819	0.06798	–0.30203
H257	0.64871	0.41989	0.33208

H258	0.6881	0.38193	0.2338
H259	0.10106	0.90701	0.32925
H260	0.14315	0.87046	0.23072
H261	0.40136	0.59568	0.31785
H262	0.35214	0.62762	0.2187
H263	0.88104	0.09794	0.26897
H264	0.82867	0.12696	0.16776
H265	0.64816	0.59853	0.23034
H266	0.68815	0.63167	0.10868
H267	0.12697	0.0647	0.11814
H268	0.17523	0.08896	–0.01332
H269	0.42855	0.42183	0.44659
H270	0.38095	0.38214	0.40565
H271	0.88833	0.91711	0.48738
H272	0.8344	0.88189	0.43924
H273	0.4234	0.55244	–0.00214
H274	0.37531	0.58482	–0.10167
H275	0.88188	0.0396	–0.01541
H276	0.83029	0.06886	–0.11732
H277	0.62307	0.45819	0.00389
H278	0.66166	0.42007	–0.09416
H279	0.05257	0.93335	0.00145
H280	0.09503	0.89729	–0.09669
H281	–0.00581	0.0205	0.18418
H282	0.33859	0.7538	–0.3371
H283	0.78824	0.22384	–0.36615
H284	0.49019	0.40396	0.27622
H285	0.37899	0.47536	0.30111
H286	0.33941	0.36801	0.10552
H287	0.3756	0.29613	0.28147
H288	0.35077	0.25517	0.16867
H289	0.24893	0.3052	0.04578
H290	0.27545	0.34655	0.1488
H291	0.93392	0.90159	0.33479
H292	0.84499	0.98869	0.40174
H293	0.78858	0.85994	0.28453
H294	0.81823	0.8325	0.10854
H295	0.80186	0.79814	–0.04421
H296	0.69799	0.84895	–0.14702
H297	0.71589	0.88445	–0.00425
H298	0.6433	0.25348	–0.31791
H299	0.18646	0.75603	–0.33073
H300	0.55977	0.61074	0.09712
H301	0.67405	0.54197	0.13294
H302	0.70452	0.65138	–0.06766

H303	0.63551	0.67417	–0.36172
H304	0.65491	0.72383	–0.39689
H305	0.77577	0.7066	–0.26231
H306	0.75556	0.65784	–0.22005
H307	0.02586	0.08774	–0.03732
H308	0.13423	0.01404	0.04939
H309	0.15741	0.10388	–0.32346
H310	0.16398	0.15499	–0.27771
H311	0.19427	0.20157	–0.23581
H312	0.30697	0.161	–0.13197
H313	0.27626	0.11461	–0.16528
H314	0.65865	0.75689	–0.25361
H315	0.20211	0.2179	–0.05054
H316	0.57072	0.40224	0.23794
H317	0.68417	0.48691	0.17083
H318	0.70526	0.35623	0.05803
H319	0.65297	0.32356	–0.03816
H320	0.65877	0.27811	–0.14369
H321	0.73766	0.31761	–0.39306
H322	0.72918	0.36338	–0.29574
H323	0.01698	0.89496	0.26106
H324	0.13118	0.95886	0.14791
H325	0.16678	0.84712	0.05274
H326	0.12875	0.83774	–0.29767
H327	0.16944	0.80123	–0.39444
H328	0.27091	0.81849	–0.16468
H329	0.22874	0.85341	–0.06209
H330	0.34843	0.24677	–0.04511
H331	0.80089	0.79391	–0.25523
H332	0.47844	0.61211	0.1259
H333	0.36858	0.53	0.25628
H334	0.32355	0.64685	0.0431
H335	0.36584	0.68358	–0.06838
H336	0.34225	0.72675	–0.16944
H337	0.26782	0.67588	–0.3948
H338	0.29367	0.63267	–0.30138
H339	0.94194	0.09417	0.03224
H340	0.84639	0.04383	0.30126
H341	0.78821	0.13736	0.0022
H342	0.81468	0.13521	–0.35387
H343	0.78488	0.17813	–0.43954
H344	0.68918	0.17709	–0.18241
H345	0.72122	0.13563	–0.09148
H346	0.96382	0.00533	0.2441
H347	–0.29309	0.17201	–0.27862

H348	–0.32835	0.1617	–0.39542
H349	–0.38691	0.15551	–0.25554
H350	–0.35172	0.1659	–0.13868
H351	–0.39793	0.10852	–0.16264
H352	–0.36265	0.11907	–0.04623
H353	–0.31506	0.0783	–0.09394
H354	–0.35049	0.06772	–0.20999
H355	–0.36025	0.02111	–0.11719
H356	–0.37977	0.01901	0.02999
H357	–0.3262	0.03144	0.00224
H358	–0.2517	0.29204	–0.22748
H359	–0.25965	0.31443	–0.35421
H360	–0.18486	0.33026	–0.34392
H361	–0.17682	0.30782	–0.21738
H362	–0.16607	0.37304	–0.22997
H363	–0.15817	0.35045	–0.10382
H364	–0.2144	0.37738	–0.00062
H365	–0.22211	0.40003	–0.12672
H366	–0.20068	0.42009	0.10464
H367	–0.19858	0.44497	–0.01613
H368	–0.15313	0.4411	0.07886
H369	–0.62563	0.0266	–0.36886
H370	–0.63542	0.04111	–0.51199
H371	–0.67607	0.04361	–0.40155
H372	–0.6625	0.07436	–0.22827
H373	–0.68541	0.09133	–0.35503
H374	–0.63134	0.12983	–0.34478
H375	–0.60831	0.11284	–0.21821
H376	–0.64544	0.17346	–0.23089
H377	–0.62241	0.15629	–0.10471
H378	–0.69077	0.16133	–0.00154
H379	–0.71367	0.17861	–0.12766
H380	–0.8121	0.42133	–0.51435
H381	–0.78138	0.42378	–0.38212
H382	–0.83503	0.43792	–0.38687
H383	–0.79176	0.39063	–0.22852
H384	–0.76841	0.37393	–0.35519
H385	–0.82149	0.3348	–0.34514
H386	–0.84495	0.35151	–0.21866
H387	–0.80627	0.29134	–0.2312
H388	–0.82973	0.30824	–0.10511
H389	–0.76125	0.304	–0.00167
H390	–0.73792	0.287	–0.12771
H391	–0.23284	0.72736	–0.17089
H392	–0.23789	0.7046	–0.04418

H393	–0.16134	0.69314	–0.05419
H394	–0.15621	0.71596	–0.1807
H395	–0.13714	0.6516	–0.16805
H396	–0.13217	0.67457	–0.29418
H397	–0.18473	0.64449	–0.39758
H398	–0.18951	0.62148	–0.27152
H399	–0.13023	0.57396	–0.40182
H400	–0.1323	0.5982	–0.52473
H401	–0.17888	0.59318	–0.43505
H402	0.63585	0.96332	–0.51152
H403	0.66572	0.96644	–0.37806
H404	0.61165	0.97974	–0.38544
H405	0.65643	0.933	–0.22574
H406	0.68021	0.91658	–0.35241
H407	0.62814	0.87682	–0.34236
H408	0.60426	0.89326	–0.21588
H409	0.64448	0.83355	–0.22842
H410	0.62059	0.85017	–0.10233
H411	0.68916	0.84675	0.00111
H412	0.71293	0.83002	–0.12492
H413	0.25476	0.69952	–0.22651
H414	0.27349	0.68033	–0.35319
H415	0.21121	0.64806	–0.34316
H416	0.19237	0.66729	–0.21667
H417	0.2151	0.60348	–0.22924
H418	0.1963	0.62288	–0.10314
H419	0.26267	0.61063	0.00029
H420	0.28131	0.59113	–0.12575
H421	0.2872	0.54946	–0.00651
H422	0.24057	0.5327	0.05584
H423	0.26253	0.56502	0.12062
H424	0.21039	0.94463	0.07031
H425	0.22134	0.96605	–0.05736
H426	0.25095	0.97232	0.07472
H427	0.23351	0.92226	–0.16999
H428	0.22216	0.90058	–0.0433
H429	0.2941	0.87941	–0.05331
H430	0.30554	0.90113	–0.17982
H431	0.3062	0.8355	–0.1672
H432	0.31753	0.85739	–0.29332
H433	0.25748	0.83469	–0.39674
H434	0.24632	0.81273	–0.27068

Supplementary Table 9. BET specific surface area values of DEG-HEP-COF, DEG-COF, HEP-COF and DEG+HEP-COF were recorded at different intervals of reaction time.

COF	$S_{BET\text{-}12\ h}$ ($m^2\ g^{-1}$)	$S_{BET\text{-}24\ h}$ ($m^2\ g^{-1}$)	$S_{BET\text{-}48\ h}$ ($m^2\ g^{-1}$)	$S_{BET\text{-}72\ h}$ ($m^2\ g^{-1}$)
DEG-HEP-COF	0	30	100	1130
DEG-COF	100	440	530	920
HEP-COF	0	0	0	20
DEG+HEP-COF	0	100	120	280

Supplementary Table 10. Drug loading of COF materials in different antibiotic concentrations

Antibiotic concentration ($g\ mL^{-1}$)	Antibiotic	Drug loading ($\mu g\ mg^{-1}$)			
		DEG-COF	HEP-COF	DEG-HEP-COF	DEG+HEP-COF
0.5%	Lev	12.1	52.9	25.6	32.0
	Van	101.3	25.6	54.9	43.8
1.0%	Lev	20.4	124.3	77.6	46.0
	Van	171.1	33.1	126.1	54.9
2.0%	Lev	21.5	275.5	179.2	48.2
	Van	278.8	40.1	314.1	59.3

Supplementary References

[1]. Cai, X. et al. Molecularly defined interface created by porous polymeric networks on gold surface for concerted and selective CO_2 reduction. *ACS Sustainable Chem. Eng*. **6**, 17277–17283 (2018).

[2] Bettelhein, A., White, B. A., Raybuck, S. A. & Murray, R. W. Electrochemical polymerization of amino-, pyrrole-, and hydroxy-substituted tetraphenylporphyrins. *Inorg. Chem*. **26**, 1009–1017 (1987).

[3] Kuhnert, N., Lopez-Periago, A. & Rossignolo, G. M. The Synthesis and conformation of oxygenated trianglimine macrocycles. *Org. Biomol. Chem*. **3**, 524–537 (2005).

[4] Bryan, Z. J., Hall, A. O., Zhao, C. T., Chen, J. & McNeil, A. J. Limitations of using small molecules to identify catalyst-transfer polycondensation reactions. *ACS Macro Lett*. **5**, 69–72 (2016).

[5] Krebs, F. C. & Jørgensen, M. High carrier mobility in a series of new semiconducting PPV-type polymers. *Macromolecules* **36**, 4374–4384 (2003).

[6] Wang, T. et al. Facile one-pot synthesis of glycoluril-based porous organic polymers. *Polymer* **60**, 26–31 (2015).
