# Supplementary material for: Synthesis of a covalent organic framework with hetero-environmental pores and its medicine co-delivery application
Source: Nat Commun. 2023 Sep 28;14:6049. doi: 10.1038/s41467-023-41622-x (PMC10539374; doi:10.1038/s41467-023-41622-x)
Supplement: Supplementary file 3 — Reporting Summary [file 41467_2023_41622_MOESM3_ESM.pdf]

nature portfolio

Corresponding author(s): Ruitao Cha, Xuesong Ding, Shengbin Lei, and Bao-Hang Han

Last updated by author(s): 24/8/23

Reporting Summary

Nature Portfolio wishes to improve the reproducibility of the work that we publish. This form provides structure for consistency and transparency in reporting. For further information on Nature Portfolio policies, see our Editorial Policies and the Editorial Policy Checklist.

Statistics

For all statistical analyses, confirm that the following items are present in the figure legend, table legend, main text, or Methods section.

n/a	Confirmed	
☐	☒	The exact sample size (*n*) for each experimental group/condition, given as a discrete number and unit of measurement
☐	☒	A statement on whether measurements were taken from distinct samples or whether the same sample was measured repeatedly
☐	☒	The statistical test(s) used AND whether they are one- or two-sided<br>*Only common tests should be described solely by name; describe more complex techniques in the Methods section.*
☐	☒	A description of all covariates tested
☒	☐	A description of any assumptions or corrections, such as tests of normality and adjustment for multiple comparisons
☐	☒	A full description of the statistical parameters including central tendency (e.g. means) or other basic estimates (e.g. regression coefficient) AND variation (e.g. standard deviation) or associated estimates of uncertainty (e.g. confidence intervals)
☐	☒	For null hypothesis testing, the test statistic (e.g. *F*, *t*, *r*) with confidence intervals, effect sizes, degrees of freedom and *P* value noted<br>*Give P values as exact values whenever suitable.*
☒	☐	For Bayesian analysis, information on the choice of priors and Markov chain Monte Carlo settings
☒	☐	For hierarchical and complex designs, identification of the appropriate level for tests and full reporting of outcomes
☒	☐	Estimates of effect sizes (e.g. Cohen's *d*, Pearson's *r*), indicating how they were calculated

Our web collection on statistics for biologists contains articles on many of the points above.

Software and code

Policy information about availability of computer code

Data collection	Xeuss SAXS/WAXS system (Xenocs, France), Bruker DMX-400 NMR spectrometer (Bruker, Germany), Perkin-Elmer Spectrum One spectrometer (Perkin-Elmer Instruments Co. Ltd, USA), Pyris Diamond thermogravimetric/differential thermal analyzer (Perkin-Elmer Instruments Co. Ltd, USA), Bruker Advance III 400 spectrometer (Bruker, Germany), FEI Sirion-200 or Hitachi high technologies (S-8200), Thermo ESCALAB 250Xi analyzer (Thermo Fisher Scientific Inc., USA), 2020 volumetric adsorption analyzer (Micromeritics Instrument Corporation, USA), Lambda-950 UV-vis-NIR spectrometer (Perkin Elmer Instruments Co. Ltd, USA)), FluoroMax+ fluorometer (HORIBA, USA), ESCALAB250Xi (Thermo Scientific, American), Confocal laser-scanning microscopy (Eclipse C1, Nikon, Japan), Microtome (RM2016, Leica, Germany), and Multimode scanning tunneling microscope (Bruker, Germany).
Data analysis	Excel 2020 (Micsoft Office, USA), Origin 2018 (OriginLab, USA), ImageJ (National Institutes of Health, USA), or IBM SPSS Statistics 25 (International Business Machines Corporation, USA).

For manuscripts utilizing custom algorithms or software that are central to the research but not yet described in published literature, software must be made available to editors and reviewers. We strongly encourage code deposition in a community repository (e.g. GitHub). See the Nature Portfolio guidelines for submitting code & software for further information.

Data

Policy information about availability of data

All manuscripts must include a data availability statement. This statement should provide the following information, where applicable:

- Accession codes, unique identifiers, or web links for publicly available datasets
- A description of any restrictions on data availability
- For clinical datasets or third party data, please ensure that the statement adheres to our policy

All data are available in the main text or the Supplementary Information. Source data are provided with this paper.

Human research participants

Policy information about studies involving human research participants and Sex and Gender in Research.

Reporting on sex and gender	N/A
Population characteristics	N/A
Recruitment	N/A
Ethics oversight	N/A

Note that full information on the approval of the study protocol must also be provided in the manuscript.

Field-specific reporting

Please select the one below that is the best fit for your research. If you are not sure, read the appropriate sections before making your selection.

☒ Life sciences ☐ Behavioural & social sciences ☐ Ecological, evolutionary & environmental sciences

For a reference copy of the document with all sections, see nature.com/documents/nr-reporting-summary-flat.pdf

Life sciences study design

All studies must disclose on these points even when the disclosure is negative.

Sample size	For all these experiments, three independent repeats were deployed in each group. Sample size was determined based on mean data from our preliminary tests.
Data exclusions	No data was excluded from the analyses.
Replication	All experiments were repeated from at least three independent tests, and all attempts at replication were successful.
Randomization	All experiments were performed randomly in our study.
Blinding	Investigators were blinded to group allocation during data collection and analysis.

Reporting for specific materials, systems and methods

We require information from authors about some types of materials, experimental systems and methods used in many studies. Here, indicate whether each material, system or method listed is relevant to your study. If you are not sure if a list item applies to your research, read the appropriate section before selecting a response.

Materials & experimental systems

n/a	Involved in the study
☒	☐ Antibodies
☐	☒ Eukaryotic cell lines
☒	☐ Palaeontology and archaeology
☐	☒ Animals and other organisms
☒	☐ Clinical data
☒	☐ Dual use research of concern

Methods

n/a	Involved in the study
☒	☐ ChIP-seq
☒	☐ Flow cytometry
☒	☐ MRI-based neuroimaging

Eukaryotic cell lines

Policy information about cell lines and Sex and Gender in Research

Cell line source(s)

Human umbilical vein endothelial cells (HUVECs) were purchased from Guangzhou Xiangbo Biotechnology Co. Ltd., China. L929 cells were obtained from Beijing Dongge Boye Biotechnology Co. Ltd., China.

Authentication

Human umbilical vein endothelial cells (HUVECS) and L929s cells authentications have performed using STR (Short Tandem Repeat) analysis to confirm their identities and uniqueness.

Mycoplasma contamination

All the cells were tested and not contaminated with mycoplasma.

Commonly misidentified lines
(See ICLAC register)

No commonly misidentified cell line was used in this study.

Animals and other research organisms

Policy information about studies involving animals; ARRIVE guidelines recommended for reporting animal research, and Sex and Gender in Research

Laboratory animals

Sprague Dawley (SD) rats (6–8 weeks old, 150 g, female) were obtained from Vital River Laboratory Animal Center (Beijing, China) and raised in a specific pathogen-free environment.

Wild animals

The study did not involve wild animals.

Reporting on sex

There were no experimental data related to gender in the study.

Field-collected samples

The study did not involve samples collected from the field.

Ethics oversight

All animal studies were approved by the Animal Care and Use Committee of National Center for Nanoscience and Technology (Approval number: NCNST21-2210-0601)

Note that full information on the approval of the study protocol must also be provided in the manuscript.
